# Promoter-targeted small RNA duplexes increase *MBNL1* transcription and mitigate myotonic dystrophy-associated spliceopathy

**DOI:** 10.1093/nar/gkaf756

**Published:** 2025-08-20

**Authors:** Nikola Musiała-Kierklo, Patrycja Plewka, Adam Jasiok, Ewa Stępniak-Konieczna

**Affiliations:** Laboratory of RNA Biology, Department of Biochemistry and Biotechnology, Poznan University of Life Sciences, Dojazd 11, 60-632 Poznan, Poland; Doctoral School of Natural Sciences, Adam Mickiewicz University, Uniwersytetu Poznanskiego 2, 61-614 Poznan, Poland; Laboratory of RNA Biology, Department of Biochemistry and Biotechnology, Poznan University of Life Sciences, Dojazd 11, 60-632 Poznan, Poland; Laboratory of RNA Biology, Department of Biochemistry and Biotechnology, Poznan University of Life Sciences, Dojazd 11, 60-632 Poznan, Poland; Poznan University of Life Sciences Doctoral School, Collegium Maximum, Wojska Polskiego 28, 60-637 Poznan, Poland; Laboratory of RNA Biology, Department of Biochemistry and Biotechnology, Poznan University of Life Sciences, Dojazd 11, 60-632 Poznan, Poland

## Abstract

Functional depletion of Muscleblind-like (MBNL) proteins is a key trigger of myotonic dystrophy (DM)-associated alternative splicing (AltS) defects. To overcome MBNL insufficiency in DM cell models, we harnessed a conserved endogenous mechanism of RNA activation (RNAa) via rationally designed small activating RNA (saRNA) targeted to the most active promoter of *MBNL1* gene. We report on two lead saRNA duplexes that stimulated *MBNL1* transcription via an on-site mechanism that involves AGO2-mediated loading of the antisense strand onto target sequence, followed by recruitment of RNAPII and auxiliary RNAa pathway components. We demonstrate that neither the antisense lncRNA *MBNL1-AS1* overlapping *MBNL1* promoter nor promoter-associated cryptic RNAs are mechanistically involved in saRNA-induced *MBNL1* gene activation. Our data highlight putative transcription factors whose binding recruitment via identified saRNAs may affect *MBNL1* expression. Most importantly, we show that RNAa-based approach upregulates MBNL1 protein content in distinct DM cell models and corrects the AltS of multiple MBNL1-regulated biomarker exons, underscoring the feasibility of adapting saRNA into novel therapeutic designs. This is the first report that site-specific augmentation of the endogenous *MBNL1* transcription mitigates disease-associated AltS defects and as such, it offers new perspectives into therapeutic options against DM.

## Graphical Abstract

**Graphical Abstract:**
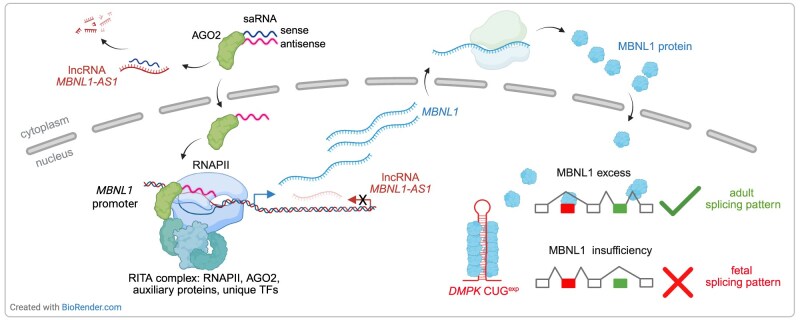

## Introduction

RNA-binding proteins of the Muscleblind-like (MBNL) family are master regulators of cellular RNA metabolism and homeostasis, including the control of alternative splicing (AltS), alternative polyadenylation, microRNA biogenesis as well as stability and localization of distinct mRNA species [1–4]. All three MBNL paralogs (MBNL1, 2 and 3) bind RNA via four zinc finger (ZnF) domains arranged in two tandems coupled by a long linker. MBNLs modulate AltS events via sequence- and position-dependent binding to cognate sequence motifs within target pre-mRNAs, relative to the regulated exon [5–7]. This positional mode of action predicts that MBNL binding within the alternative exon or upstream intronic sequences promotes exon skipping, while binding to downstream intronic regions facilitates alternative exon inclusion [2, 7, 8]. In essence, MBNL proteins repress a program of alternative exons specific of embryonic stem cells (ESCs) and fetal tissues in favor of alternative exons characteristic of differentiated tissues, which is consistent with their prominent role in promotion of differentiation and development [9–11].

The significance of MBNLs in AltS regulation is best illustrated in myotonic dystrophy (DM) type 1 (DM1), a multisystem disorder in which functional depletion of MBNL proteins from the nucleoplasm via sequestration on CUG-expanded (CUG^exp^) *DMPK* transcripts underlies massive dysregulation in the AltS of hundreds of target pre-mRNAs [9, 12, 13]. The DM1 spliceopathy, manifested as a shift in the AltS pattern from splice isoforms characteristic of adult and differentiated tissues toward those typical of ESCs and fetal tissues, results in translation of proteins inapt to perform their functions in the adult organism. Because distinct AltS events respond with different strength to the concentration of free MBNL [14], even small fluctuations in the amount of functional protein may affect the severity of the ensuing splice defects in DM1 [15]. Ultimately, functional deficiency of MBNL proteins and subsequent spliceopathy lead to pathological hallmarks of DM1 including skeletal muscle myotonia, atrophy and wasting as well as plethora of other multiorgan dysfunctions.

Current DM1 therapeutics is limited to alleviating disease symptoms and providing supportive care for the patients. Multiple experimental strategies are being extensively tested to halt the disease at distinct pathogenetic levels. These include the removal of the expanded CTG-repeated region (CTG^exp^) within the mutated *DMPK* gene (e.g. via repeat contraction [16–18], transcription inhibition [19] or CRISPR-Cas9-mediated CTG^exp^ excision [20]) as well as degradation of toxic *DMPK* transcripts (e.g. by antisense oligonucleotides directed at the repeats [21] and sequences outside the repeat region [22–26], by RNA interference [27, 28] or by engineered hU7-snRNAs [29]). Another category involves prevention of MBNL:RNA CUG^exp^ interaction or release of MBNL proteins from CUG-imposed sequestration (e.g. by RNA-directed small-molecule compounds [30–32], antisense reagents [33, 34] or engineered MBNL1-based decoy protein [35]) as well as upregulation of MBNL expression to restore the cellular pool of functional protein. The latter approach has proven successful in multiple studies grounded either on forced overexpression of various exogenous MBNL isoforms in mice, e.g. via AAV-mediated overexpression of a single *Mbnl1* [36] or *Mbnl2* [37] isoform or via constitutive multisystemic overexpression of a transgene carrying humanized version of the major *MBNL1* isoform found in adult skeletal muscles [38]. However, overexpression-based gene therapy tools may prompt excessive activity of MBNL and trigger damaging consequences. For example, adenoviral MBNL1 delivery to rat cardiac fibroblasts induced transformation and acquisition of a contractile myofibroblast phenotype [39]. Similarly, transgenic overexpression of MBNL1 *in vivo* in quiescent fibroblasts led to fibrotic response in multiple tissues, including heart, kidney, and lung, that progressed with age [39]. Further, local intramuscular overexpression of MBNL1 in mice demonstrated decreased body weight and increased muscle histopathology [40] or even reduced survival [38]. Controlled autoregulatory expression of the protein may be one way to overcome limitations associated with excessive MBNL activity, as shown by hybrid *MBNL1* overexpression construct capable of self-limiting of its own expression [41]. On the downside, overexpression of only one isoform rules out the potential effect of a balanced splice isoform diversity that could otherwise be achieved by endogenous modulation.

The therapeutic utility of endogenous *MBNL1* enhancement was demonstrated with antisense reagents including, e.g. miRNA sponges [42], antagomiRs [43], or peptide-conjugated antimiRs [44], directly targeting microRNA translational inhibitors of *MBNL1*. More recent refinement of this strategy used blockmiR-based site-specific inhibition of microRNA binding within 3′UTR of *MBNL1* mRNA [45]. Upregulation of endogenous *MBNL1* was also achieved with chemical compounds including non-steroidal anti-inflammatory drugs and unspecific epigenetic modulators (e.g. phenylbutazone [46] or HDAC inhibitors [47]).

Encouraged by these data, we hypothesized that a more directed approach utilizing a site-specific, targeted transcriptional modulation of the endogenous *MBNL1* promoter within its natural context may trigger therapeutic effect at a much lower level of the target gene upregulation compared to, e.g. exogenous overexpression, thus minimizing potential side-effects. To achieve this, we leveraged a conserved endogenous mechanism of gene activation termed RNA activation (RNAa). RNAa is a nuclear process mediated via short, naturally occurring or synthetic promoter-targeted double-stranded RNAs (dsRNA) termed small activating RNAs (saRNA) [48, 49]. RNAa activity is strictly dependent on the nuclear presence of Argonaute 2 (AGO2) protein [48, 50, 51] which, upon loading of the saRNA duplex in the cytoplasm, cleaves off and discards the passenger strand (sense, S) and forms an active complex with the guide strand (antisense, AS) complementary to cognate promoter target sequence. Following translocation to the nucleus, AGO2 docks to the saRNA-guided promoter target site and serves as a recruitment platform for the assembly of RNA-induced transcriptional activation (RITA) complex comprising auxiliary proteins, including RNA helicase A (RHA), RNA polymerase-associated protein CTR9 homolog (CTR9; part of the PAF1 complex), and DEAD-box helicase 5 (DDX5), among many other protein components. RITA interacts with RNA polymerase II (RNAPII) to initiate transcription and productive elongation of the mRNA [51]. A role of natural antisense transcripts complementary to sense genes, their promoters or regulatory regions, has been also implicated in the RNAa pathway [52]. RNAa has been effectively used for upregulation of numerous targets including, e.g. *p21^WAF1/CIP1^*, *CDH1*, *VEGF* [48], *PR*, *MVP* [49], and *CEBPA* [53]. Notably, therapeutic utility of saRNA effectors was evidenced in several cell cancer models and even clinical trials in patients with advanced liver cancer (lipid nanoparticle encapsulated saRNA drug MTL-CEBPA [53, 54]).

*MBNL1* is the most prominently expressed mammalian *MBNL* paralog and plays a major role in the AltS regulation, particularly in the context of DM1 [4, 13]. *MBNL1* expression is driven by three annotated promoters, with promoter 2 (P2) being the most prominent driver in the majority of tissues [55]. Intriguingly, P2-derived *MBNL1* pre-mRNAs undergo autoregulation through a feedback loop mechanism based on MBNL binding and exclusion of the first coding exon (e1), whose absence negatively affects the protein activity and stability due to the loss of two ZnFs encoded by e1 [55, 56]. This protective mechanism allows for self-buffering of the protein amount according to cellular demands [55, 56] and is active in DM1, though often exhausted with increasing CUG expansion size and as the disease progresses [55]. This opens up novel possibilities for therapeutic designs based on endogenous modulation of *MBNL1* expression through manipulation of P2 activity. Hypothetically, increasing the activity of P2 could augment transcription of a balanced *MBNL1* splice isoform diversity capable of undergoing autoregulation via AltS of e1 to prevent excessive protein accumulation. As such, P2 provides an attractive target to develop novel therapeutic strategies against DM1, aimed not only at increasing the cellular MBNL1 pool, but ensuring self-limiting of the protein [55, 56].

In the current work, we present a comprehensive investigation of RNAa feasibility in molecular therapy of DM1, including screening of saRNAs targeted to distinct *MBNL1* promoters and identification of the lead duplexes effectively enhancing P2-derived *MBNL1* expression in cellular models of DM1. Using chemical modifications of identified saRNAs, CUT&RUN scanning across their target sites as well as gene reporter assays, we provide molecular insights into the underlying on-site mechanism, which involves AGO2-mediated loading of the antisense strand of saRNA duplex onto target DNA and stimulation of transcription. Our data rules out the involvement of antisense lncRNA *MBNL1-AS1* overlapping *MBNL1* P2 and demonstrates that cryptic promoter-associated RNAs are unlikely to be involved in *MBNL1* RNAa. We also address the possible off-target effect of identified saRNAs and show that correct chemical modification of the sense strand can readily eliminate it without compromising RNAa. Furthermore, our study highlights putative transcription factors (TFs) whose binding recruitment via identified saRNAs may affect *MBNL1* transcription, thus underscoring the use of RNAa as a molecular toolbox to uncover novel factors in *MBNL1* expression regulation. Most importantly, we show that the two identified lead saRNAs enhance cellular MBNL1 protein content and significantly mitigate DM1-spliceopathy. In all, the ability of RNAa to selectively upregulate *MBNL1*, the key player in DM1 pathophysiology, expands possible points of therapeutic interventions for future drug developments against this rare disease.

## Materials and methods

### Design of short double-stranded saRNA duplexes

saRNA duplexes containing 19-nt sequence complementary to selected regions of the targeted promoter (+ DNA strand) were designed following a published set of guidelines [48, 57]. Target sequences within *MBNL1* promoter P2 (Eukaryotic Promoter Database (EPD) *MBNL1_1*) and P3 (EPD *MBNL1_2*) were selected between −200 and −3000 bp upstream of the EPD annotated transcription start site (TSS). A BLAT search against the UCSC Genome Browser database (GRCh38/hg38) excluded potential off-target sequences and targets falling within SNPs. Control RNA duplexes, validated and used interchangeably in all experiments, included: (i) non-targeting control saRNA (saCtrl) lacking homology to any annotated human genomic sequence (extensively validated and published by others, e.g. [48]), (ii) scrambled versions of the two lead saRNAs (saMB1_1-scrA, B or C and saMB1_2-scrA, B or C) and (iii) seed-region mutant versions of the two lead saRNAs (saMB1_1-MM4, 6 or 8 and saMB1_2-MM4, 6 or 8; with point mutations at positions 4, 6, or 8 from the 5′ end of the AS strand, designed based on [58]). 5′-end biotinylation (5′BIO) of either the sense (5′BIO-S) or the antisense (5′BIO-AS) strand of the selected saRNA duplexes was used to interfere with AGO2 function and block RNAa activity [59]. Mismatched saRNAs were designed to carry a mismatched base opposite the 5′-most nucleotide of either the sense (MM-S) or the antisense (MM-AS) strand of the duplex, in order to lower the 5′-terminus thermodynamic stability and force the selection of a given strand as the guide/leading strand [59]. saP21 was described previously [48, 51]. All saRNA duplexes were synthesized by Sigma–Aldrich/Merck and sequences are listed in Supplementary Tables S1 and S2.

### Design of siRNA and GapmeRs

Previously validated and published siRNAs targeting *MBNL1* [32], *AGO2* [59, 60], *CTR9* [51], *RHA* [51], *MEIS1* [61], and *MEIS2* [62] were purchased from Sigma–Aldrich/Merck. The *MBNL1-AS1* Lincode SMARTPool siRNA (pool of 4 siRNAs) was purchased from Dharmacon/Horizon Discovery (LOC401093), and non-targeting siCtrl duplex was purchased from Thermo Fisher Scientific (Ambion™ *Silencer*™ Select Negative Control #2 siRNA). Antisense LNA GapmeRs targeting *MBNL1-AS1* were designed using GeneGlobe Design&Analysis Hub (Qiagen) and synthesized by Qiagen. GapmerR targeting *DMPK* intron 14 was described previously [63] and purchased from Qiagen. All siRNA and GapmeR sequences are listed in Supplementary Table S3.

### Cell lines, culture conditions, and transfection of antisense reagents

Primary fibroblasts derived from DM1 patients’ skin biopsies (cell lines GM03987, GM04033, GM03132 and GM03989 expressing *DMPK* gene carrying ∼500, ∼1000, ∼1700, and ∼2000 CTG repeats, respectively) and control fibroblasts derived from a non-DM1 patient skin biopsy (cell line GM07492) were obtained from the Coriell Cell Repository at the Coriell Institute for Medical Research. Fibroblasts were grown in Eagle's minimal essential medium (EMEM) with stable glutamine (Biowest), supplemented with 10% fetal bovine serum (FBS) (Biowest), 1% antibiotic/antimycotic solution (Sigma–Aldrich/Merck) and 1% non-essential amino acids solution (Sigma–Aldrich/Merck) in 5% CO_2_ at 37°C. Normal Human Skeletal Myoblasts (HSkM) were purchased from Thermo Fisher Scientific. Non-DM1 myoblasts (9936) and DM1 patient-derived myoblasts (10009) were obtained from Telethon Biobank Network. Myoblasts were grown in Ham’s F10 medium with L-glutamine (Biowest) supplemented with 20% FBS, 0.4 μg/ml dexamethasone (Sigma–Aldrich/Merck), 10 ng/ml epidermal growth factor (Sigma–Aldrich/Merck), 1% antibiotic/antimycotic solution, in 5% CO_2_ at 37°C. HeLa and HEK293T cell lines were purchased from ATCC and Thermo Fisher Scientific, respectively, and grown in a high-glucose Dulbecco's Modified Eagle Medium (DMEM) (Biowest) with L-glutamine, supplemented with 10% FBS and 1% antibiotic/antimycotic solution in 5% CO_2_ at 37°C. For delivery of saRNAs, siRNAs or GapmeRs to fibroblasts, cells were seeded at ∼50–60% confluency and transfected 24 h post-plating using Lipofectamine RNAiMAX (Invitrogen) per manufacturer's instructions. Unless otherwise indicated, 75 nM saRNA was transfected for 120 h in all experiments. In all transfection experiments, mock refers to lipofectamine-treated cells. Experimental design for saRNA co-transfection or sequential transfection of siRNA/saRNA and GapmeRs/saRNA is detailed in the main text and figure legends. For analyses of saRNAs efficiency to upregulate *MBNL1* in cell lines other than fibroblasts (Supplementary Fig. S4), the following conditions were used. HSkM cells were seeded at ∼30–40% confluency and transfected 6 h later. After 72 h, 50% of the cells were collected and the remaining 50% cells were seeded, transfected again 6 h post-plating and harvested 72 h post-second transfection for analyses. Myoblasts lines 9936 and 10009 were seeded at ∼30% confluency and transfected 6 h later with saRNAs for 96 h. HeLa and HEK293T cells were seeded at ∼30–40% confluency and transfected ∼18 h later with saRNAs for 72 h. After 72 h, 50% of the transfected cells were collected and the remaining 50% cells were re-plated and allowed to grow for additional 24 h before harvesting for analyses.

### RNA isolation and cDNA synthesis

Cells were harvested in 500 μl TRIzol Reagent (Thermo Fisher Scientific) or Fenozol reagent (A&A Biotechnology), and total RNA was isolated with either Total RNA Zol-Out D (A&A Biotechnology) or MagnifiQ™ 16 Total RNA Plus instant kit (A&A Biotechnology) using Auto-Pure Mini (A&A Biotechnology), per manufacturer's protocol. To obtain cDNA from fibroblasts and myoblasts, 200 ng total RNA was reverse transcribed using either High-Capacity cDNA Reverse Transcription Kit with RNase Inhibitor (Thermo Fisher Scientific) or PrimeScript™ RT Reagent Kit with gDNA Eraser (Takara). To obtain cDNA from HeLa and HEK293T cells, 500 ng total RNA was reverse transcribed using TranScriba kit (A&A Biotechnology) per manufacturer's instructions.

### Gene expression analyses

Real-time quantitative reverse transcriptase polymerase chain reactions (RT-qPCRs) were performed with QuantStudio 6 and 7 Flex System (Applied Biosystems) or CFX Opus 96 Real-Time PCR System (BioRad) using PowerTrack™ SYBR Green Master Mix or PowerUp SYBR Green Master Mix (Applied Biosystems) per manufacturers’ instructions. All RT-qPCR data were analyzed in Microsoft Excel using the 2^−ΔΔCt^ method [64]. Ct values of the analyzed target gene were normalized against the reference housekeeping gene *GAPDH*. Primer sequences used for gene expression analyses are listed in Supplementary Table S4.

### Alternative splicing assays

For alternative splicing analyses, RT-PCRs were performed using GoTaq DNA Polymerase (Promega) and specific primer pairs listed in Supplementary Table S5. Reaction products were separated in 1.8–2% agarose gels with 10 mg/ml ethidium bromide or 1:20 SimplySafe (EURx). PCR images were captured and analyzed using G:Box EF2 (Syngene) and GeneTools software (Syngene), or GelDoc Go Gel Imaging System (BioRad) and Image Lab software (BioRad), respectively. Percent Spliced In (PSI) represents percentage of alternative exon inclusion calculated according to the formula: PSI = (band intensity of a PCR product with included exon × 100)/(sum of band intensities for PCR products with included as well as excluded exon).

### Actinomycin D treatment

GM04033 cells were seeded in a 6-well plate and transfected 24 h later with 75 nM indicated saRNAs or mock-treated. Actinomycin D (Sigma–Aldrich/Merck) was added directly to the medium 120 h post-transfection, at a concentration of 5 μg/ml (diluted in DMSO). Next, cells were harvested at timepoints 0, 2, 4, 6, and 8 h after actinomycin D treatment for RNA isolation and RT-qPCR-based expression analyses of *MBNL1*, lncRNA *MBNL1-AS1* and reference *GAPDH*. Results were plotted to show percentage of the remaining transcript, normalized to reference, relative to timepoint 0 (100%). Upregulation of *MBNL1* at timepoint 0 (before actinomycin D treatment) corresponding to 120 h of saRNA transfection was verified by RT-qPCR.

### Nascent RNA capture

The amount of newly synthesized RNA was determined using Click-iT™ Nascent RNA Capture Kit (Invitrogen) per manufacturer’s protocol. Briefly, cells were seeded in 6-well plates and transfected 6 h later (HEK293T) or next day (GM04033) with 75 nM saRNA for 48 h (HEK293T) or 72 h (GM04033), followed by 24 h 5-Ethynyl Uridine (EU) pulse at a concentration of 0.2 mM. Next, labeled RNA was biotinylated, captured and extracted per manufacturer’s instructions using either 1 μg EU-RNA and 0.5 mM biotin azide (HEK293T) or 400 ng EU-RNA and 0.25 mM biotin azide (GM04033). RNA bound to the beads was used as a template for cDNA synthesis using the SuperScript VILO™ cDNA synthesis kit (Invitrogen), per manufacturer’s instructions. Nascent RNA expression was quantified by RT-qPCR using *MBNL1*, lncRNA *MBNL1-AS1, DMPK*, and *GAPDH* (reference) specific primers listed in Supplementary Table S4.

### Directional RT-PCR

Directional RT-PCR analysis [65] in HeLa cells was employed to detect the presence and define the directionality of *MBNL1* P2 promoter associated RNAs. Briefly, direction-specific primer (either forward, F: GCAGATAGAAAGTTCCCATCCCT or reverse, R: GGTGCCTCTTCCCAGCGTTA) was used for reverse transcription (RT) reaction using TranScriba kit (A&A Biotechnology) and 350 ng total RNA, followed by amplification of cDNA (DreamTaq PCR MasterMix; Thermo Fisher Scientific) using both F and R primers for 35 cycles. PCR product amplified from cDNA derived from RT with F primer but not with R primer indicates antisense transcription in the analyzed region. Conversely, product amplification from cDNA derived from RT with R primer but not with F primer indicates sense transcription in the analyzed region. Amplicons obtained in both reactions indicate bidirectional transcription. PCR reactions on control “-RT” samples (containing all of the RT reaction components except for the reverse transcriptase enzyme and RT primers) were performed to exclude contamination by amplification of residual genomic DNA. Non-template control was set up to control for extraneous nucleic acid contamination.

### Generation of luciferase reporter vectors and luciferase assay

To generate the *MBNL1* promoter 2 constructs (*MBNL1* P2-Luc and P2-Luc_short), the region of *MBNL1* P2 (positions -1516/+60 and -1082/+60 relative to TSS2, respectively) was PCR amplified from HeLa genomic DNA with oligos: 5′ TTTGGTACCGGCGTTATCCTTGAGTGTAGG 3′ and 5′ TTTGCTAGCGTCAGCTCCTTGTCATGGGA 3′ (*MBNL1* P2-Luc); 5′ TTTGGTACCCTGCAGGAAGAATGTTGTACCA 3′ and 5′ TTTGCTAGCGTCAGCTCCTTGTCATGGGA 3′ (*MBNL1* P2-Luc_short) and cloned into pGL4.10[luc2] vector (Promega) using the KpnI and NheI restriction sites. The *MBNL1* P2-LucΔsaMB1_2 construct, with a deletion of the saMB1_2 target site, was prepared using Q5® Site-Directed Mutagenesis Kit (NEB) according to the manufacturer's instructions, using the following oligos: 5′ TTAAACGATTAACGCTGG 3′ and 5′ CAGTTTAAGCAAACTAAAAGAAC 3′. To generate pmirGlo_saMB1_2-S vector, oligos containing the target sequence of saMB1_2 sense (S) strand as well as an additional diagnostic KpnI restriction site were annealed and cloned 3′ off of firefly luciferase gene (*luc2*) in a pmirGLO Dual-Luciferase miRNA Target Expression Vector (Promega) in between NheI and XhoI sites. Oligo sequences were as follows: 5′ CTAGCGGTACCAAGGACTCAAGTACCCACAC 3′ (the underlined sequence marks the target sequence of the S strand of saMB1_2) and 5′ TCGAGTGTGGGTACTTGAGTCCTTGGTACCG 3′. The pmirGlo vector carries internal Renilla luciferase gene reporter for normalization of the primary reporter – the firefly luciferase. All constructs were verified by Sanger sequencing. For luciferase assay, 1.2 × 10^4^ HeLa cells/well were seeded in a 96-well plate one day before transfection. 50 ng *MBNL1* P2-Luc, *MBNL1* P2-Luc_short or *MBNL1* P2-LucΔsaMB1_2 construct was co-transfected with 2 ng pGL4.75 [hRluc CMV] (Promega) and 50 nM indicated saRNA, while 50 ng pmirGlo_saMB1_2-S vector was co-transfected with 50 nM saRNA (Lipofectamine 2000; Invitrogen). Firefly luciferase and Renilla luciferase activities were measured 48 h post co-transfection using Dual-Glo-Luciferase Assay System (Promega), per manufacturer’s instructions. Firefly activity was normalized to that of Renilla for each sample.

### Protein isolation and western blot analyses

Cells were washed in phosphate-buffered saline (PBS), harvested in 1 ml ice-cold PBS, pelleted at 5000 x g for 5 min at 4°C and lysed with 75 μl radioimmunoprecipitation assay (RIPA) buffer (Thermo Fisher Scientific) supplemented with 1X Halt Protease Inhibitor Cocktail (Thermo Fisher Scientific). Lysates were cleared by centrifugation at 15 000 x g for 15 min at 4°C. Protein concentration was determined using Pierce BCA Protein Assay Kit (Thermo Fisher Scientific) per manufacturer's protocol. Samples were mixed with Laemmli Sample Buffer (Bio-Rad) containing β−mercaptoethanol and denatured at 95°C for 5 min. Protein extracts (7–10 μg) were separated in a 10% precast polyacrylamide gel (10% Mini-PROTEAN® TGX™ Precast Protein Gels, Bio-Rad) and transferred to a polyvinylidene difluoride (PVDF) membrane (Invitrogen) for 1 h at 100 V at 4°C, using Mini-PROTEAN Tetra System (Bio-Rad). Transfer efficiency was evaluated using Ponceau S Staining Solution (Thermo Fisher Scientific) per manufacturer’s instructions. Membranes were blocked for 1 h at room temperature (RT) in 1X TBS-T buffer (tris-buffered saline [TBS], 0.1% Tween 20 [T]) containing 5% skim milk powder (Sigma–Aldrich/Merck) [1X TBS-T-MLK], then incubated O/N at 4°C with either mouse anti-human MBNL1 primary antibody (MBNL1 clone 4A8; Wolfson Centre for Inherited Neuromuscular Disease; 1:1000) or mouse anti-human HRP-conjugated Vinculin (sc-73614; Santa Cruz; 1:2500), diluted in 1X TBS-T-MLK. Membranes were washed 3 × 5 min in 1X TBS-T and incubated 1 h at RT with a secondary rabbit HRP-conjugated anti-mouse antibody (12–349, Millipore), diluted 1:10 000 in 1X TBS-T-MLK. After membrane washing, signal was detected using SuperSignal West Femto Maximum Sensitivity Substrate (Thermo Fisher Scientific). Images were captured using G:Box Chemi-XR5 (Syngene) and signal intensity was quantified using GeneTools image analysis software (Syngene).

### Cleavage under target and release using nuclease (CUT&RUN)

CUT&RUN assay (Cell Signaling) was used per manufacturer's protocol to analyze binding enrichment of specific proteins around predicted saRNA target sites during RNAa induction by selected saRNA duplexes. Briefly, GM04033 cells were seeded in 6-well plates and transfected with 75 nM saRNAs for 120 h. 1 × 10^5^ cells were harvested for each antibody reaction and additional same number of cells for input sample control. The following amounts of antibodies were used: 4 μg anti-AGO2 (PA5-117725; Invitrogen), 5 μg anti-RNAPII (clone CTD4H8; Sigma–Aldrich/Merck), 10 μg anti-RNAPII Ser2P (clone 3E10; Active Motif), 10 μg anti-RNAPII Ser5P (clone E8; Active Motif), 2 μg anti-MEIS2 (sc-515470 X; Santa Cruz) and 2 μg negative control Normal Rabbit IgG (#2729; Cell Signaling). Following manufacturer's protocol, the resulting DNA products were quantified by qPCR using 8 primer pairs (Supplementary Table S6) designed to amplify short products (60 – 80 bp) spanning genomic region from −3.5 kb to +4.2 kb relative to *MBNL1* TSS2 or −3.9 kb to +5.6 kb relative to *p21*^*WAF1/CIP1*^ TSS [51]. IP efficiency was calculated using the Percent Input Method with the following formula: % Input = 100% x 2(C[T] 100% Input Sample - C[T] IP Sample) and obtained data were plotted as % of input. Results were normalized by adding 5 ng of the Sample Normalization Spike-In DNA into each CUT&RUN reaction after DNA digestion step. For sample normalization, signal from the Sample Normalization Spike-In yeast DNA was quantified using the Sample Normalization Primer Set. The sample with the lowest C[T] value was selected to calculate the Normalization Factor (2(C[T] Selected Sample - C[T] the Other Sample)) per manufacturer's protocol. Finally, CUT&RUN reactions signals were normalized by each Normalization Factor.

### RNA fluorescent *in situ* hybridization (RNA-FISH)

For RNA-FISH, GM03989 cells were seeded into 4-well chamber slides (Nunc™ Lab-Tek™ II Chamber Slide™ System; Thermo Fisher Scientific) and transfected with 75 nM saRNAs or mock-treated for 120 h. Cells were fixed in 4% PFA/PBS for 15 min at 4°C and washed three times in 1X PBS. Pre-hybridization was performed with 30% formamide in 2X SSC for 10 min, followed by hybridization in buffer containing 30% formamide, 2X SSC, 0.02% BSA, 66 μg/ml yeast tRNA, 120 U/ml RNasin (Promega) and 2 ng/μl DNA/LNA probe (CAG)6-CA labelled at the 5′-end with Cy-3 and modified at positions 2, 5, 8, 13, 16, and 19 with LNA (FutureSynthesis). Post-hybridization washing was performed with 30% formamide in 2X SSC at 37°C for 30 min followed by 1X SSC washing at 37°C for 30 min. Slides were mounted in DAPI-containing VECTASHIELD Mounting Media with anti-fade reagent (Vector Laboratories). Images were taken on a Zeiss Axio Observer Z1 microscope with Zeiss EC Plan-Neofluar 63x/1.25 oil objective and analyzed using Zeiss ZEN blue 3.2 – Lite software. Foci were counted in *n* = 100 nuclei per experimental sample and each sample was duplicated to verify reproducibility. Negative controls included samples hybridized in the absence of (CAG)6-CA probe.

### Immunofluorescence

GM04033 cells were seeded in 2-well chamber slides (Nunc™ Lab-Tek™ II Chamber Slide™ System; Thermo Fisher Scientific) and transfected with 75 nM saRNA or mock-treated for 96 h. Cells were fixed with 4% PFA for 15 min in 1X PBS at room temperature followed by three washes in 1X PBS. Next, cells were permeabilized with 0.5% Triton X-100 in 1X PBS for 15 min followed by three washes in 1X PBS and blocked in 2.5% Normal Horse Serum (Vector Laboratories) for 30 min at RT. After blocking, cells were incubated with primary mouse antibody anti-MBNL1 (MBNL1 clone 4A8; Wolfson Centre for Inherited Neuromuscular Disease; 1:100) at 4°C O/N. Slides were washed three times in 1X PBS containing 0.1% Tween 20 (1X PBS-T) for 5 min each and then incubated for 1 h in dark with VectaFluor Duet Reagent containing anti-mouse IgG (DyLight594 - red signal) (Vector Laboratories). After two washes in 1X PBS-T, slides were mounted using DAPI-containing VECTASHIELD Mounting Media with anti-fade reagent (Vector Laboratories). Images were taken on a Nikon A1Rsi confocal microscope with Plan Apo VC 100X/1.4 Oil DIC N2 objective and analyzed using ImageJ software. Corrected total cell fluorescence (CTCF) was calculated using the formula: CTCF = Integrated density – (Area of selected cell x Mean fluorescence of background readings). CTCF was calculated from 20 different fields (confocal images taken from *n* = 2 biological replicates using the same settings in one session) containing the following total number of nuclei (n): mock, *n* = 156; saCtrl, *n* = 199; saMB1_1, *n* = 126; saMB1_2, *n* = 110.

### RNA-FISH coupled with IF (RNA-FISH-IF)

GM03989 cells were seeded into 4-well chamber slides and transfected with 75 nM saRNA or mock-treated for 120 h. RNA-FISH protocol was performed, omitting DAPI staining and mounting in the last step. Subsequently, slides were processed as described in the immunofluorescence (IF) protocol. Images of DM1 fibroblasts were taken on LEICA Stellaris 8 confocal microscope with HC PL APO 63x/1.40 OIL CS2 objective and analyzed using LAS X Office 1.4.7 software.

### Cell viability, cytotoxicity, and apoptosis analyses

ApoTox-Glo Triplex Assay (Promega) was used per manufacturer's instructions to determine cell viability, cytotoxicity and apoptosis. The assay was performed in GM04033 cells seeded in black 96-well plates with clear bottom and transfected with 25, 50, 75, or 100 nM saRNA or mock-treated for 120 h. No-cell control served as a control for background fluorescence and luminescence. Fluorescence (measured at the following wavelength sets: 400_Ex_/505_Em_ (viability) and 480_Ex_/525_Em_ (cytotoxicity)) and luminescence were determined using Tecan Infinite M200 Pro plate reader. Viability and cytotoxicity (cell fluorescence) as well as apoptosis (luminescence) were calculated as a fold change in signal intensities compared to the lipofectamine-treated cells (mock).

### Statistical analyses and data visualization

Unless otherwise indicated, experiments were repeated at least three times using a minimum of *n* = 3 independent biological replicates. Group data are presented as mean ± standard error of mean (SEM) and results obtained for each individual sample are marked on bar graphs as black dots. Two-tailed Student’s *t*-test was used for statistical analyses using GraphPad Prism 8.3.0 software with the following criteria for statistical significance: *P < 0.05; **P < 0.01; ***P < 0.001; ****P < 0.0001. Unless otherwise indicated, statistical significance is shown relative to control RNA duplex (saCtrl). All graphs were generated using GraphPad Prism 8.3.0 software.

## Results

### Small RNA duplexes targeted to *MBNL1* gene promoter increase *MBNL1* transcript levels

**Figure 1.**
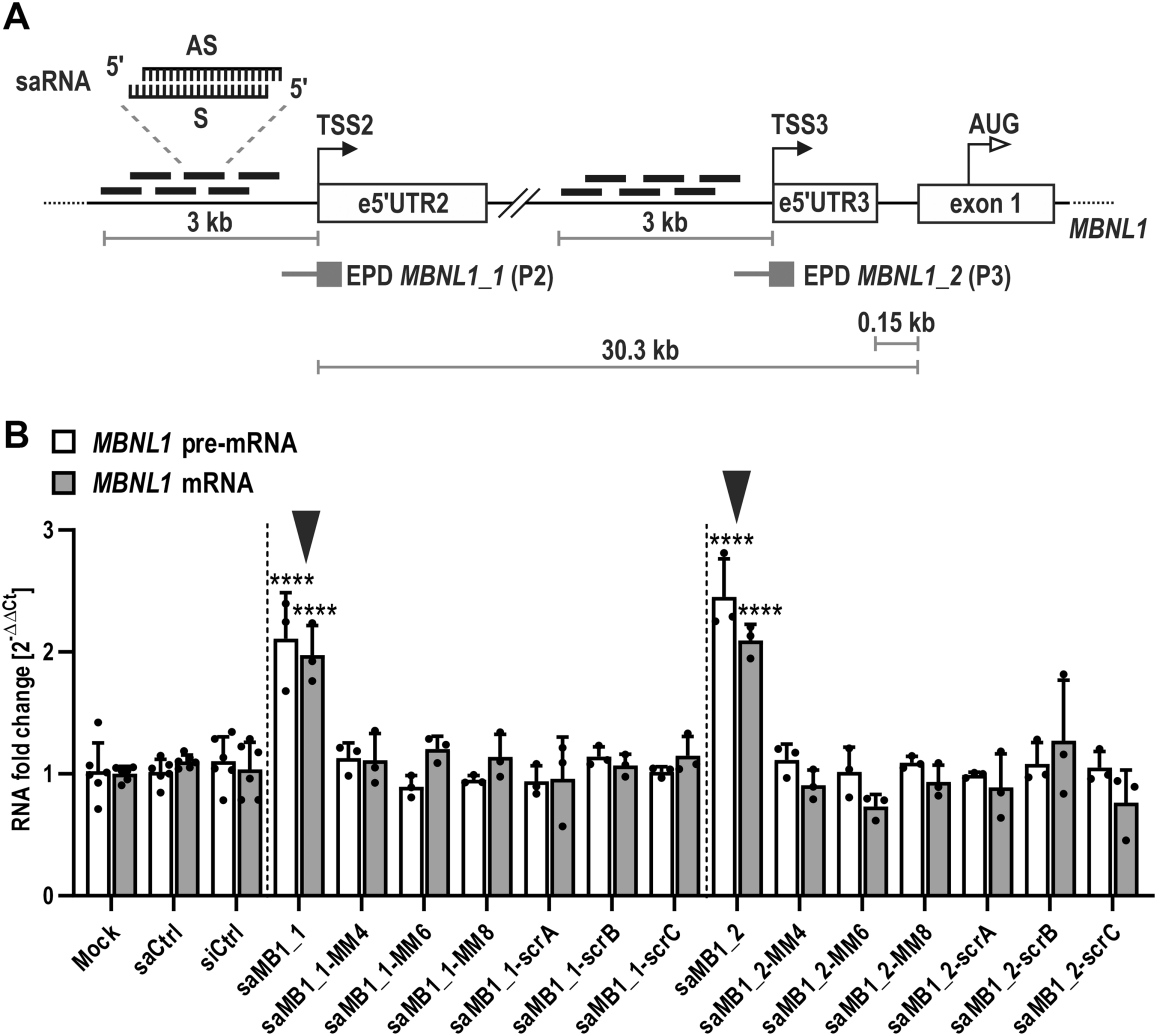
saRNA duplexes targeted to *MBNL1* gene promoter 2 upregulate *MBNL1* transcript levels in a DM1 cell model. (**A**) Schematic representation of a genomic region covering *MBNL1* promoter 2 (P2) and 3 (P3) with indicated transcription start sites (TSS2 and 3, respectively), corresponding 5′ UTR exons (e5’UTR2 and 3, respectively) as well as the first coding exon (exon 1) and translation start site (AUG). The schematic of saRNA duplex is shown with antisense (AS) and sense (S) strands marked. Genomic regions targeted by saRNAs (up to -3kb upstream of TSS2 and 3) are indicated. EPD annotated *MBNL1* promoters *MBNL1_1* (P2) and *MBNL1_2* (P3) are depicted, with the thick part representing annotated TSS and sequences downstream, and the thin part indicating sequences upstream of the TSS and defining the orientation of the promoter. (**B**) Representative results of RT-qPCR analyses of *MBNL1* pre-mRNA and mRNA levels in GM04033 DM1 fibroblasts transfected for 120 h with: lipofectamine alone (mock), 75 nM control dsRNA (saCtrl and siCtrl), saRNA against *MBNL1* P2 (saMB1_1 and saMB1_2), and mutant versions of saMB1_1 and saMB1_2 carrying a single nucleotide mismatch introduced at positions 4–8 within the seed region (MM4-8) or sequence-scrambled versions of saMB1_1 and saMB1_2 (scrA-C). Inverted arrowheads mark samples in which saRNA triggered significant *MBNL1* upregulation.

To test the feasibility of RNAa for targeted upregulation of *MBNL1*, we used human fibroblasts derived from DM1-affected patients. These primary cells constitute a natural and physiologically relevant model in which MBNL proteins, nuclear CUG^exp^ foci, and spliceopathy have been extensively studied and can be readily measured [32, 33, 41, 66], providing us with a reliable tool for assessing the therapeutic potential of saRNA duplexes in the context of DM1. We designed a set of saRNA duplexes with antisense strands complementary to genomic sequences (+ DNA strand) within *MBNL1* gene promoter (P) regions (Fig. 1A). Initial analyses included two *MBNL1* promoters annotated within the EPD as *MBNL1_1* and *MBNL1_2* (hereafter referred to as P2 and P3 [55], respectively) (Fig. 1A, Supplementary Fig. S1A–C). Promoter 1 (P1) of *MBNL1* was omitted from analyses, as mRNAs derived from TSS1 by default lack e1, and the activity of P1 is significantly lower compared to P2 and P3 [55]. RT-qPCR analyses of TSS2- and TSS3-derived *MBNL1* transcript levels in human primary fibroblasts affected by DM1 (GM04033 cell line carrying 1000 CTG repeats; Supplementary Fig. S1D) as well as meta-analyses of EPD-deposited data on promoter-specific *MBNL1* expression in skin fibroblasts from DM1 donors (Supplementary Fig. S1E) and overall promoter-specific *MBNL1* expression profile in all samples in which P2 and P3 promoters are active (Supplementary Fig. S1F), confirmed superior activity of P2 over P3. These results are concordant with our previous data [55, 56].

Initial screening of rationally designed 21-nt long (19-nt target sequence and 2-nt dT overhangs) dsRNAs targeting *MBNL1* P2 (21 saRNAs) and P3 (18 saRNAs), within the region spanning ∼3 kb upstream of the respective transcription start site (TSS2 and TSS3), revealed that the significantly more active P2 was amenable to RNAa in patient-derived primary DM1 fibroblasts, while P3 did not respond to the tested saRNAs (Supplementary Fig. S2A and B). The two top scoring P2-directed duplexes identified in initial screening, saMB1_1 and saMB1_2, targeted *MBNL1* at positions −882 and −1319 bp upstream of the EPD annotated TSS2, respectively, and enhanced *MBNL1* pre-mRNA and mRNA levels by 1.5 up to 2-fold (Fig. 1B, Supplementary Fig. S2B). None of the tested control RNA duplexes were able to induce *MBNL1* upregulation (Fig. 1B). These controls included scrambled versions of the two top-scoring saRNAs saMB1_1 and saMB1_2 (saMB1_1-scrA, B and C and saMB1_2-scrA, B and C) as well as their derivatives with point mutations introduced at distinct positions within the seed region (saMB1_1-MM4, 6 or 8 and saMB1_2-MM4, 6 or 8). Moreover, previously validated non-targeting saCtrl [51] and commercial RNA duplex (siCtrl) were used as additional controls.

The lead saRNA duplexes, saMB1_1 and saMB1_2, specifically increased the level of P2-, but not P3-derived *MBNL1* transcripts (Supplementary Fig. S2C). saRNA dose-response and RNAa kinetics analyses in primary DM1 fibroblasts indicated optimal amount of saRNA (75 nM) and timepoint (120 h) required for the most significant *MBNL1* boost (Supplementary Fig. S3A (left panel) and S3B). A comparative dose-response analysis examining the effects of saRNAs and a well-characterized siRNA against AGO2 [59, 60] at a defined dose range starting at 75 nM (saRNA response peak) up to 150 nM revealed that siRNA duplex worked similarly effective at the whole concentration range while saRNA response gradually declined above 75 nM dose (Supplementary Fig. S3A, middle and right panels). Since both analyses were performed using the same amount of transfection reagent, the limitation in saRNAs transfection efficiency arising from insufficient dsRNA to lipofectamine ratio is unlikely the cause for the observed phenomenon. In general, cytoplasmic RNAi works effectively within low concentration ranges of siRNA, depending on the targeted transcript abundance, while nuclear RNAa requires higher saRNA amounts [67]. In our study, increasing the saRNA dose above the 75 nM threshold did not enhance its response, likely because the target site, which, by default, is a sequence within the genomic DNA, may be already fully occupied or because of the chromatin context and accessibility of the *MBNL1* promoter. Our results suggest that the observed decline in saRNA response may be intrinsic to RNAa mechanism involving the two identified molecules, in which saRNA processing as well as the delayed kinetics of RNAa due to, e.g. nuclear import of saRNA and chromatin remodeling at the target promoter, may be the rate limiting steps. Broader studies involving more examples of siRNA and saRNA duplexes should examine the ubiquitousness of the observed phenomenon in the RNAa mechanism. Finally, no induction of *MBNL2* paralog was detected upon saMB1_1 and saMB1_2, supporting the specificity of the identified duplexes toward *MBNL1* (Supplementary Fig. S3C). Notably, transfection of both lead saRNAs simultaneously (data not shown) or sequentially (Supplementary Fig. S3D) failed to trigger *MBNL1* upregulation.

### saRNAs upregulate *MBNL1* RNA in distinct cell models

Both lead saRNA duplexes directed at P2 upregulated *MBNL1* RNA in distinct DM1 cell models, including patient-derived primary fibroblasts carrying *DMPK* with ∼500, ∼1700, or ∼2000 CTG triplet repeats and control non-DM1 fibroblasts derived from unaffected individuals (Supplementary Fig. S4A). Importantly, myoblasts (both unaffected and DM1) which represent the proliferating precursors of skeletal muscle tissue most affected by DM1, were also amenable to RNAa, albeit only via saMB1_2 (Supplementary Fig. S4B-C). In general, saMB1_2 induced stronger effect compared to saMB1_1 in majority of the tested cell lines, regardless of the experimental conditions (i.e. repeated transfection, as shown in Supplementary Fig. S4B). Other cell models, including HeLa (Supplementary Fig. S4D) and HEK293T cells (Supplementary Fig. S4E, left panel), were also responsive to RNAa upon either of the two lead saRNA duplexes. Long-term and persistent effect of saMB1_2 was evident in HeLa and HEK293T cells, where *MBNL1* levels remained elevated even after splitting the cells by half 72 h post-saRNA transfection and allowing the remaining cells to grow for additional 24 h (Supplementary Fig. S4D and S4E, left panel). Moreover, strong induction of nascent *MBNL1* RNA was observed upon saMB1_1 in HEK293T cells, even though other cell models were less responsive to this saRNA (Supplementary Fig. S4E, right panel).

### saRNAs increase *MBNL1* transcription at both the initiation and elongation step

**Figure 2.**
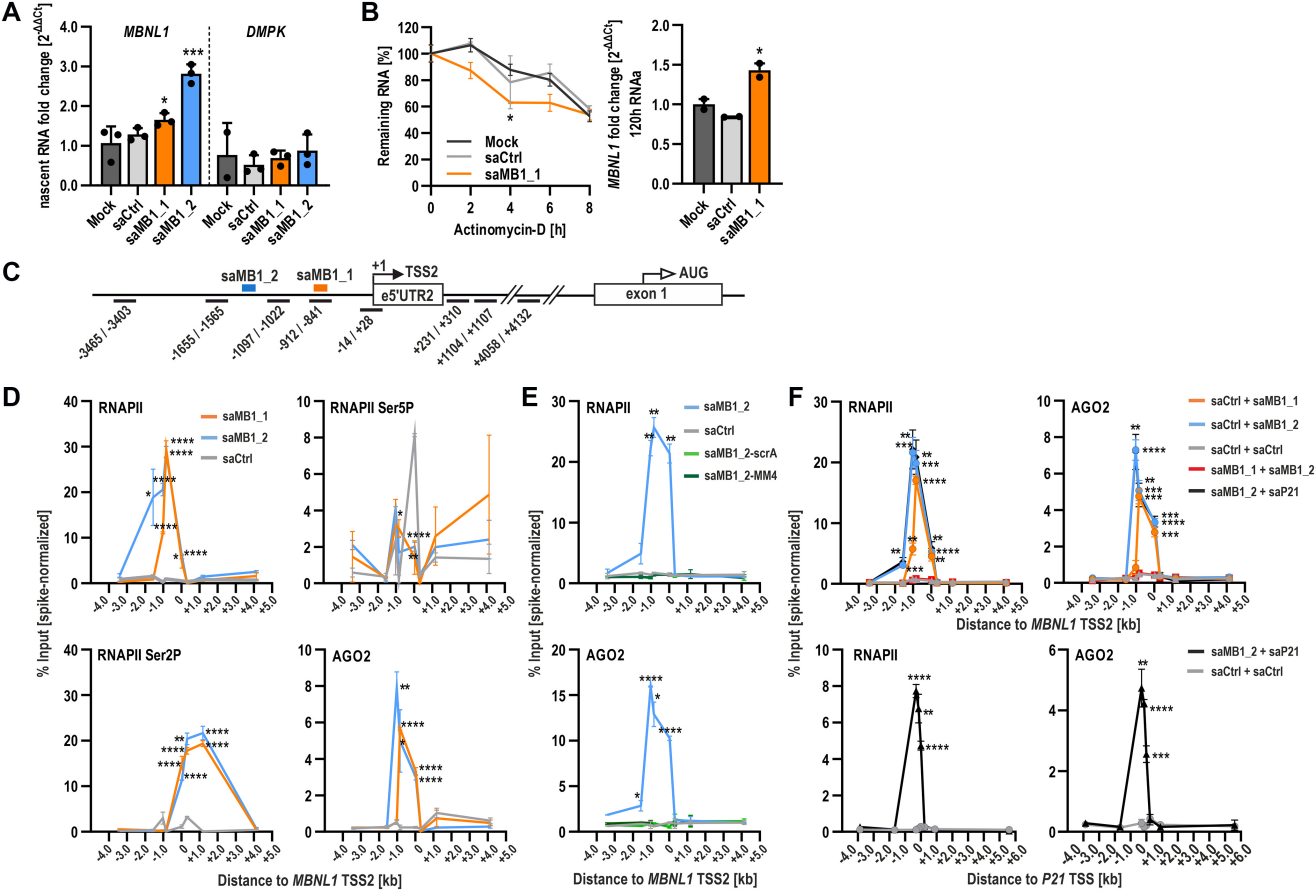
saRNA-mediated *MBNL1* upregulation occurs at the level of transcription initiation and elongation. (**A**) Nascent mRNA capture in GM04033 DM1 fibroblasts transfected with 75 nM indicated saRNA for 72 h, followed by 24 h EU pulse. Expression of nascent mRNA of *MBNL1* and *DMPK* (control) was assayed by RT-qPCR. (**B**) Line chart to the left shows representative RT-qPCR-based time course analysis (0–8 h) of the remaining *MBNL1* mRNA upon actinomycin D (Act-D) treatment of GM04033 cells transfected with 75 nM saMB1_1 (orange) or control duplex saCtrl (grey) for 120 h. Results obtained in lipofectamine-treated cells (mock, black) are shown for reference. Act-D was added 120 h post-saRNA transfection (timepoint 0 of the time course analysis). The bar graph to the right shows RT-qPCR verification of *MBNL1* upregulation 120 h post-saRNA treatment in GM04033 cells used for Act-D experiment. (**C**) Schematic representation of *MBNL1* genomic region scanned for specific protein-binding enrichment using CUT&RUN assay. TSS2 (+1) is indicated. The thick short lines indicate saRNA binding sites (blue and orange). The thin black lines below the schematic represent analyzed qPCR products and indicate their positions relative to TSS2. (**D**) Summary of the qPCR results of CUT&RUN analyses in GM04033 cells transfected with 75 nM indicated saRNA for 120 h, demonstrating binding enrichment of RNAPII, RNAPII Ser5P, RNAPII Ser2P, and AGO2 following saMB1_1 (orange) or saMB1_2 (blue) delivery. Non-targeting dsRNA (saCtrl; grey) was used as a control. (**E**) Results of the control CUT&RUN experiment in which scrambled sequence (light green) as well seed-region mutant version of saMB1_2 (dark green) failed to recruit RNAPII and AGO2, signifying sequence specificity of saMB1_2. (**F**) Summary of the qPCR results of CUT&RUN analyses in GM04033 cells transfected for a total of 120 h with indicated saRNA delivered sequentially such that 24 h treatment with 75 nM of one saRNA was followed by transfection with an equal amount of the second saRNA for additional 96 h. The order of transfection is as labelled. Binding enrichment of RNAPII and AGO2 was detected at saRNA target sites following transfection of saCtrl + saMB1_1 (orange), saCtrl + saMB1_2 (blue), saMB1_2 + saP21 (black) but not saMB1_1 + saMB1_2 (red) or saCtrl + saCtrl (grey). saP21 [48, 51] targets the promoter of *p21*^*WAF1/CIP1*^ gene at 322 bases upstream TSS. Results were normalized using Spike-In DNA and are represented as % of input. Primer sequences used for CUT&RUN analyses of the respective genes are listed in Supplementary Table S6.

The RNAa mechanism involves the activation of gene expression at the transcriptional level. Having observed that saRNA-mediated increase in *MBNL1* mRNA is accompanied by increased pre-mRNA levels (Fig. 1B), likely indicative of enhanced transcription, we corroborated these data by examining the level of nascent, newly transcribed RNAs in DM1 fibroblasts treated with saRNAs using nascent RNA capture protocol. We observed a significant increase in nascent *MBNL1* RNA, but not unrelated control *DMPK* transcripts (Fig. 2A). Similar results were obtained in HEK293T (Supplementary Fig. S4E, right panel). Altogether, these data strongly support the notion that *MBNL1* promoter-targeted saRNAs enhance *MBNL1* gene transcription. Further, to exclude the possible effect of saRNA on *MBNL1* transcript stability, we inhibited mRNA synthesis using actinomycin D (Act-D) starting at 120 h post-saMB1_1 delivery. *MBNL1* transcript decay kinetics followed a similar pattern to the one in controls at distinct timepoints after Act-D addition, thus ruling out unspecific effect of saRNA on *MBNL1* mRNA turnover (Fig. 2B).

To support these findings, we next assessed the binding of factors associated with active transcription and RNAa pathway by scanning the region around *MBNL1* TSS2 via CUT&RUN assay. Upon saRNA delivery, chromatin was immunoprecipitated with antibodies recognizing RNA polymerase II (RNAPII; corresponding to total RNA polymerase II) as well as Ser5 and Ser2 phosphorylation status of the C-terminal domain of RNAPII (RNAPII Ser5P and RNAPII Ser2P; corresponding to initiating and elongating polymerase, respectively) as well as AGO2. Subsequently, qPCR with eight sets of primers specific for *MBNL1* gene, ranging from −3.5 kb to +4.2 kb relative to *MBNL1* TSS2 (Fig. 2C schematic), was used to scan the indicated genomic region for binding of these proteins. We detected a massive binding enrichment of RNAPII and RNAPII Ser2P to *MBNL1* P2 in primary DM1 fibroblasts transfected with either of the two top scoring saRNAs (Fig. 2D). While the enrichment of RNAPII Ser2P in saCtrl-treated cells was minimal, both saMB1_1 and saMB1_2 triggered a significant increase in RNAPII Ser2P occupancy near TSS2 that extended across the transcribed region of the gene. Conversely, strong signal for RNAPII Ser5P was detected specifically around TSS2 only in saCtrl-treated cells, indicating that this region is likely poised for transcription but not actively transcribed in the absence of saMB1_1 or saMB1_2 (Fig. 2D). However, RNAPII Ser5P enriched signals were detected in saRNA-transfected cells between −1 and −2 kb from TSS2, a region covering their respective binding sites (Fig. 2D). Next, we analyzed the binding of AGO2, a crucial component of the RNAa pathway [50, 51]. In response to saMB1_1 and saMB1_2 delivery, a significant enrichment in AGO2 binding was detected in regions encompassing saRNA target sites (Fig. 2D). This enrichment corresponded to RNAPII as well as RNAPII Ser5P / Ser2P binding at the same locations and was not detected upon saCtrl. Importantly, control CUT&RUN experiments confirmed that scrambled version of saMB1_2 (saMB1_2-scrA) and seed-region mutant version of saMB1_2 (saMB1_2-MM4) recruited neither RNAPII nor AGO2, as opposed to unmodified saMB1_2 duplex (Fig. 2E). Overall, these data demonstrate strong sequence specificity of RNAa complex guidance to *MBNL1* promoter by identified saRNAs. We conclude that saRNA-AGO2 complex targets *MBNL1* P2 and associates with RNAPII to promote formation of the transcription initiation complex that stimulates both transcription initiation and productive elongation, which is concordant with the current understanding of RNAa mechanism [51].

The lack of RNAa response upon combined sequential or simultaneous treatment with saMB1_1 and saMB1_2 (Supplementary Fig. S3D and data not shown) was unexpected and could suggest that saRNAs might interfere with each other through direct interaction. However, this is unlikely considering the lack of sequence complementarity between both saRNAs. Alternatively, reduced target site accessibility due to steric hindrance while loading the saRNAs onto closely located target sequences by AGO2 may be the cause. This scenario is supported by the lack of RNAPII and AGO2 binding to the *MBNL1* promoter region upon combined saMB1_1 and saMB1_2 transfection as opposed to transfection of either of these saRNAs separately, where clear enrichment of both proteins was detected using CUT&RUN assay (Fig. 2F, upper panel). To further dissect this phenomenon, we performed CUT&RUN upon sequential transfection of saMB1_2 and a well characterized saP21 [51], targeting the *p21*^*WAF1/CIP1*^ gene promoter. As expected, a massive enrichment of RNAPII and AGO2 was detected at both target sites when saP21 and saMB1_2 were used in combination (Fig. 2F). We conclude that steric hindrance and an interference in RNAa complexes assembly at the promoter target site are most likely responsible for the lack of *MBNL1* induction when prompted by two saRNAs, saMB1_1, and saMB1_2, targeting nearby sequences.

### saRNAs stimulate transcription by recruiting specific TFs to *MBNL1* promoter

To reinforce the conclusions on the effect of saRNA on *MBNL1* transcription, we next scanned experimentally validated genome-wide TF-binding profiles in the JASPAR CORE 2024 collection and UCSC Genome Browser to search for unique TFs whose binding sites may overlap saRNA target sites. Annotated PBX2, MEIS1 and MEIS2 binding sites were found to overlap the cognate target genomic sequence of saMB1_1 (Supplementary Fig. S5A). All these TFs may act as transcriptional activators [68, 69]. To verify their possible involvement in *MBNL1* RNAa, we silenced the expression of *MEIS1* and *MEIS2* via RNAi in DM1 fibroblasts and assessed the effect on saRNA-mediated *MBNL1* enhancement. While efficient silencing of *MEIS1* and *MEIS2* prevented *MBNL1* upregulation via saMB1_1 (Supplementary Fig. S5B and C), it did not affect saMB1_2-mediated RNAa (Supplementary Fig. S5D and E), strongly suggesting specific cooperation of these TFs in transcription driven by the saRNA whose target site overlaps their respective binding sites. In support, enriched binding of MEIS2 to *MBNL1* P2 was detected by CUT&RUN scanning only upon saMB1_1 transfection, but not saMB1_2 (Supplementary Fig. S5F). In all, these data suggest that saRNA may operate via recruitment of specific transcriptional activators whose binding sites overlap its cognate sequence. This highlights a unique opportunity to identify novel *MBNL1* expression regulators using RNAa as a molecular toolbox and necessitates further studies on the role of these saRNAs in *MBNL1* regulation.

### Silencing of RNAa complex components prevents saRNA-mediated increase in *MBNL1* expression

**Figure 3.**
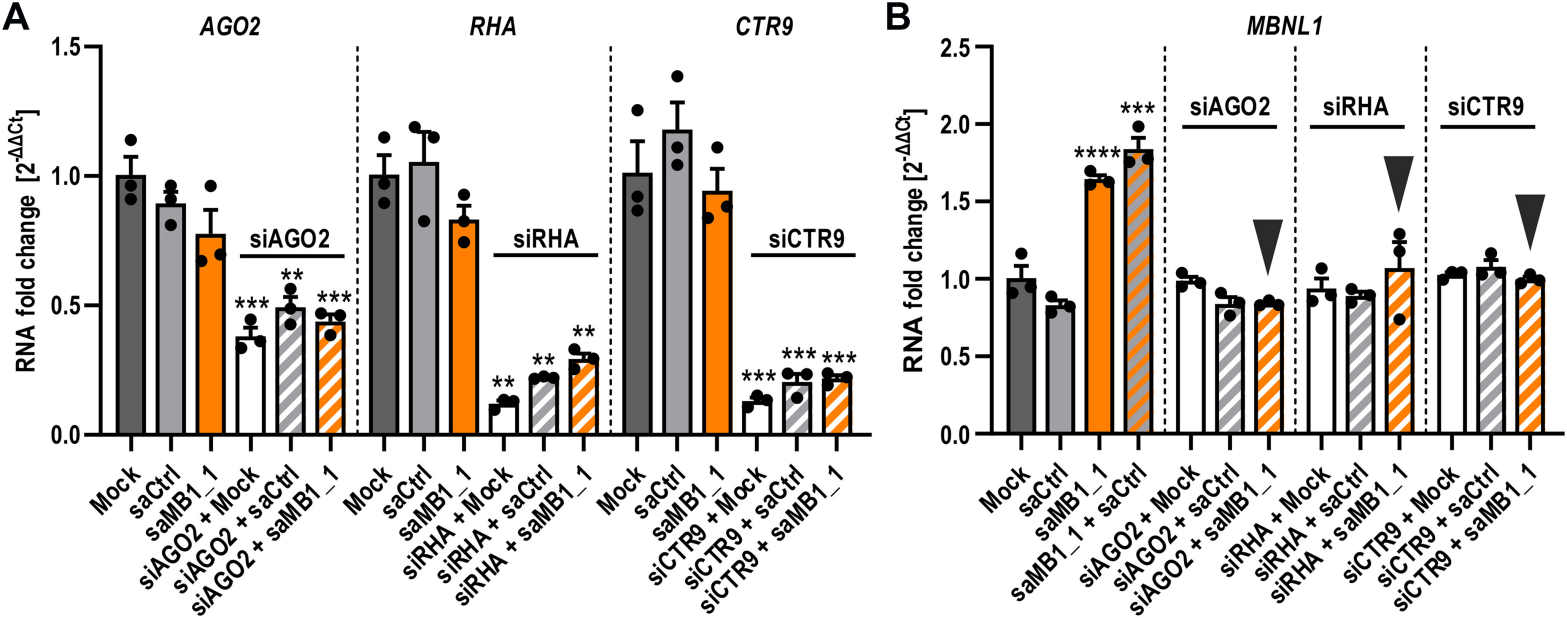
saRNA duplex upregulates *MBNL1* via RNAa pathway. (**A** and **B**) Knock-down of *AGO2*, *RHA*, and *CTR9* expression (**A**) and fold change of *MBNL1* induction (**B**) verified by RT-qPCR in GM04033 DM1 fibroblasts transfected for a total of 120 h with lipofectamine alone (mock) or 75 nM indicated dsRNA delivered solo, or sequentially such that 24 h treatment with 75 nM siRNA against indicated RNAa complex component (siAGO2, siRHA, and siCTR9) was followed by mock-transfection or transfection with an equal amount of saCtrl or saMB1_1 for additional 96 h. Inverted arrowheads in (B) indicate samples in which knock-down of the indicated RNAa complex component inhibited *MBNL1* induction.

Finally, to provide direct evidence that saMB1_1 and saMB1_2 enhance *MBNL1* transcription specifically via RNAa mechanism, we used RNA interference (RNAi) to knock-down the expression of major established RNAa pathway components required for the assembly of the RITA complex, including AGO2, RHA and CTR9 [51, 67]. siRNAs against specific RNAa pathway components were delivered into GM04033 cells either alone or sequentially, i.e. followed by transfection of saMB1_1 or saCtrl duplex. We observed that efficient knock-down of RITA components (∼50–80%) by their corresponding siRNAs (siAGO2, siRHA, siCTR9; Fig. 3A) completely blocked *MBNL1* upregulation via saMB1_1 (Fig. 3B). In contrast, combined transfection of saMB1_1 and saCtrl permitted *MBNL1* enhancement to the same extent as in saMB1_1-treated samples. These analyses were extended to saMB1_2 duplex, in which case downregulation of *AGO2* likewise prevented *MBNL1* transcriptional upregulation (Supplementary Fig. S6A and B). Cumulatively, these data support RNAa pathway involvement in saRNA-mediated transcriptional upregulation of *MBNL1*.

### Distinct mechanisms may be involved in RNAa of *MBNL1*

**Figure 4.**
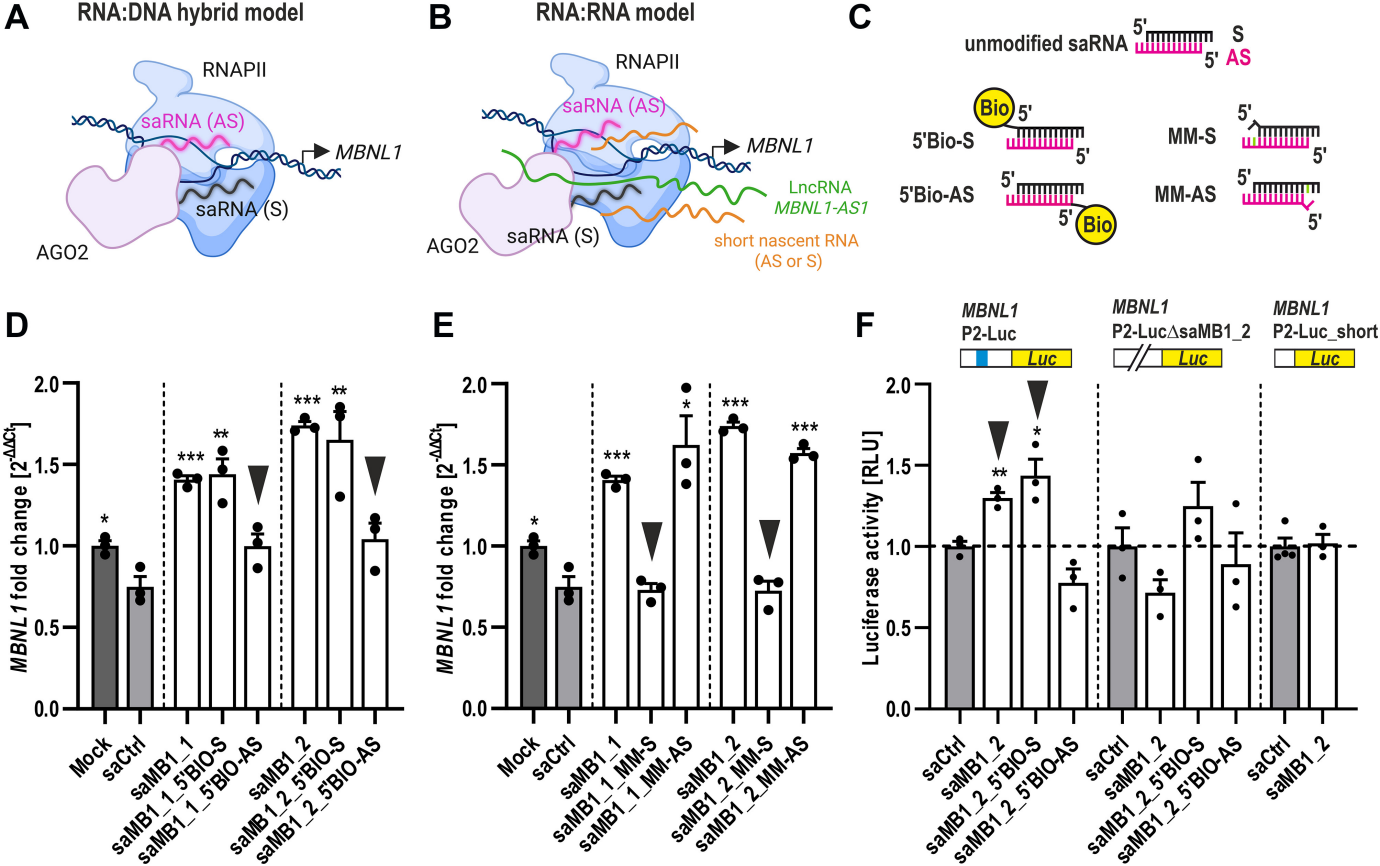
AGO2-guided antisense strand of saRNA duplex is required for *MBNL1* RNAa. (**A** and **B**) Schematic of putative RNA activation mechanism models at *MBNL1* P2 predicting the major role of either the antisense (AS) or the sense (S) strand of saRNA duplex and considering the involvement of lncRNA *MBNL1-AS1* as well as nascent cryptic promoter-associated transcripts. Created in BioRender. Stepniak-Konieczna, E. (2025) https://BioRender.com/sz1613a. The RNA:DNA hybrid model (**A**) presumes an on-site mechanism in which respective saRNA strands target their complementary DNA sequences to facilitate transcriptional complex assembly. The RNA:RNA model (**B**) predicts that lncRNA *MBNL1-AS1* and/or nascent promoter-associated transcripts in either orientation are targeted by respective saRNA strands to promote the assembly of transcriptional machinery. Detailed description in main text. (**C**) Schematic representation of saRNA modifications used to either inhibit (5′BIO) or promote (MM) the selection of indicated strands by AGO2. (**D**and **E**) RT-qPCR analyses of *MBNL1* levels assayed in GM04033 DM1 fibroblasts transfected for 120 h with 75 nM indicated unmodified saRNAs or their modified versions covalently linked to biotin at the 5′-end of either the antisense (5′BIO-AS) or the sense (5′BIO-S) strand (**D**), or modified saRNAs containing mismatched bases at the 3′end opposite to the 5′-most nucleotide of either the antisense (MM-AS) or the sense (MM-S) strand (**E**). Inverted arrowheads indicate chemical modifications of the AS (D) or S strand (E) that either blocked (D) or were incompatible with (E) *MBNL1* RNAa, respectively. (**F**) Dual-luciferase assay of *MBNL1* promoter activity in HeLa cells co-transfected with an indicated reporter vector (schematic), saRNA and Renilla reporter for normalization. saMB1_2 binding site is represented as a blue rectangle. Luciferase activities were measured 48 h post-co-transfection and are represented as RLU (relative light unites) normalized to that of Renilla for each sample.

Having verified that the two identified saRNAs stimulate *MBNL1* transcription specifically via RNAa-dependent pathway, we next investigated possible scenarios explaining how these saRNAs might operate at *MBNL1* promoter. Based on the available published data, we hypothesized two main models (Fig. 4A and B; based on [67]). The RNA:DNA hybrid model predicts that RNAa is an on-site process mediated by AGO2-dependent loading of the antisense (AS) or the sense (S) strand of saRNA duplex onto complementary target sequence within the coding (+) or template (−) DNA strand, respectively (Fig. 4A). AGO2 may recruit DNA/RNA helicases such as RHA to unwind DNA duplex and allow for RNA:DNA hybrid pairing, and other auxiliary proteins like CTR9 to stimulate active *MBNL1* transcription via assembly of the RITA complex [58, 67]. Another scenario, based on the RNA:RNA model, predicts that the sense strand (S) of saRNA duplex plays the leading role in RNAa by binding to the natural antisense long non-coding RNA (lncRNA) *MBNL1-AS1* originating from the complementary strand of *MBNL1* gene and partially overlapping the genomic P2 region (Fig. 4B). In this on-site process, association of the S strand of saRNA with the lncRNA *MBNL1-AS1* could act as a molecular scaffold for the assembly of the RITA complex and the recruitment of additional proteins, including histone modifiers, to alter local chromatin structure and ultimately drive transcription [52, 67]. Alternatively, promoter-associated short nascent cryptic transcripts in either sense or antisense orientation could also serve as docking sites for respective saRNA strands complexed with AGO2 (Fig. 4B). To investigate which of the proposed models operates at *MBNL1* P2, we first utilized chemical modifications of saRNA duplexes to verify specific strand involvement in *MBNL1* RNAa, followed by examining of the putative physical association of the individual saRNA strands with their target sites using *MBNL1* promoter-luciferase reporter assays. These studies were complemented by comprehensive analyses of lncRNA *MBNL1-AS1* requirement for RNAa-mediated upregulation of *MBNL1*. We also examined the presence of nascent cryptic transcripts at *MBNL1* promoter.

### AGO2-guided antisense strand of saRNA duplex is required for *MBNL1* RNAa

To gain insights into the saRNA strand involvement in RNAa at *MBNL1* promoter, we first leveraged chemical modifications of the lead saRNA duplexes to specifically inhibit or promote selection of either the S or the AS strand by AGO2. While modifications to the 5′-end of either strand are known to interfere with AGO2 function and block RNAa, lowering of the thermodynamic stability at the 5′-end of either strand promotes its preferential loading onto AGO2 as the guide (active) strand [59]. Accordingly, we designed chemically modified saRNAs derived from saMB1_1 and saMB1_2 that were covalently linked to biotin at the 5′-end of either the AS (5′BIO-AS) or the S (5′BIO-S) strand to inhibit their selection by AGO2 (Fig. 4C). Conversely, promotion of strand selection was achieved by introducing a mismatched base at the 3′-end of the opposite strand. This mismatched base would directly face the 5′-most nucleotide of either the AS (MM-AS) or S (MM-S) strand (Fig. 4C), thus lowering its 5′-end thermodynamic stability and marking it for preferential selection. We observed that inhibition of the AS strand in either of the two lead saRNA duplexes (5′BIO-AS) completely blocked *MBNL1* upregulation in DM1 primary fibroblasts, while inhibition of the S strand (5′BIO-S) had no effect on saRNA activity (Fig. 4D). In line with this observation, MM-S modified saRNA duplexes were not able to induce *MBNL1* upregulation (Fig. 4E), indicating that promotion of the S strand selection by AGO2 is incompatible with *MBNL1* RNAa. Conversely, promotion of the AS strand selection (AS-MM) permitted *MBNL1* RNAa to a similar level as in the case of unmodified duplexes (Fig. 4E). Altogether, these data strongly indicate that in the case of both identified saRNA duplexes, the AS strand is the leading (guide) strand selected by AGO2 during RNAa, while the S strand has no impact on RNAa activity.

### saMB1_2 binds to the target region of the *MBNL1* gene promoter

To verify whether the AS strand of saMB1_2 may bind the intended target within the *MBNL1* gene promoter, we assessed the enrichment in *MBNL1* promoter activity upon saRNA transfection. The *MBNL1* promoter containing saMB1_2 binding site was cloned upstream of the luciferase gene to generate the *MBNL1* P2-Luc construct (Fig. 4F, schematic). This reporter was co-transfected into HeLa cells with either saCtrl, unmodified saMB1_2 or modified saMB1_2 linked to biotin at the 5′-end of either the sense (5′BIO-S) or the antisense (5′BIO-AS) strand. A dual-luciferase reporter assay showed that both the unmodified as well as the 5′BIO-S version of the saMB1_2 can significantly activate the *MBNL1* P2-Luc reporter compared to the saCtrl and 5′BIO-AS, thus reinforcing the lead role of the antisense strand of saMB1_2 in *MBNL1* RNAa (Fig. 4F). To confirm that *MBNL1* activation by saMB1_2 is mediated by the physical interaction with complementary site within *MBNL1* promoter, we generated *MBNL1* P2-LucΔsaMB1_2 construct with a deletion of saMB1_2 binding site (Fig. 4F, schematic). Notably, saMB1_2 and its 5′BIO-S version were unable to trigger an increase in the luciferase activity when co-transfected with *MBNL1* P2-LucΔsaMB1_2 (Fig. 4F). These results indicate that saMB1_2 can directly interact with *MBNL1* promoter. Consistently, saMB1_2 failed to stimulate the luciferase activity when co-transfected with a reporter containing a shorter fragment of the *MBNL1* promoter, deprived of the larger region encompassing saMB1_2 target site (*MBNL1* P2-Luc_short) (Fig. 4F). These data reinforce the sequence-specificity of saMB1_2 for the predicted binding site and suggest that during the RNAa process saMB1_2 binds to the genomic DNA of the target site within the *MBNL1* promoter to facilitate its transcriptional stimulation. Altogether, these findings point to the RNA:DNA hybrid model of *MBNL1* RNAa, but do not exclude other players potentially serving as additional docking sites for identified saRNAs.

### LncRNA *MBNL1-AS1* is dispensable for RNA activation of *MBNL1*

**Figure 5.**
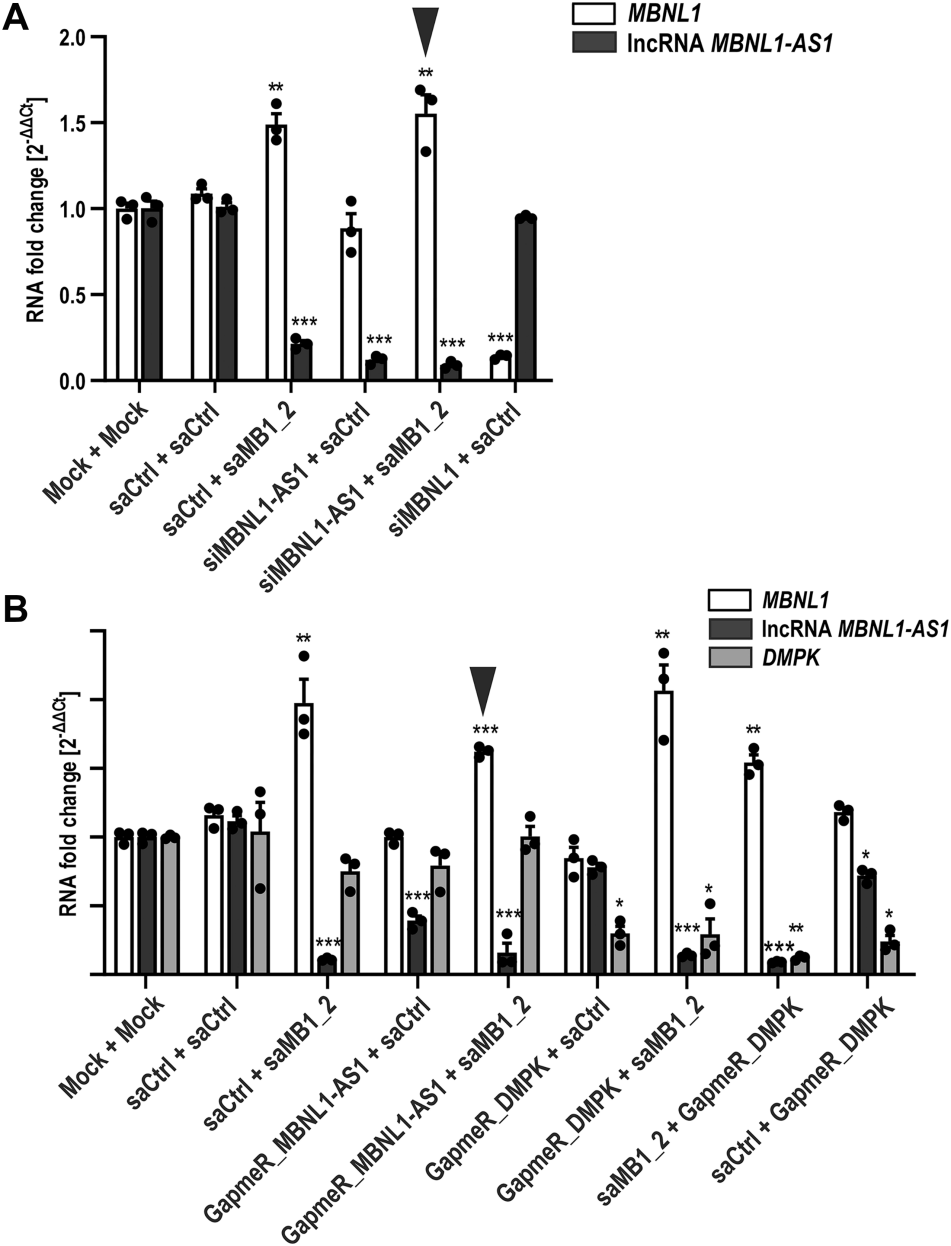
LncRNA *MBNL1*-*AS1* is dispensable for RNAa of *MBNL1*. (**A** and **B**) RT-qPCR analyses of indicated transcript levels in GM04033 DM1 fibroblasts subjected to silencing of lncRNA *MBNL1-AS1* using RNA interference (**A**) or GapmeR-mediated knock-down (**B**). In (A), cells underwent two successive transfections including mock or 75 nM indicated dsRNA (saCtrl, saMB1_2, siMBNL1-AS1, or siMBNL1) for 24 h, followed by second transfection, including mock or 75 nM indicated dsRNA (saCtrl, saMB1_2) for additional 96 h. In (B) cells were mock-transfected or transfected with 75 nM indicated reagent (saCtrl, saMB1_2, GapmeR against *MBNL1-AS1* or *DMPK*) for 24 h, followed by additional 96 h mock-transfection or transfection with 75 nM indicated dsRNA (saCtrl and saMB1_2) or GapmeR against *DMPK*. Samples were harvested for analyses after a total of 120 h. Order of transfected reagents is as labeled. Inverted arrowheads indicate samples in which silencing of *MBNL1-AS1* using RNAi (A) or GapmeRs (B) failed to interfere with RNAa of *MBNL1*.

Because antisense transcripts overlapping sense genes have been implicated in RNAa of their sense counterparts, we set out to investigate whether the lncRNA *MBNL1-AS1* overlapping *MBNL1* P2 participates in saMB1_2-mediated RNAa at *MBNL1* promoter, as predicted in the RNA:RNA model (Fig. 4B). For this purpose, we silenced *MBNL1-AS1* via classical RNAi as well as via GapmeR-mediated RNaseH-dependent degradation. Two distinct approaches were undertaken to eliminate lncRNA *MBNL1-AS1* isoforms from both cytoplasmic (RNA interference, Fig. 5A) as well as nuclear compartment (GapmeR-mediated degradation, Fig. 5B). Remarkably, knock-down of *MBNL1-AS1* had no inhibitory effect on *MBNL1* upregulation upon subsequent transfection of saMB1_2, regardless of the silencing approach (Fig. 5A and B). Further, silencing of *MBNL1-AS1* by itself did not alter *MBNL1* expression level, demonstrating that this lncRNA unlikely serves as a negative regulator of its sense counterpart. Overall, these findings rule out the requirement for *MBNL1-AS1* in *MBNL1* RNAa and reinforce the conclusion that *MBNL1* upregulation is the sole result of saRNA-dependent transcriptional enhancement.

Importantly, the RNA:RNA model predicts that promoter-associated cryptic transcripts (in both orientations) may also serve as binding sites for saRNAs and thus may be involved in RNAa (Fig. 4B). We tested this hypothesis via directional RT-PCR analyses [65] which allowed us to determine the presence and orientation of potential short transcripts deriving from the saMB1_2 target region of the *MBNL1* P2 promoter. Obtained data clearly indicate that only antisense (most likely corresponding to *MBNL1-AS1*) but not sense RNAs are produced at the analyzed region of the *MBNL1* promoter (Supplementary Fig. S7A and B). Lack of sense amplicons likely mirrors enhanced transcription of the full length *MBNL1* RNA and interference with the transcription of nascent promoter-associated RNAs. Although these data are not quantitative, the intensity of bands representing antisense amplicons was clearly reduced upon saRNA transfection (Supplementary Fig. S7B). Collectively, these findings provide evidence that neither the lncRNA *MBNL1-AS1* nor the promoter-associated cryptic transcripts are mechanistically involved in *MBNL1* RNAa via the identified saRNAs. Furthermore, they support the conclusion that saRNAs target the *MBNL1* promoter via an on-site mechanism, in which the AS strand of the saRNA duplex binds complementary *MBNL1* DNA coding strand to facilitate transcriptional stimulation of *MBNL1* promoter.

### LncRNA *MBNL1-AS1* is downregulated upon RNA activation of *MBNL1*

The comprehensive study on *MBNL1-AS1* involvement in RNAa of the *MBNL1* gene revealed an intriguing and consistent downregulation of lncRNA *MBNL1-AS1* at distinct timepoints following delivery of saMB1_1 and saMB1_2 to DM1 fibroblasts (Supplementary Fig. S8A), which is consistent with the results of directional RT-PCR. Sequence-scrambled versions of saRNAs were unable to reduce lncRNA *MBNL1-AS1*; however, varying degrees of reduction were observed with control saRNA duplexes carrying mutations at distinct positions within the seed region (Supplementary Fig. S8B). This implies that the mechanism underlying *MBNL1-AS1* reduction does not require perfect sequence complementarity. Downregulation was evident even upon transfection of saRNAs that failed to enhance *MBNL1* but were targeted to genomic sites overlapping *MBNL1-AS1* (Supplementary Fig. S8C). We hypothesized that this effect could be driven by the S strand of saRNA duplex via classical RNAi. Alternatively, saRNA-mediated assembly of the transcriptional complex at *MBNL1* promoter could interfere with the antisense transcription. Intriguingly, knock-down of AGO2, a crucial component in both RNAi as well as RNAa, did not prevent *MBNL1-AS1* reduction upon saRNA, thus ruling out the former scenario (Supplementary Fig. S9A). In contrast, the level of nascent, newly synthesized *MBNL1-AS1* was significantly downregulated upon saMB1_1 or saMB1_2, thus pointing to the latter possibility (Supplementary Fig. S9B). Moreover, when transcription was blocked with actinomycin D following saRNA delivery, we detected a significant reduction in the remaining lncRNA *MBNL1-AS1* compared to controls, pointing to its decreased stability (Supplementary Fig. S9C). In all, these data allow us to conclude that at least two distinct mechanisms participate in lncRNA *MBNL1-AS1* reduction upon effective RNAa of its sense counterpart. On the one hand, reduced transcription of *MBNL1-AS1*, which stems likely from transcriptional collision with the complex transcribing *MBNL1*. On the other hand, reduction in lncRNA stability, which likely mirrors the secondary miRNA-like effect of the S strand of saRNA. Prompted by these results, we used chemically modified saRNA duplexes to verify the hypothesis that the S strand drives downregulation of *MBNL1-AS1*. Strikingly, AS strand inhibition by 5′-end biotinylation (5′BIO-AS) had no impact on *MBNL1-AS1* reduction, while 5′BIO-S modification completely inhibited it in the case of saMB1_1, but not saMB1_2 (Supplementary Fig. S10A). Similarly, the saRNA harboring a single 3′-most nucleotide mismatch within the S strand (MM-AS) prevented *MBNL1-AS1* decrease, but only in the case of saMB1_1 and not saMB1_2 (Supplementary Fig. S10B). In all, these data demonstrate that *MBNL1-AS1* downregulation is controlled by the S but not the AS strand of saMB1_1 duplex. Conversely, saMB1_2 duplex is either more tolerant to modifications of the 5′ (in 5′BIO-S) as well as 3′ end (in MM-AS) of the S strand or alternatively, *MBNL1-AS1* downregulation in this case may be triggered by an additional factor which remains to be identified.

### The off-target potential of the S strand of saMB1_2 can be eliminated by chemical modification

The S strands of the identified saRNA duplexes, when incorporated into AGO2 complex, may lead to off-target effects by interacting with complementary non-specific transcripts or gene promoters. To assess the off-target potential of the S strand of saMB1_2, we generated pmirGlo_saMB1_2-S reporter vector, in which the target sequence of the S strand was cloned into the 3′UTR of the firefly luciferase gene within a dual-luciferase reporter vector pmirGlo, which also carries Renilla luciferase gene for normalization (Supplementary Fig. S10C, schematic). We hypothesized that reduction in luciferase activity upon binding of the S strand to luciferase mRNA, due to destabilization of its mRNA and blocked translation, would be indicative of the miRNA-like off-target potential of the S strand. As predicted, a significant loss in reporter activity was observed in HeLa cells co-transfected with pmirGlo_saMB1_2-S and saMB1_2 or its modified version carrying 5′-biotin on the AS strand (5′BIO-AS), but not on the S strand (5′BIO-S) (Supplementary Fig. S10C, chart). The latter allowed robust luciferase luminescence, underscoring the miRNA-like off-target potential of the S strand. Notably, these data also indicate that correct chemical modification of the S strand can diminish this unanticipated off-target effect without compromising saRNA activity to upregulate the target gene.

### saRNA duplexes increase MBNL1 protein content in myotonic dystrophy cell models

**Figure 6.**
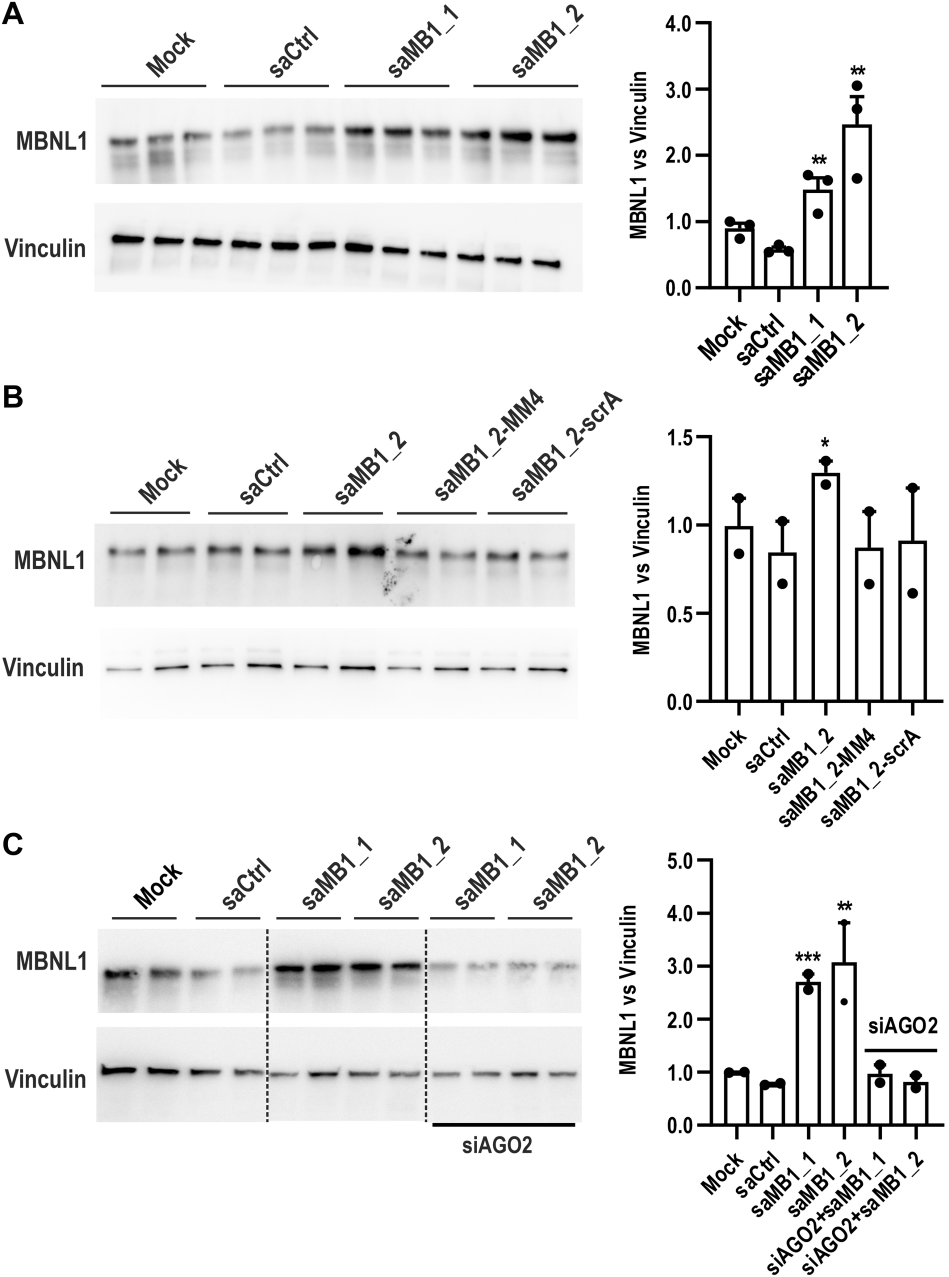
RNA activation enhances MBNL1 protein content in a cellular model of myotonic dystrophy. (**A**-**C**) Representative western blot analyses of MBNL1 protein levels in GM04033 DM1 fibroblasts transfected with 75 nM saMB1_1 and saMB1_2 for 120 h (**A**), scrambled and seed-region mutant versions of saMB1_2 (**B**) as well as saRNAs transfected on the background of *AGO2* knock-down (siRNA against *AGO2* transfected for 24 h, followed by second transfection with the indicated saRNA for additional 96 h) (**C**). Quantifications are shown to the right of gel images. MBNL1 and Vinculin (loading control) blots are derived from the same experiment and a single gel. Data are presented as mean ± SEM of triplicate (A) or duplicate (B and C) biological repeats. Lanes in (C) were rearranged and the boundary between non-adjacent rearranged lanes is indicated by vertical dashed lines. Original uncropped scans are shown in Supplementary Fig. S11.

To evaluate the therapeutic impact of *MBNL1* RNAa, we assessed the level of cellular pool of MBNL1 protein upon saMB1_1 and saMB1_2 delivery to DM1 fibroblasts. Both lead duplexes triggered a significant increase in MBNL1 protein level ranging from 1.3- up to 3-fold at 120 h post-transfection (Fig. 6A–C and Supplementary Fig. S11A–C). Overall, these results mirror increased levels of *MBNL1* RNA detected by RT-qPCR analyses. Importantly, neither scrambled (saMB1_2-scrA) nor seed-region mutant versions (saMB1_2-MM4) of saMB1_2, which are incompatible with RNAa (Fig. 1B), were able to trigger MBNL1 protein increase (Fig. 6B and Supplementary Fig. S11B). Consistently with data demonstrating AGO2 requirement for RNAa, RNAi-mediated silencing of AGO2 prevented MBNL1 protein boost (Fig. 6C and Supplementary Fig. S11C). Moreover, time course RNAa revealed the induction of MBNL1 protein across 72 h up to 120 h post-saMB1_2 transfection, and the gradual decline to basal levels at 144 h (Supplementary Fig. S12A). Because P2-derived *MBNL1* pre-mRNAs undergo autoregulation through a feedback loop mechanism based on MBNL binding and exclusion of the first coding exon (e1), whose presence governs protein activity and stability, we also looked at the correlation of MBNL1 elevation with e1 exclusion from *MBNL1* transcripts. Indeed, a drop in e1 content corresponded with the elevated MBNL1 protein at 72–120 h, after which its inclusion returned to the basal level observed in control samples (Supplementary Fig. S12B).

**Figure 7.**
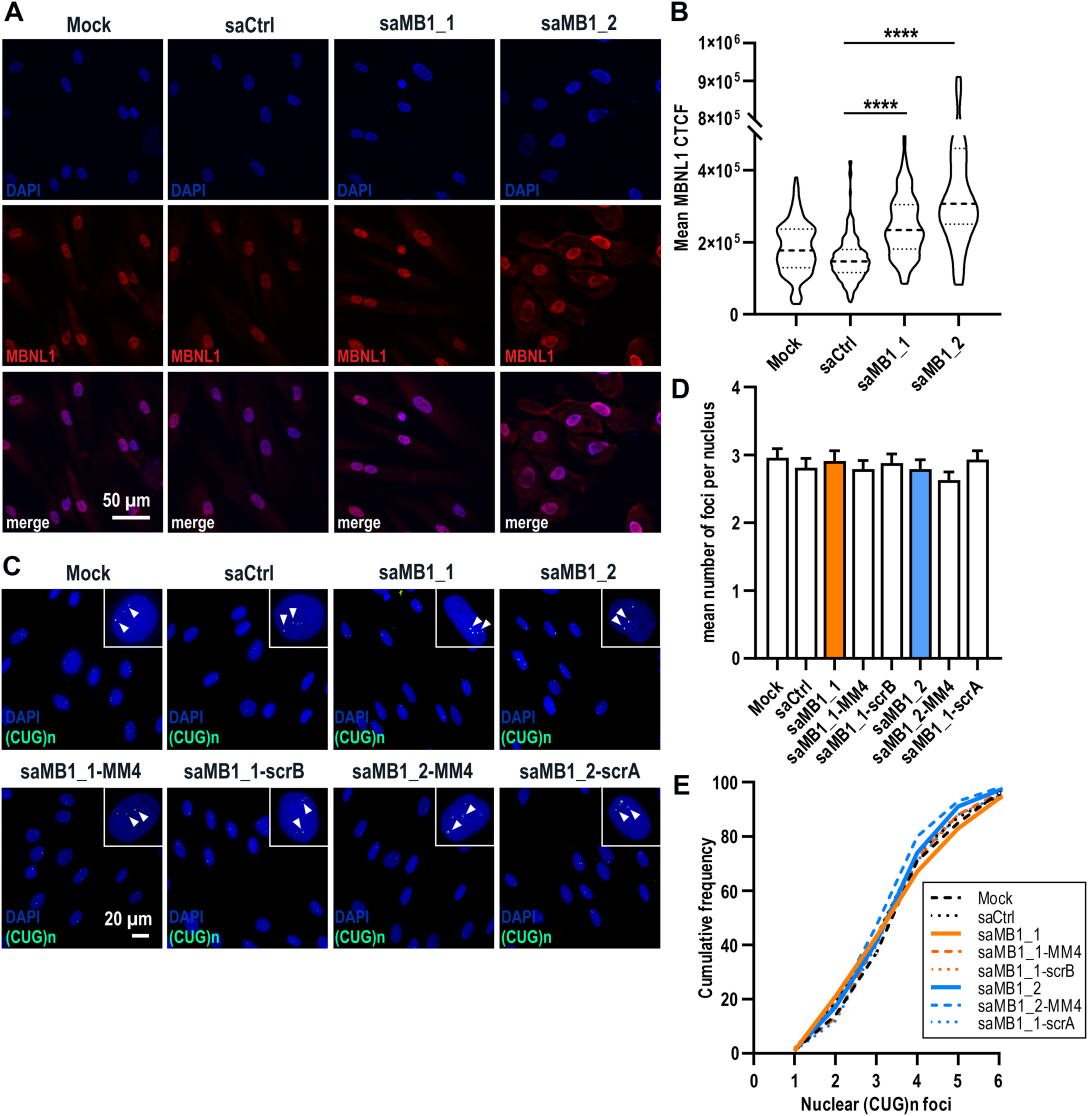
saRNA duplexes increase MBNL1 protein content in DM1 cells without enhancing nuclear RNA foci accumulation. (**A**) Representative confocal images of MBNL1 IF (red; DyLight594) in GM04033 DM1 primary fibroblasts transfected with 75 nM indicated saRNAs or mock-treated for 120 h. Nuclei were counterstained with DAPI (blue). Scale bar (50 μm) is indicated. (**B**) Quantification of total MBNL1 signal intensity and its distribution across sample types shown in (A). CTCF was calculated from 20 different fields (confocal images taken from *n* = 2 biological replicates using the same settings in one session) of the following total nuclei number (n): mock *n* = 156; saCtrl *n* = 199; saMB1_1 *n* = 126; saMB1_2 *n* = 110. Thick dashed lines indicate median and thin dotted lines indicate quartiles. (**C**) Representative RNA-FISH analyses of (CUG)n foci (green; Cy3) in GM03989 DM1 primary fibroblasts transfected with 75 nM indicated saRNAs or mock-treated for 120 h. Nuclei were counterstained with DAPI (blue). Arrowheads in blow-up panels indicate examples of nuclear RNA foci. Scale bar (20 μm) is indicated. (**D**and **E**) Quantification of the mean number of RNA foci per nucleus (D) and cumulative frequency of nuclear CUG^exp^ RNA aggregates (E) in sample types shown in (C). Data in (D and E) are presented as mean ± SEM of *n* = 100 nuclei/sample type.

In agreement with the protein data, IF analyses of MBNL1 in DM1 cells 120 h post-saRNA delivery revealed significantly higher intensity of MBNL1 signal in nuclei of saMB1_1- and saMB1_2-transfected cells compared to controls (Fig. 7A and B). Because excessive MBNL1 levels could possibly enhance RNA foci formation, we quantified CUG^exp^ foci in saRNA-treated DM1 fibroblasts. RNA fluorescent *in situ* hybridization analyses (RNA-FISH) clearly indicated similar numbers of nuclear RNA inclusions across all samples, regardless of the treatment type (mock and control duplexes vs. saMB1_1 and saMB1_2) (Fig. 7C–E). Moreover, co-localization of MBNL1 with RNA inclusions was unaltered in saMB1_1 and saMB1_2-transfected cells, as visualized by RNA-FISH coupled with immunofluorescent (IF) detection of MBNL1 protein (RNA-FISH-IF), and did not seem to lead to increase in foci number or size (Supplementary Fig. S13).

### *MBNL1* RNAa corrects alternative splicing defects in DM1 fibroblasts and myoblasts

**Figure 8.**
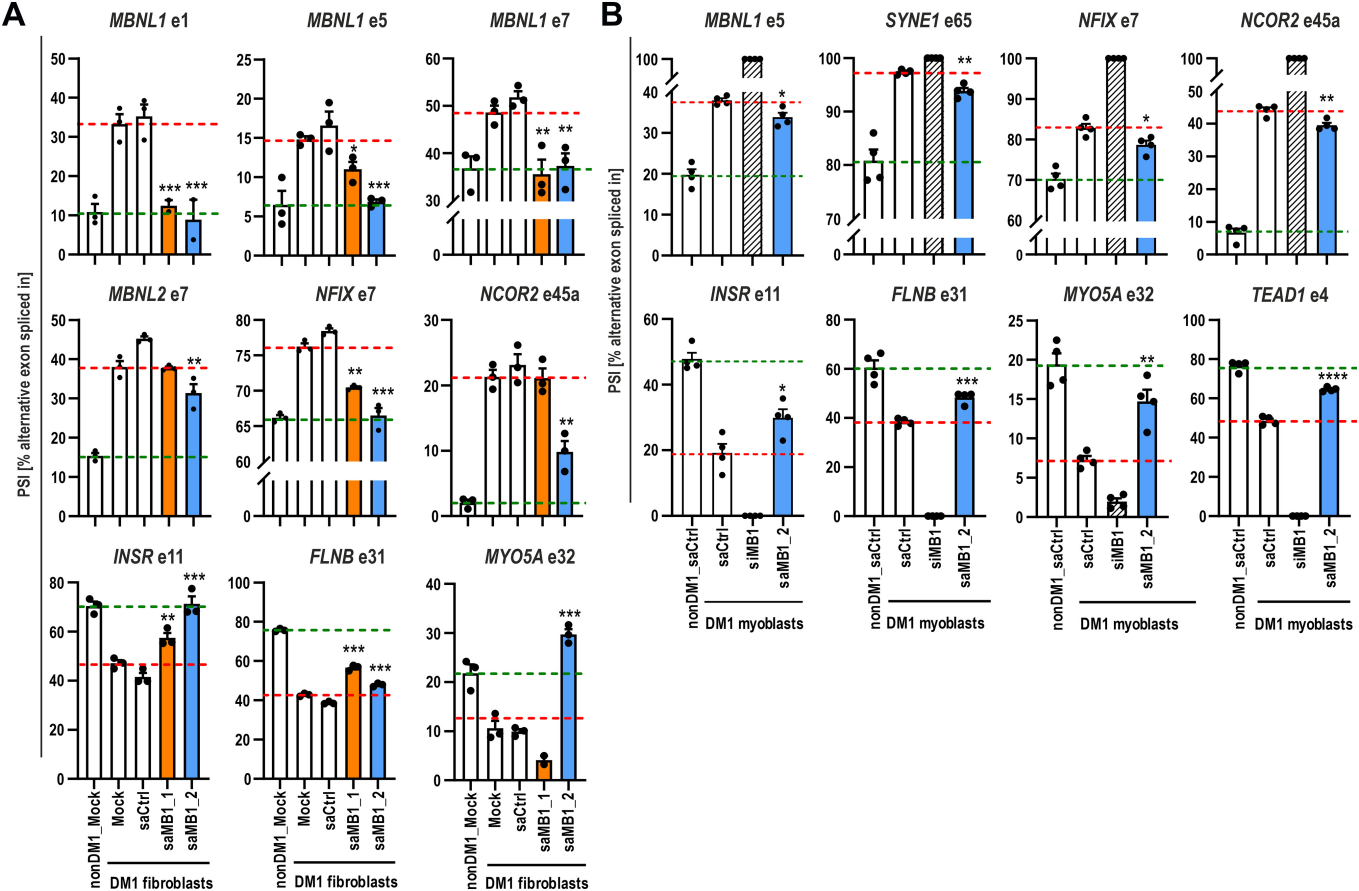
RNA activation of *MBNL1* mitigates DM1-spliceopathy. (**A**-**B**) RT-PCR-based quantification of indicated alternative splicing events in GM04033 DM1 fibroblasts (**A**) and DM1 10009 myoblasts (**B**) transfected with 75 nM indicated saRNA for 120 and 96 h, respectively. NonDM1_Mock (A) and nonDM1_saCtrl (B) represent unaffected fibroblasts transfected with lipofectamine or saCtrl, respectively. Red and green dashed lines mark the level of alternative exon inclusion in DM1 and unaffected cells, respectively. Data are presented as mean ± SEM of triplicate (A) and quadruple (B) samples and refer to % alternative exon inclusion (PSI; % exon spliced in). For each splicing event, quantification refers to a single representative experiment, which was repeated three times for consistency. Representative RT-PCR gel images used for quantification are shown in Supplementary Figs S14 and S15.

Because even small fluctuations in the cellular pool of MBNL1 may affect the outcome of the overall alternative splicing pattern in diseased cells, we investigated whether the increase in MBNL1 content following RNAa was sufficient to correct the AltS defects in DM1 cell models including patient-derived fibroblasts (Fig. 8A, Supplementary Fig. S14) and myoblasts (Fig. 8B, Supplementary Fig. S15). We assessed the splicing of alternative exons negatively as well as positively regulated by MBNL1, i.e. exons whose inclusion is repressed by MBNL1 (*MBNL1* e1, e5, e7; *MBNL2* e7*; NFIX* e7; *NCOR2* e45a; *SYNE1* e65) or promoted by MBNL1 (*INSR* e11; *FLNB* e31; *MYO5A* e32; *TEAD1* e4), respectively. These selected splicing events are altered in diseased cells in the absence of functional MBNL proteins and were extensively validated as biomarkers of DM1 spliceopathy [32, 66, 70]. Strikingly, delivery of either of the two lead saRNA duplexes triggered a consistent shift in the splicing pattern of the analyzed exons from the one observed in DM1 (marked by red dashed lines) toward the pattern typical of healthy, non-affected cells (marked by green dashed lines) (Fig. 8A and B). In both tested cell models saRNAs consistently decreased the inclusion of alternative exons normally repressed by MBNL1 (Fig. 8A and B). Analogous observation was made for the group of biomarker exons positively regulated by MBNL1, i.e. saRNAs enhanced their inclusion. The effects of saMB1_1 on AltS correction were generally weaker compared to saMB1_2, which is consistent with more efficient upregulation of *MBNL1* mRNA and protein via saMB1_2 (Figs 1B, 2A, and 6; Supplementary Fig. S4). In DM1 fibroblasts, the rescue effect of saMB1_2 ranged from almost a ∼100% (e.g. *MBNL1* e1 and e5, *NFIX* e7, *INSR* e11) to between 30% and 50% splicing correction (e.g. *MBNL2* e7 and *NCOR2* e45a, respectively), depending on the analyzed exon. In contrast, splicing correction in DM1 myoblasts, although evident, was much less pronounced compared to DM1 fibroblasts due to the weaker induction of MBNL1 which most likely mirrors poorer efficiency of saRNA uptake by muscle cells. Importantly, sequence-scrambled or seed region mutant saRNA duplexes had no effect on AltS of MBNL1 target exons (Supplementary Fig. S16A and B). Moreover, no adverse cellular effects were noted upon delivery of saRNA duplexes to DM1 cells, as judged by unaltered cell viability, cytotoxicity and apoptosis for up to 5 days post-transfection (Supplementary Fig. S17A–C). Overall, these data clearly demonstrate a significant therapeutic utility of RNA activation in the treatment of AltS defects in DM1 via targeted upregulation of *MBNL1* gene transcription and at the same time, necessitate further studies on efficient delivery of these therapeutics to muscle cells.

## Discussion

The fact that molecular hallmarks of DM1 stem from the depletion of MBNL proteins, but not *MBNL* genes mutations, offers a unique therapeutic possibility to compensate for protein deficiency with extra endogenous expression. This approach has been pioneered using miRNA translational repressors of transcript isoforms originating from *MBNL1* and *MBNL2* genes, which were targeted with *miR-23b* and *miR-218* antagonists to elevate MBNL1 and 2 proteins in skeletal muscles and correct DM1-like alterations ([71] and reviewed in [72]). In this work, we took an alternative approach based on a direct transcriptional stimulation of the endogenous *MBNL1* gene promoter via natural, conserved cellular mechanism of gene activation – RNAa. Our screening of rationally designed RNAa effectors, saRNA duplexes, targeted to the most active promoter of *MBNL1*, resulted in identification of the two lead molecules, saMB1_1 and saMB1_2, that enhanced *MBNL1* pre-mRNA, mRNA and protein levels *in cellulo* up to ∼2-fold. Importantly, studies utilizing distinct approaches to boost endogenous content of MBNL1 protein, including aforementioned miRNA repressors as well as e.g. generic HDAC inhibitors [47, 73] or globally acting DNA methylation inhibitors [46], demonstrated that even a relatively small increase of MBNL1 level (between 1 and 2-fold) can be beneficial in mitigating DM1 disease phenotypes. Additionally, benefits of moderate *MBNL1* increase include: (i) prevention from unwanted side-effects associated with uncontrolled MBNL1 overexpression (as shown by others, e.g. [38, 39]) and (ii) in the case of saRNA – a specific, site-directed action on *MBNL1* transcription. Mechanistically, we show that direct transcriptional stimulation of *MBNL1* promoter by saRNA is sufficient to boost the cellular pool of MBNL1 protein and counteract the AltS defects associated with MBNL1 depletion via CUG^exp^ RNA – a cellular hallmark of DM1. The level of MBNL1 protein upregulation was sufficient to significantly ameliorate spliceopathy, yet it did not trigger increased RNA foci number and size nor did it lead to cellular toxicity. This study therefore constitutes the first report that site-specific augmentation of *MBNL1* transcription may mitigate disease-associated AltS alterations in DM1 cells and hence, may foster studies on novel therapeutic designs.

Our data indicate that P2 promoter was susceptible to RNAa, while all tested P3-directed saRNAs failed to upregulate *MBNL1* expression. Because effective saRNA design is largely a “hit-or-miss” process [74], it is possible that the number of tested saRNAs and the coverage of genomic sites within P3 were not sufficient. However, it also specifically highlights the importance of promoter selection in RNAa strategies. Particularly, considering the higher occupancy of permissive histone marks around P2/TSS2 than TSS3/P3, based on UCSC Genome Browser-deposited data, as well as superior activity of P2 over P3 observed in the current and in our previous studies [55, 56]. Basal level of gene expression from a particular promoter is crucial for effective RNAa, as both the susceptibility and the magnitude of gene induction by RNAa require the target gene to be poised for transcription and manifest low to intermediate levels of mRNA expression. It is plausible that P3 was refractory to RNAa in our studies because of epigenetic chromatin modifications and lesser accessibility of this promoter region. Nonetheless, identifying effective saRNAs directed at *MBNL1* P3 would allow to directly compare the effects of *MBNL1* transcriptional stimulation from a promoter by default giving rise to transcripts containing the first coding exon (e1) and thus highly functional and stable protein, versus P2-derived RNAs that undergo autoregulation via alternative splicing of e1.

saRNA duplexes identified in our study increased nascent *MBNL1* mRNA without altering mRNA decay kinetics, strongly indicating that RNAa at *MBNL1* P2 occurs at the transcriptional level. This is consistent with published works [51, 54] and moreover, concordant with previous ChIP-based analyses [50, 51] showing saRNA-triggered enrichment of RNAPII occupancy at targeted promoter regions. Massive accumulation of initiating and elongating RNAPII at saMB1_1 and saMB1_2 target sites, extending across the gene body, strongly supported saRNA-mediated formation of the transcription initiation complex as well as effective elongation. Together with the requirement for the key RITA complex components including AGO2, RHA, and CTR9, these data clearly indicate that saMB1_1 and saMB1_2 mediate *MBNL1* transcriptional enhancement specifically via canonical RNAa pathway. An interesting observation is that two saRNAs targeting one gene and directed at cognate sequences located within a relatively small distance (which in the case of saMB1_1 and saMB1_2 is ∼450 bp) failed to induce RNAa, likely due to spatial interference of a transcribing complex initiating at one site and arrival or assembly of another complex at another site within close proximity. Our hypothesis on steric hindrance is supported by the lack of AGO2 and RNAPII recruitment when both duplexes targeting nearby sites are used in combination. Consistently, saRNAs targeting genes located on distinct chromosomes recruited key RNAa components and were able to induce RNAa when used concurrently. This may have important implications for simultaneous RNAa-based enhancement of *MBNL* paralogs for potentially additive and/or synergistic therapeutic effect.

RNAa can be harnessed to identify gene expression regulators. We demonstrated the contribution of specific transcription activators, MEIS1 and MEIS2, to saMB1_1-mediated *MBNL1* upregulation. MEIS proteins belong to the TALE (Three-Amino-acid Loop Extension) family of homeobox genes and can directly or cooperatively bind DNA with various TFs (e.g. HOX, PBX, and PARP1), hence their role in effective transcriptional activation, regulation of chromatin dynamics and gene expression in multiple processes [68]. It is plausible that MEIS proteins associate with RITA complex in *MBNL1* transcriptional upregulation via saMB1_1. To uncover novel *MBNL1* expression regulators, further studies should investigate protein components participating in *MBNL1* RNAa via identified saRNAs, e.g. via pull-down of their chemically modified derivatives complexed with interacting partners, followed by proteomic analyses.

Our comprehensive study involving experiments with chemical modifications of saRNA strands as well as *MBNL1* promoter luciferase assays indicated that the identified saMB1_1 and saMB1_2 saRNAs target DNA via an on-site mechanism involving the AS strand. While inhibition of the AS strand completely blocked *MBNL1* induction, promotion of the S strand selection by AGO2 failed to induce RNAa. Accordingly, luciferase assays suggested a direct physical interaction of the AS strand of saMB1_2 with the cognate target site within the *MBNL1* promoter. Although several lines of evidence suggest that saRNA may also bind natural antisense transcripts overlapping promoters to increase target gene expression [50, 52], we ruled out lncRNA *MBNL1-AS1* requirement for *MBNL1* RNAa. Consistently with published works [59], our data allow to conclude that the AS strand serves as the guide/leading strand and targets complementary DNA sequence within the *MBNL1* promoter, whereas the S strand has minimal impact on RNAa. Intriguingly, our study revealed that the lead saRNAs triggered consistent downregulation of *MBNL1-AS1* overlapping *MBNL1* P2, but only when their S strands were active. One likely scenario is that the S strand of saRNA directly interacts with antisense transcript through RNA-RNA base pairing, potentially leading to its destabilization, higher transcript turnover or degradation via mechanism other than RNAi, as we ruled out the role of AGO2 in this process. Alternatively, saRNA could recruit chromatin-modifying complexes to the promoter region of the target gene, indirectly affecting transcription of neighboring antisense transcripts by altering the accessibility of their promoters or enhancer regions. The former scenario is supported by higher *MBNL1-AS1* turnover observed in our experiments with actinomycin D-mediated transcription inhibition. The latter possibility, however, is also reinforced by reduced levels of nascent lncRNA *MBNL1-AS1*. It is plausible that both mechanisms participate independently to bring about *MBNL1-AS1* reduction upon *MBNL1* RNAa. Notably, saRNAs’ effects on antisense transcripts can vary depending on the specific genomic context and regulatory mechanisms involved. In support, Schwartz *et al.* [52] proposed that progesterone receptor (PR) activation is attributable to an association between saRNA and the antisense transcript AT-2 overlapping the PR promoter. This view was challenged by another study showing AT-2 dispensability for PR RNAa [58] and our own results also excluded the requirement for *MBNL1-AS1* in *MBNL1* RNAa. Further, silencing of *MBNL1-AS1* affected neither the basal expression level of *MBNL1* nor saRNA-mediated *MBNL1* upregulation. This demonstrates that first, this lncRNA is not a negative regulator whose removal can relieve negative pressure on its sense counterpart and second, it is mechanistically dispensable for *MBNL1* RNAa. Moreover, we demonstrated that saRNA treatment does not induce the production of promoter-associated nascent sense transcripts originating from the saMB1_2 target region that could serve as docking sites for the AS strand of saRNA at *MBNL1* promoter. Altogether, our results negate the contribution of the RNA:RNA model in RNA activation of *MBNL1* gene by the two identified saRNAs.

An important caveat to be considered with all RNA-based antisense approaches is potential off-targeting, mediated via binding to unintended RNA targets even in the case of partial sequence complementarity. In our work, an unexpected effect involved downregulation of lncRNA *MBNL1-AS1* overlapping *MBNL1* P2, mediated clearly via S strand of saRNA duplex. Even though saRNAs did not adversely affect cell viability, cytotoxicity or apoptosis for up to 5 days post-transfection, we cannot exclude the long-term effect of persistent *MBNL1-AS1* downregulation upon *MBNL1* RNAa. Importantly, this unanticipated effect could be eliminated completely in the case of saMB1_1, and partially in the case of saMB1_2, via simple chemical modification that inhibited the S strand activity: 5′-end biotin conjugation. Moreover, introducing only one 3′-most nucleotide mismatch within the S strand was sufficient to fully abolish *MBNL1-AS1* downregulation without affecting the overall RNAa efficiency, but only in the case of saMB1_1 and not saMB1_2. Together with data showing that saRNA seed region mutations were unable to prevent *MBNL1-AS1* downregulation, all these results support two conclusions (i) that the off-target effect mediated via the S strand does not rely on a perfect sequence complementarity, and (ii) that careful manipulation of the S strand activity can completely or partially abrogate unwanted side effects without compromising RNAa efficiency.

All cell models used in this work were susceptible to *MBNL1* induction via RNAa, albeit with varying efficiency. These differences may stem from local epigenetic status (e.g. DNA methylation) and chromatin accessibility across diverse human cell lines as well as efficiency of saRNA uptake by e.g. fibroblasts vs. muscle cells and inherent differences in metabolic properties of distinct cell lines. Indeed, delivery of dsRNA to patients’ diseased cells and tissues has been a major challenge in RNA-based therapy [75]. Intriguingly, siRNA delivery *in vivo* can be significantly improved through lipid conjugation e.g. cholesterol [76], while lipid- and polymer-based nanoparticles are well known to prolong their circulation time, stability, and bioavailability *in vivo* [77]. Likewise, recent work within the RNAa field has shown an effective intravenous delivery into mice of *HNF4A*-targeted saRNA linked with dendrimer nanoparticles [78] or conjugated aptamers [79]. Moreover, advanced saRNA-based therapy in clinical trials in patients with advanced hepatocellular carcinoma consists of *CEBPA-*targeted saRNA linked to liposomal nanoparticles (SMARTICLES™) delivered via intravenous infusion [80]. Further, recently launched phase I clinical trial will examine lipid-conjugated (LiCO™) *p21*-targeted saRNA in patients with non-muscle-invasive bladder cancer, delivered through intravesical instillation (clinicaltrials.gov identifier NCT06351904) [81]. In this novel saRNA-based drug, RAG-01, *p21*-targeted saRNA is conjugated with a lipid-based delivery system via a benzimidazole linker. It would be interesting to test whether lipid-based modifications could positively affect saMB1_1 and saMB1_2 uptake and response in cellular DM1 models.

RNAa strategy presented in this work targets solely *MBNL1* with potential implications for normalized skeletal muscle function, which warrants further studies in DM1 myoblasts. However, the disease affects multiple other tissues and cell types that express varying levels and intracellular locations of distinct *MBNL* paralogs. Brain and heart, apart for muscle, are major organs affected by DM1. Considering the feasibility of combined RNAa of distinct genes, simultaneous targeting of *MBNL* paralogs expressed in distinct organs (e.g. *MBNL1* and the major paralog in the brain, *MBNL2*) poses an attractive option to significantly increase therapeutic benefits through e.g. synergistic or additive effect. However, poor delivery and uptake to differentiated cells constitute serious limitations. For example, none of the currently available disease modifying treatments have the potential to cross the blood brain barrier, so remediating compromised CNS functions remains a challenge. Caveats aside, RNA-based therapeutics, unlike gene editing approaches, are not permanent and temporarily but often long-lastingly mitigate disease severity. Although this necessitates repeated rounds of treatment, it offers the benefit of dose adjustment and withdrawal whenever more advanced therapeutic options become available. Currently, however, evaluation of the pharmacokinetics, biodistribution, and the mechanism of tissue penetration by saRNAs in DM1 is limited by lack of suitable mouse models due to poor promoter conservation between human and mouse.

In conclusion, this is the first report of a directed, site-specific manipulation of the endogenous *MBNL1* promoter to enhance *MBNL1* transcription. Our data distinctly revealed therapeutic potential of RNAa in DM1, reflected by increased MBNL1 protein content and correction of AltS of MBNL1 target pre-mRNAs. This directed approach, when adapted into RNAa-based therapy, may offer several advantages including high specificity, reversibility, dose-control, and potential for tissue-specific delivery when coupled with distinct carriers. Future studies should address chemical modifications for improved delivery, minimized off-target effect, as well as putative synergistic and/or additive effect of saRNAs targeted simultaneously to other *MBNL* paralogs for enhanced splicing correction. Finally, saRNA-based approach not only expands possible points of therapeutic interventions for future drug developments in DM but may also serve as a novel molecular toolbox to interrogate protein factors involved in *MBNL1* transcription. As such, our findings offer brand new perspectives on *MBNL1* gene expression regulation and may likely foster future research on RNAa in the context of targeted DM1 therapy.

## Supplementary Material

gkaf756_Supplemental_File

## Data Availability

All data generated or analyzed during this study are included in this published article and its supplementary information files.
