# Supplementary material for: Promoter-targeted small RNA duplexes increase MBNL1 transcription and mitigate myotonic dystrophy-associated spliceopathy
Source: Nucleic Acids Res. 2025 Aug 20;53(15):gkaf756. doi: 10.1093/nar/gkaf756 (PMC12364543; doi:10.1093/nar/gkaf756)
Supplement: gkaf756_Supplemental_File [file gkaf756_supplemental_file.pdf]

**Supplementary Material For:**

# **Promoter-targeted small RNA duplexes increase *MBNL1* transcription and mitigate myotonic dystrophy-associated spliceopathy.**

Nikola Musiała-Kierklo[1,2], Patrycja Plewka[1], Adam Jasiok[1,3] and Ewa Stępniak-Konieczna[1,*]

[1]Laboratory of RNA Biology, Department of Biochemistry and Biotechnology, Poznan University of Life Sciences, Dojazd 11, 60-632 Poznan, Poland

[2]Doctoral School of Natural Sciences, Adam Mickiewicz University, Uniwersytetu Poznanskiego 2, 61-614 Poznan, Poland

[3]Poznan University of Life Sciences Doctoral School, Collegium Maximum, Wojska Polskiego 28, 60-637 Poznan, Poland

* To whom correspondence should be addressed: ewa.stepniak-konieczna@up.poznan.pl

CONTENT:

1. LIST OF SUPPLEMENTARY FIGURES S1-S17 AND TABLES S1-S6
2. SUPPLEMENTARY FIGURES S1-S17 AND CORRESPONDING FIGURE LEGENDS
3. SUPPLEMENTARY TABLES S1-S6 AND CORRESPONDING LEGENDS
4. SUPPLEMENTARY REFERENCES

## 1. LIST OF SUPPLEMENTARY FIGURES S1-S17 AND TABLES S1-S6

**Supplementary Figure S1.** The activity of *MBNL1* promoters differs in DM1 cells.

**Supplementary Figure S2.** Screening of saRNAs directed at *MBNL1* promoters identifies two top scoring duplexes, saMB1_1 and saMB1_2, specifically enhancing P2-derived *MBNL1* mRNA levels in DM1 fibroblasts.

**Supplementary Figure S3.** Dose-response, kinetics, specificity and combined effect of saRNA duplexes in DM1 fibroblasts.

**Supplementary Figure S4.** saRNA duplexes upregulate *MBNL1* RNA in distinct cell models.

**Supplementary Figure S5.** saRNA duplexes stimulate the recruitment of specific transcription factors to induce *MBNL1* RNAa.

**Supplementary Figure S6.** RNA interference-mediated knock-down of AGO2 prevents RNA activation of *MBNL1*.

**Supplementary Figure S7.** Directional RT-PCR reveals absence of cryptic short sense RNA and downregulation of antisense RNA at the saMB1_2 target site within *MBNL1* promoter P2.

**Supplementary Figure S8.** saRNA duplexes targeted to *MBNL1* promoter P2 induce downregulation of lncRNA *MBNL1-AS1*.

**Supplementary Figure S9.** Reduced antisense transcription and higher transcript turnover contribute to the reduction of lncRNA *MBNL1-AS1* upon *MBNL1*-directed saRNAs.

**Supplementary Figure S10.** The sense strand of *MBNL1*-directed saRNA mediates lncRNA *MBNL1-AS1* downregulation.

**Supplementary Figure S11.** Unadjusted and uncropped western blot images corresponding to Figure 6.

**Supplementary Figure S12.** Timecourse analyses show correlation of MBNL1 protein induction upon saRNA treatment with MBNL1-dependent e1 exclusion from *MBNL1* pre-mRNA.

**Supplementary Figure S13.** Colocalization of MBNL1 and $CUG^{exp}$ RNA foci in DM1 fibroblasts is not affected by saRNA-mediated *MBNL1* upregulation.

**Supplementary Figure S14.** Unadjusted and uncropped RT-PCR gel images of alternative splicing events in DM1 fibroblasts shown in Figure 8A.

**Supplementary Figure S15.** Unadjusted and uncropped RT-PCR gel images of alternative splicing events in DM1 myoblasts shown in Figure 8B.

**Supplementary Figure S16.** Sequence-scrambled or seed region mutant versions of saMB1_1 and saMB1_2 do not affect *MBNL1*-regulated alternative splicing.

**Supplementary Figure S17.** saRNA duplexes do not affect cell viability, toxicity and apoptosis.

**Supplementary Table S1.** Sequences of saRNAs and control RNA duplexes used in this study.

**Supplementary Table S2.** Sequences of chemically modified and mismatch-containing saRNA duplexes for strand inhibition or promotion.

**Supplementary Table S3.** siRNA and GapmeR sequences.

**Supplementary Table S4.** Expression primers for RT-qPCR.

**Supplementary Table S5.** Alternative splicing primers.

**Supplementary Table S6.** CUT&RUN primers for qPCR.

## 2. SUPPLEMENTARY FIGURES S1-S17 AND CORRESPONDING FIGURE LEGENDS

### Supplementary Figure S1.

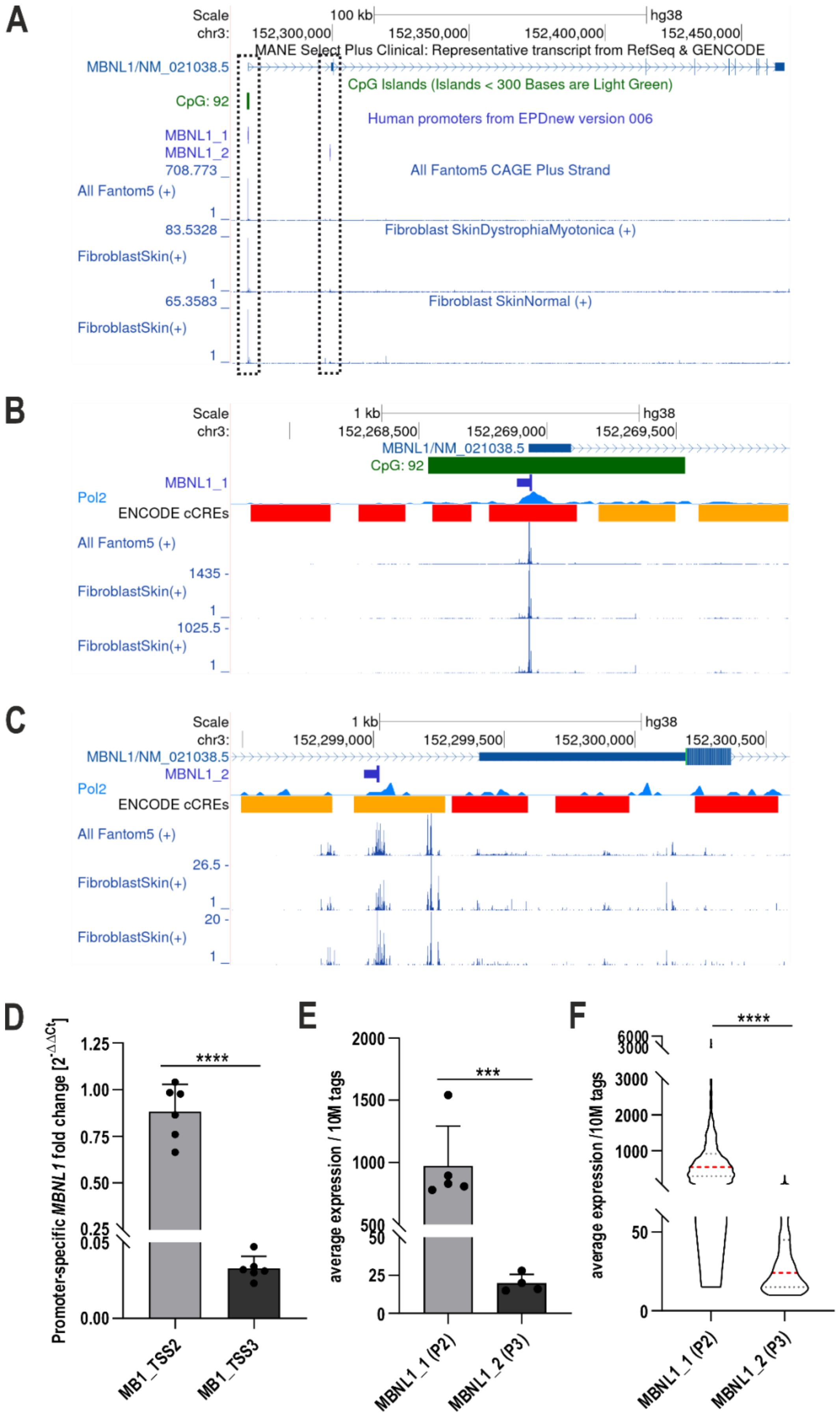

**Supplementary Figure S1. The activity of *MBNL1* promoters differs in DM1 cells. (A-C)** UCSC Genome Browser view of *MBNL1* gene with track display including EPD v006 promoters *MBNL1_1* (P2) and *MBNL1_2* (P3), CpG islands, ENCODE candidate Cis-Regulatory Elements (cCREs), ChIP-seq for RNA polymerase II in CD4 cells (1) and FANTOM5 mapped transcription start sites (TSS) in normal as well as myotonic dystrophy skin fibroblasts. cCREs color code in (B-C) marks promoter-like signatures (red) and proximal enhancer-like signature (orange). Blow-up panels of *MBNL1* P2 and P3-associated TSS2 and TSS3 regions in **(B)** and **(C)**, respectively, correspond to regions marked in (A) by dotted line rectangles. **(D)** Relative *MBNL1* transcript levels derived from TSS2 and TSS3 assessed via RT-qPCR-based analyses in GM04033 DM1 fibroblasts. Data are presented as mean ± SEM of n=6 samples. **(E)** Meta-analysis of promoter-specific *MBNL1* expression in DM1 patients' skin-derived fibroblasts based on data deposited in Eukaryotic Promoter Database (EPD). Analyzed data represent either whole-cell lysates or nuclear fractions from 3 distinct DM1 donors (n=5 samples available for P2 and n=4 samples available for P3). **(F)** Meta-analysis of the average expression level of P2- and P3-derived *MBNL1,* in all samples in which these promoters are active (n=1051 for P2; n=371 for P3), based on data deposited in Eukaryotic Promoter Database (EPD). Average expression in (E) and (F) represents the number of tags in a 100-bp region centered on the EPD-annotated respective TSS and normalized to 10M total tags. Red horizontal lines in (F) represent average expression: 695.655 tags per 10M for P2 and 35.5714 tags per 10M for P3, grey dashed lines in (F) indicate quartiles.

**Supplementary Figure S2.**

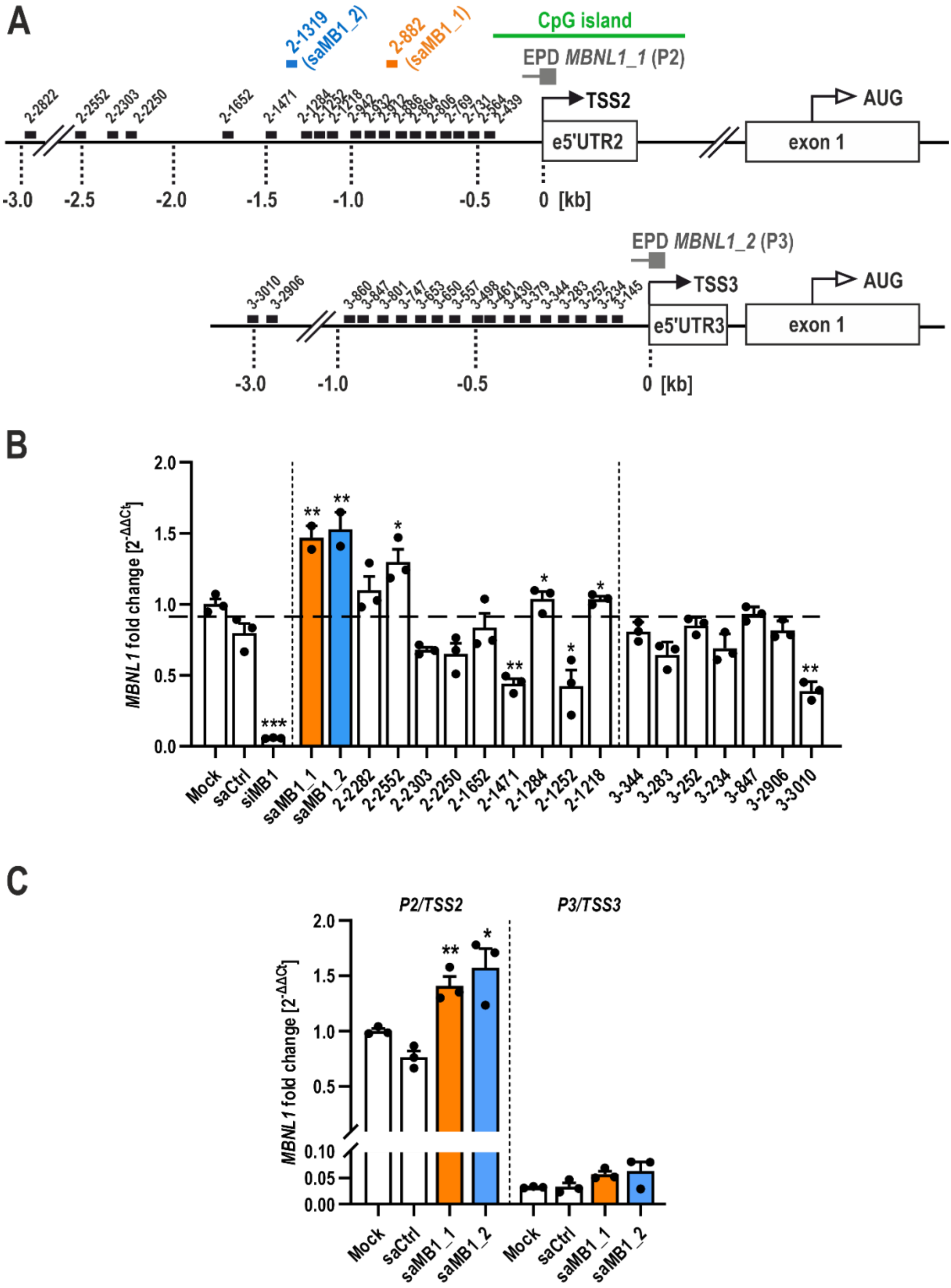

**Supplementary Figure S2. Screening of saRNAs directed at *MBNL1* promoters identifies two top scoring duplexes, saMB1_1 and saMB1_2, specifically enhancing P2-derived *MBNL1* mRNA levels in DM1 fibroblasts. (A)** Schematic representation, not to scale, of *MBNL1* genomic regions (according to NCBI RefSeq Select and MANE subset: a single representative transcript) spanning P2 (upper panel) and P3 (lower panel). Indicated features include: EPD-annotated promoters (*MBNL1_1* (P2) and *MBNL1_2* (P3), respectively),

transcription start sites (TSS2 and 3, respectively), 5′ UTR exons (e5′UTR2 and 3, respectively) and the first coding exon (exon 1) with translation start site (AUG). Target sites of saRNAs are indicated. **(B)** Representative results of saRNAs screening (selected duplexes), based on RT-qPCR analyses of *MBNL1* mRNA levels in GM04033 cells transfected with 75 nM indicated saRNAs targeting P2 and P3, for 120 h. siMB1 refers to samples with RNAi-mediated knock-down of *MBNL1*. **(C)** RT-qPCR expression analysis of P2- and P3-derived *MBNL1* transcripts in GM04033 cells transfected with 75 nM indicated saRNA for 120 h.

**Supplementary Figure S3.**

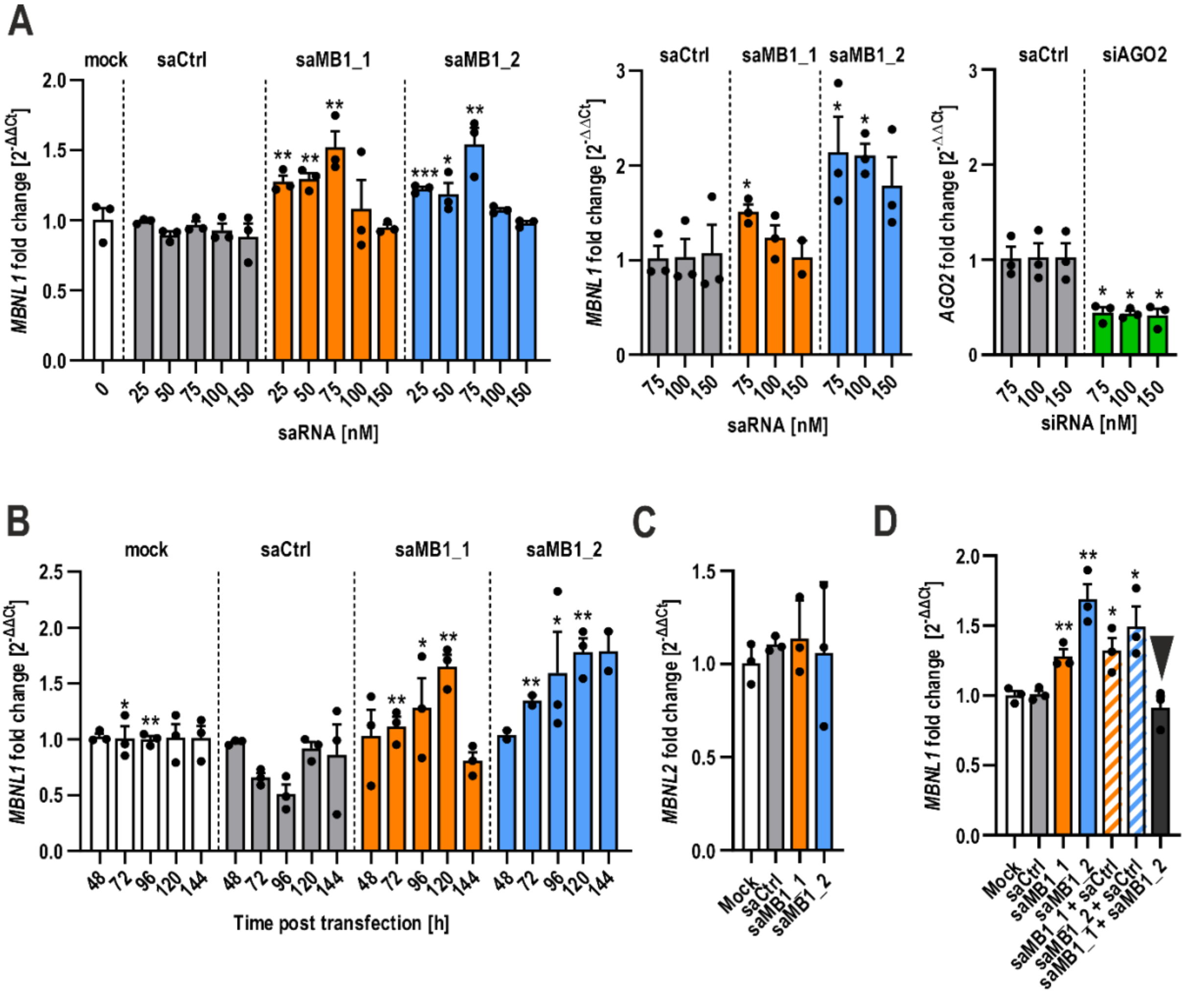


**Supplementary Figure S3. Dose-response, kinetics, specificity and combined effect of saRNA duplexes in DM1 fibroblasts. (A)** RT-qPCR analyses of indicated transcripts in GM04033 cells transfected for 120 h with indicated amounts of dsRNA. The saRNA dose-response study at a broad range of concentrations (25-150 nM; left panel) as well as a comparative dose-response study of saRNAs (middle panel) and a well characterized siRNA against *AGO2* transcripts (right panel) at a defined range of high doses (75-150 nM) were performed. **(B)** Timecourse RT-qPCR analysis of *MBNL1*, showing RNAa kinetics at 75 nM saRNA across indicated timepoints post-transfection. **(C-D)** RT-qPCR analysis of *MBNL2* (C) or *MBNL1* (D) expression in GM04033 cells transfected with 75 nM indicated saRNA for 120 h. In (D) 75 nM saMB1_1 and 75 nM saMB1_2 were transfected sequentially such that 24 h treatment with one saRNA was followed by transfection of the second saRNA for additional 96 h (black inverted arrowhead). The order of transfection is as labelled. Sequential transfection of either saMB1_1 or saMB1-2 followed by saCtrl (striped bars) was used as a control.

**Supplementary Figure S4.**

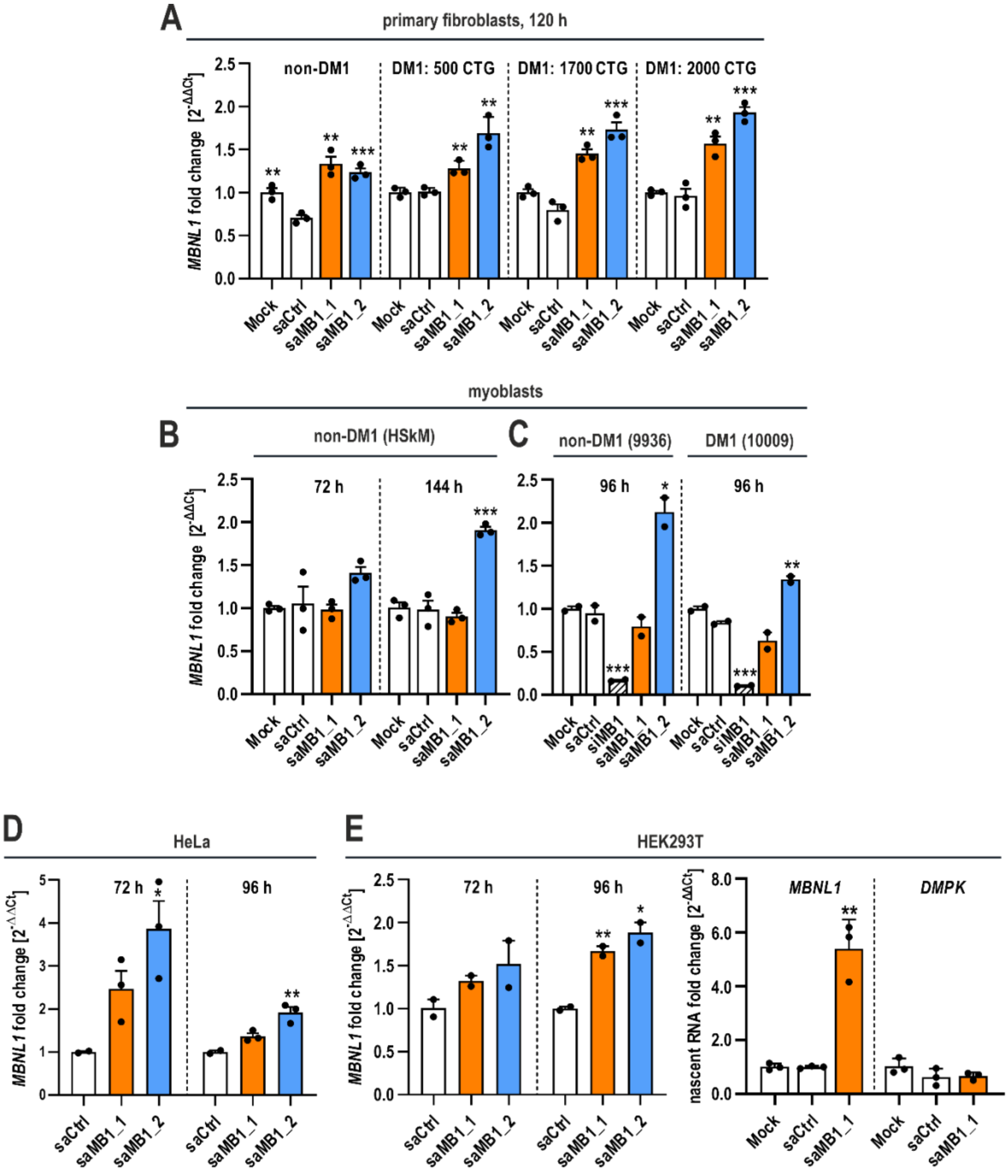


**Supplementary Figure S4. saRNA duplexes upregulate *MBNL1* RNA in distinct cell models. (A-D)** Representative results of RT-qPCR analyses of *MBNL1* expression upon transfection with indicated top scoring saMB1_1 and saMB1_2 in distinct cell models: **(A)** primary fibroblasts GM07492 (non-DM1) or GM03987, GM03132 and GM03989 expressing *DMPK* gene with ~500, ~1700 and ~2000 CTG repeats, respectively, **(B)** Primary Normal Human Skeletal Myoblasts (HSkM), **(C)** myoblasts 9936 (non-DM1; left) and 10009 (DM1; right), **(D)** HeLa and **(E, left panel)** HEK293T. The following experimental conditions were used: 75 nM saRNA for 120 h (A), 75 nM saRNA for 72 h and 144 h (B), 75 nM saRNA for 96 h (C), 75 nM saRNA for 72 h and 96 h (D and E, left panel). In (B), HSkM cells were first transfected with 75 nM saRNA for 72 h upon which 50% cells were collected (first analysis – 72 h, left) and the remaining 50% cells were seeded, transfected again with 75 nM saRNA and harvested 72 h post second transfection (second analysis – 144 h, right). In (D) and (E, left panel), Hela and

HEK293T cells, respectively, were transfected and 50% were collected 72 h later for the first analysis (72 h) and the remaining 50% cells were seeded and allowed to grow for additional 24 h, upon which second analysis was performed (96 h). **(E, right panel)** RT-qPCR results of nascent RNA capture of *MBNL1* and *DMPK* (control) in HEK293T cells transfected with 75 nM saMB1_1 for 48 h, followed by 24 h 5-EU pulse. Results were normalized to *GAPDH* and presented as mean ± SEM of triplicate (A, B, D and right panel in E) or duplicate samples (C and left panel in E).

**Supplementary Figure S5.**

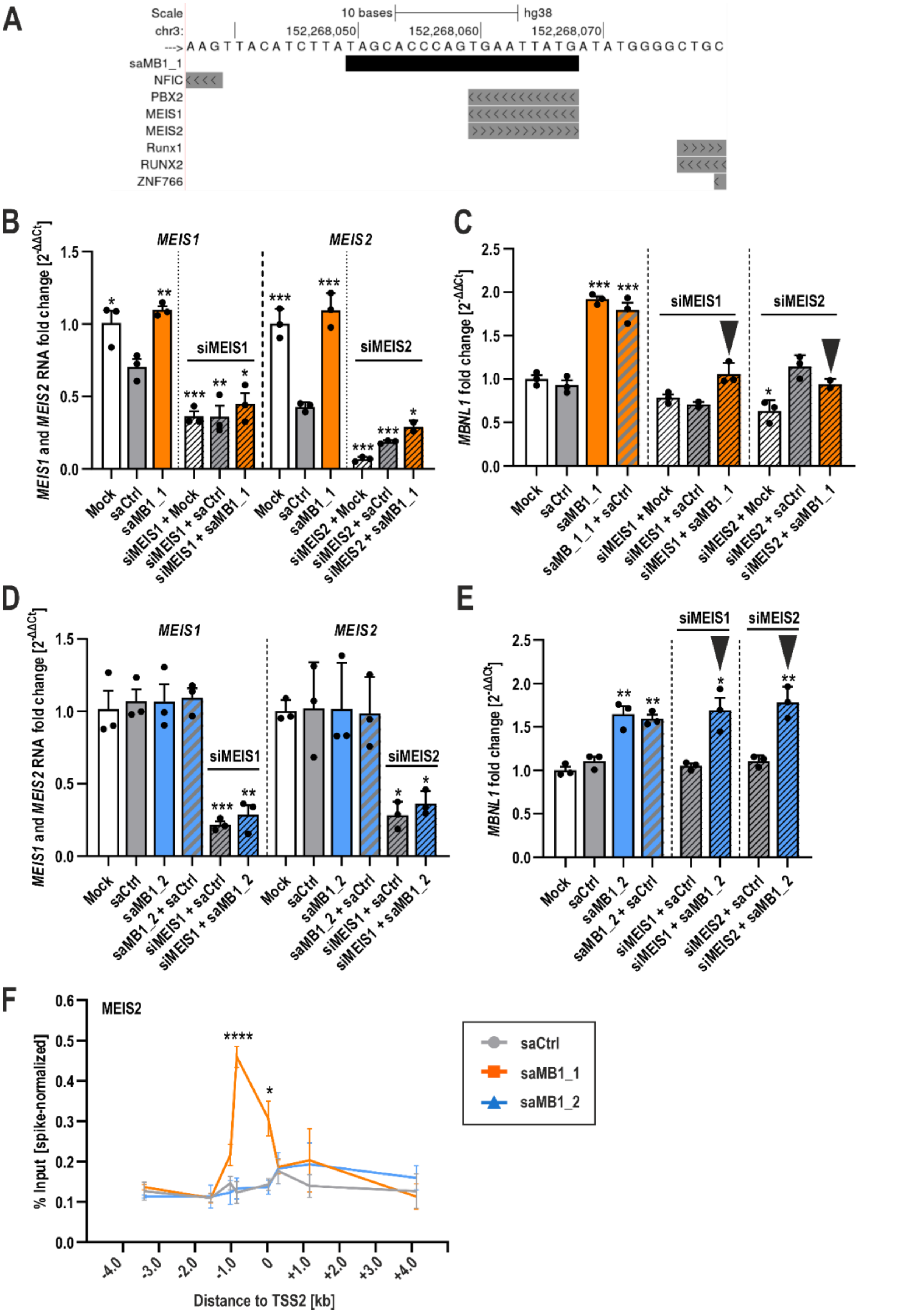


**Supplementary Figure S5. saRNA duplexes stimulate the recruitment of specific transcription factors to induce *MBNL1* RNAa. (A)** UCSC Genome Browser track image

showing saMB1_1 target genomic sequence within *MBNL1* promoter 2 region. Predicted transcription factors binding sites overlapping saMB1_1 target site are indicated (PBX2, MEIS1, MEIS2). **(B-C)** RT-qPCR assay of *MEIS1* and *MEIS2* (B) or *MBNL1* (C) in GM04033 DM1 fibroblasts upon knock-down of *MEIS1* (siMEIS1) or *MEIS2* (siMEIS2) and subsequent RNAa of *MBNL1* via saMB1_1. Cells were transfected with lipofectamine (mock) or 75 nM indicated dsRNA (saCtrl, saMB1_1, siMEIS1 or siMEIS2). After 24 h, siMEIS1 and siMEIS2-treated samples were subjected to a second transfection round with lipofectamine alone (mock) or 75 nM indicated dsRNA (saCtrl or saMB1_1) for additional 96 h. In (C), additional control included cells transfected sequentially with 75 nM saMB1_1 and an equal amount of saCtrl added 24 h later, for a total of 120 h (orange / grey striped bar). Black inverted arrowheads in (C) indicate samples in which knock-down of MEIS1 or MEIS2 inhibited *MBNL1* induction via saMB1_1. **(D-E)** RT-qPCR assay of *MEIS1* and *MEIS2* (D) or *MBNL1* (E) in GM04033 cells upon knock-down of *MEIS1* (siMEIS1) or *MEIS2* (siMEIS2) and subsequent RNAa of *MBNL1* via saMB1_2. Experimental settings in (D) and (E) are analogous to those shown in (B) and (C), respectively, with the exception that saMB1_2 was used instead of saMB1_1. Black inverted arrowheads in (E) indicate samples in which knock-down of MEIS1 or MEIS2 failed to inhibit *MBNL1* induction via saMB1_2. **(F)** qPCR results of CUT&RUN analyses in GM04033 cells transfected with 75 nM indicated saRNA for 120 h, demonstrating binding enrichment of MEIS2 to indicated genomic regions (ranging from −3.5 kb to +4.2 kb relative to *MBNL1* TSS2), following transfection of saMB1_1 (orange line), but not saMB1_2 (blue line) or saCtrl (grey line). Results were normalized using Spike-In DNA and are represented as % of input.

**Supplementary Figure S6.**

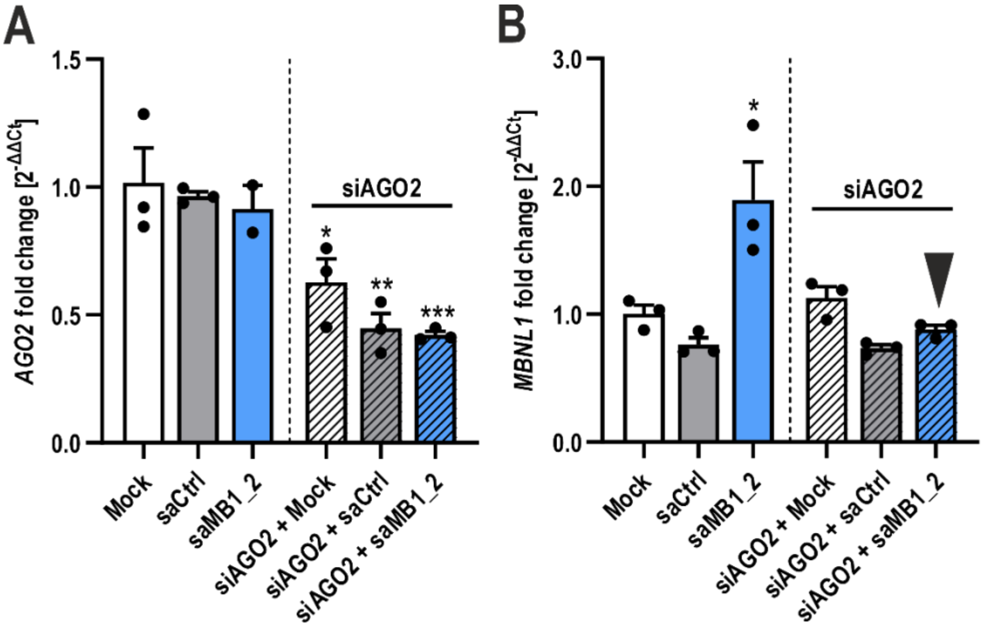


**Supplementary Figure S6. RNA interference-mediated knock-down of AGO2 prevents RNA activation of *MBNL1*.** RT-qPCR analyses of *AGO2* **(A)** and *MBNL1* **(B)** expression in GM04033 DM1 fibroblasts transfected for 24 h with lipofectamine alone (mock) or 75 nM indicated dsRNA (saCtrl, saMB1_2 or siAGO2). After 24 h, only siAGO2-transfected samples (right parts of the graphs) were subjected to a second round of transfection with lipofectamine alone (mock) or 75 nM saCtrl or saMB1_2, for additional 96 h. After a total of 120 h, all samples were harvested for analyses. Black inverted arrowhead in **(B)** indicates the samples in which knock-down of AGO2 inhibited *MBNL1* induction by saMB1_2.

**Supplementary Figure S7.**

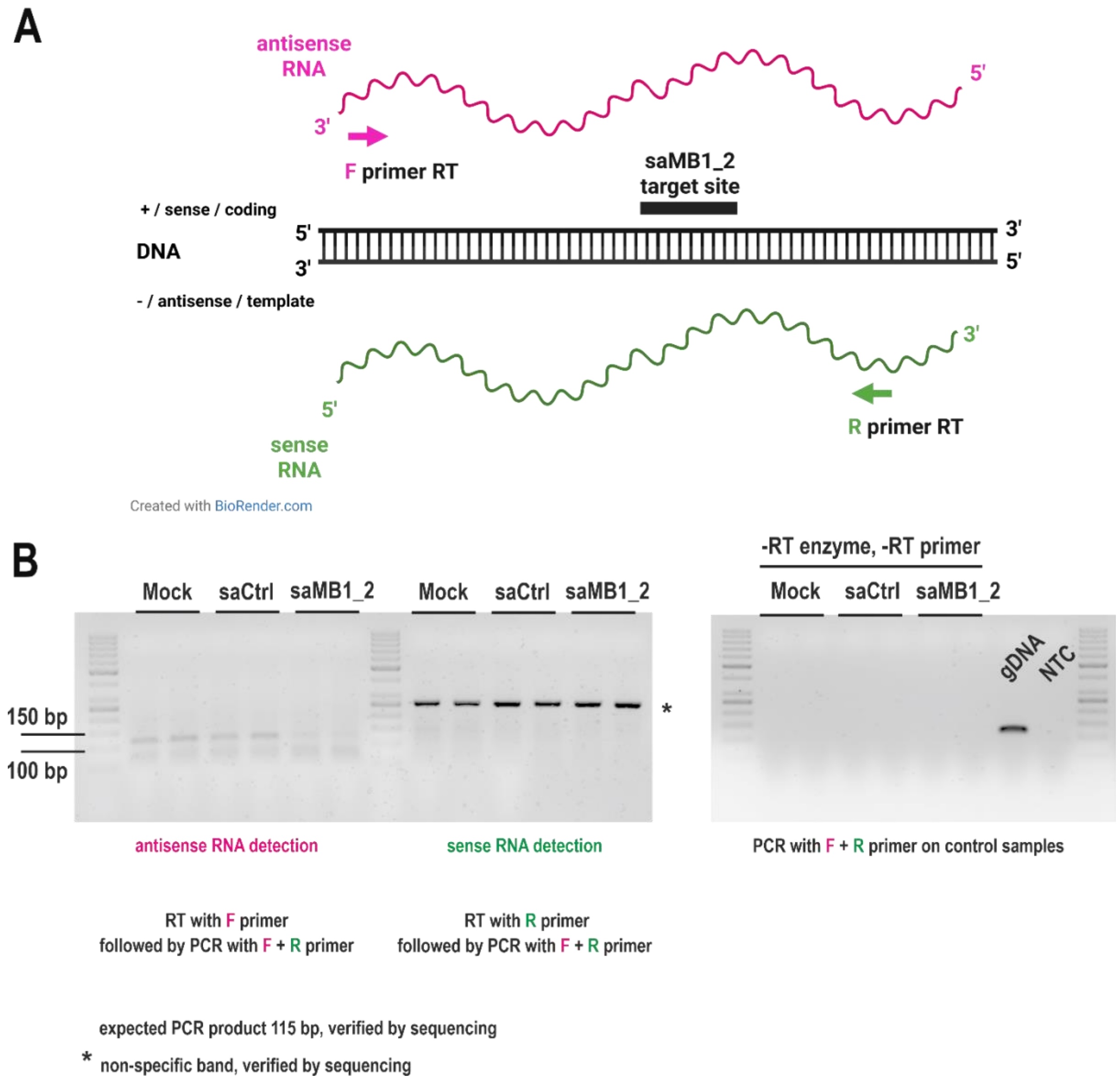


**Supplementary Figure S7. Directional RT-PCR reveals absence of cryptic short sense RNA and downregulation of antisense RNA at the saMB1_2 target site within *MBNL1* promoter P2. (A)** Schematic illustration of the directional RT-PCR assay used to detect and verify the directionality of the transcripts originating from the saMB1_2 target genomic region. Note that F (forward primer) and R (reverse primer) flank the saMB1_2 binding site. created in BioRender. Stepniak-Konieczna, E. (2025) https://BioRender.com/noo48m8. **(B)** Directional RT-PCR analysis of antisense and sense transcripts derived from the genomic region encompassing saMB1_2 binding site in HeLa cells treated for 72 h with lipofectamine alone (mock) or 75 nM indicated saRNAs (left panel). To exclude contamination by amplification of residual genomic DNA, control samples containing all reaction components except for the reverse transcriptase (RT enzyme) and RT primers were included in PCR reactions (right panel). gDNA- genomic DNA control; NTC- no-template control.

**Supplementary Figure S8.**

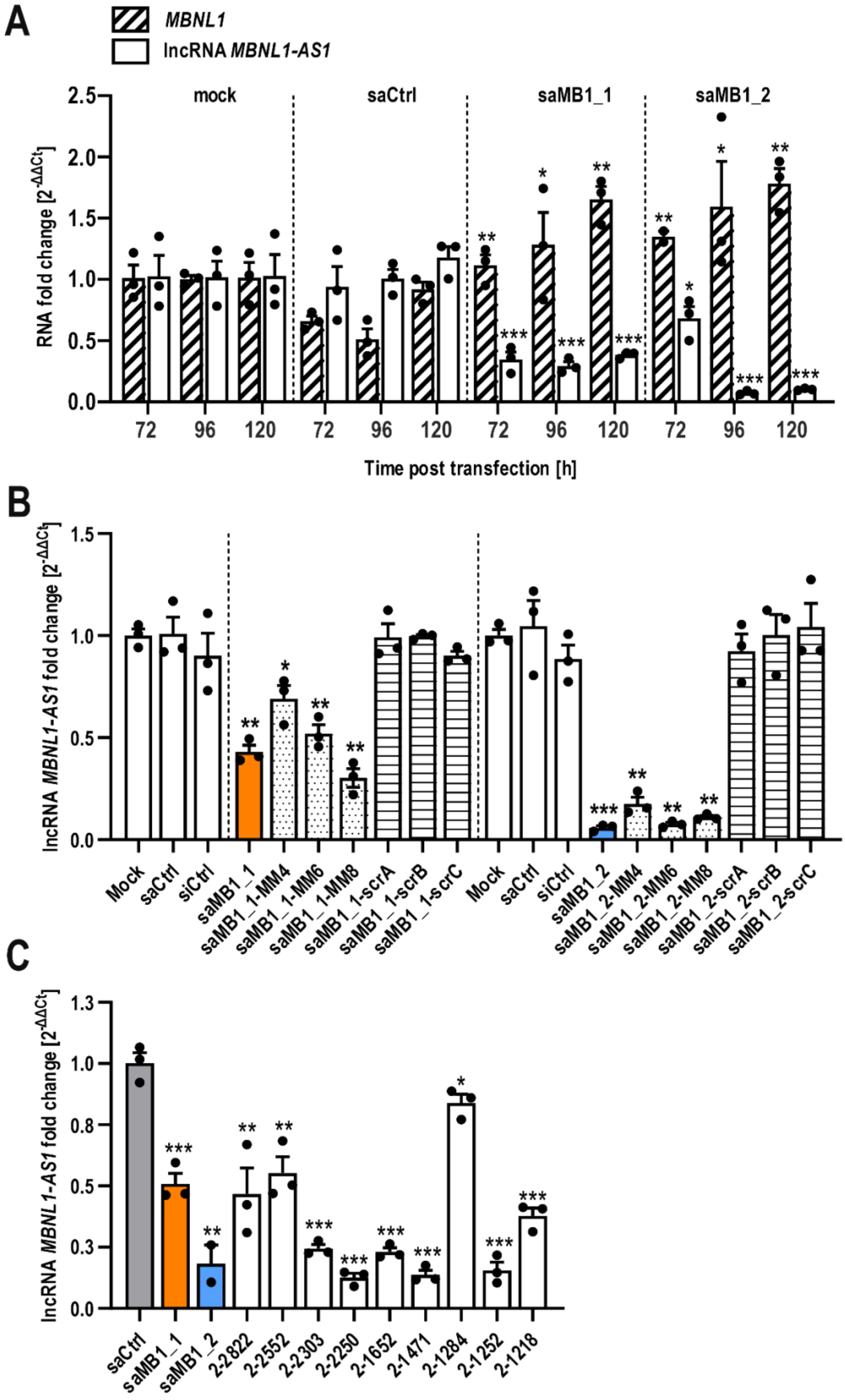


**Supplementary Figure S8. saRNA duplexes targeted to *MBNL1* promoter P2 induce downregulation of lncRNA *MBNL1-AS1*. (A)** RT-qPCR-based timecourse analyses (72 – 120

h) of *MBNL1* and *MBNL1-AS1* levels in GM04033 DM1 fibroblasts transfected with 75 nM indicated saRNA duplexes targeted to *MBNL1* gene promoter 2. **(B)** RT-qPCR analysis of lncRNA *MBNL1-AS1* level in GM04033 cells transfected for 120 h with 75 nM of control RNA duplexes (saCtrl or siCtrl), lead saRNAs (saMB1_1 and saMB1_2) or their modified versions carrying a single nucleotide mismatch within the seed region at position 4, 6 or 8 (MM4, 6, 8), or their scrambled sequence versions (scrA-C). **(C)** RT-qPCR analysis of lncRNA *MBNL1-AS1* level in GM04033 cells transfected with 75 nM indicated saRNA for 120 h.

**Supplementary Figure S9.**

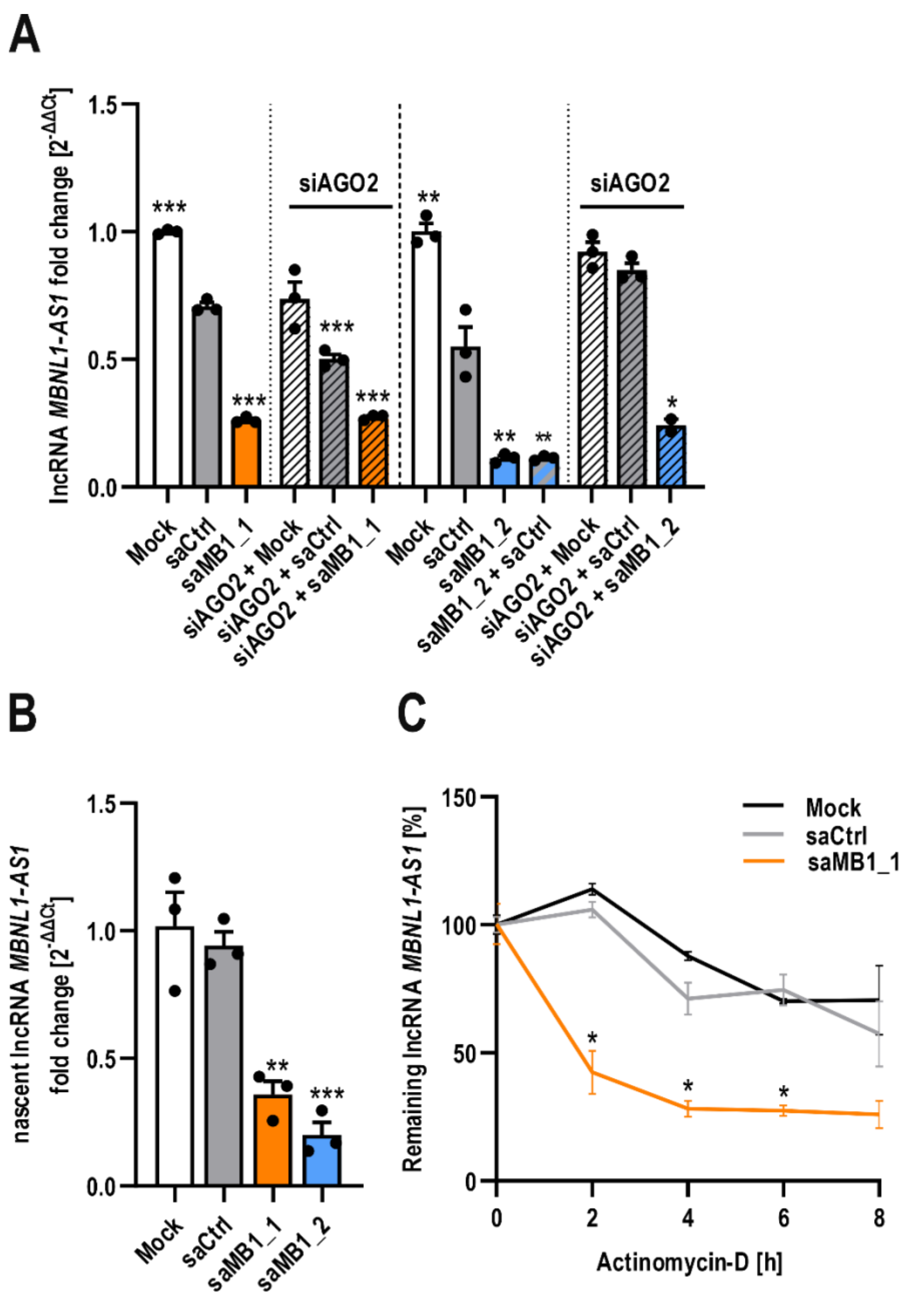


**Supplementary Figure S9. Reduced antisense transcription and higher transcript turnover contribute to the reduction of lncRNA *MBNL1-AS1* upon *MBNL1*-directed saRNAs. (A)** RT-qPCR of *MBNL1-AS1* in GM04033 DM1 fibroblasts transfected with lipofectamine (mock) or 75 nM indicated saRNA delivered solo (saCtrl, saMB1_1 or saMB1_2), or sequentially such that the 24 h transfection of 75 nM siAGO2 was followed by a second transfection of either lipofectamine alone (mock), 75 nM saCtrl or one of the two lead saRNA duplexes (saMB1_1 or saMB1_2) for additional 96 h. In the case of saMB1_2-transfected samples, additional control also included a second transfection with 75 nM saCtrl (blue / grey striped bar). All analyses were performed after a total of 120 h. **(B)** RT-qPCR-based analysis of nascent RNA capture of *MBNL1-AS1* assayed in GM04033 cells transfected with lipofectamine alone (mock) or 75 nM indicated saRNA for 72 h, followed by 24 h EU pulse and capture using Click-iT chemistry. **(C)** RT-qPCR-based timecourse analysis (0-8 h) of the remaining lncRNA *MBNL1-AS1* upon actinomycin D (Act-D) treatment of GM04033 cells transfected with 75 nM saMB1_1 (orange) or saCtrl (grey) for 120 h. Results obtained in lipofectamine-treated cells

(mock, black) are shown for reference. Act-D was added 120 h post saRNA transfection (timepoint 0 of the timecourse analysis). Verification of *MBNL1* upregulation 120 h post saRNA treatment is shown in Figure 2B, right panel. Data in (C) are presented as mean ± SEM of duplicate samples normalized to *GAPDH*.

**Supplementary Figure S10.**

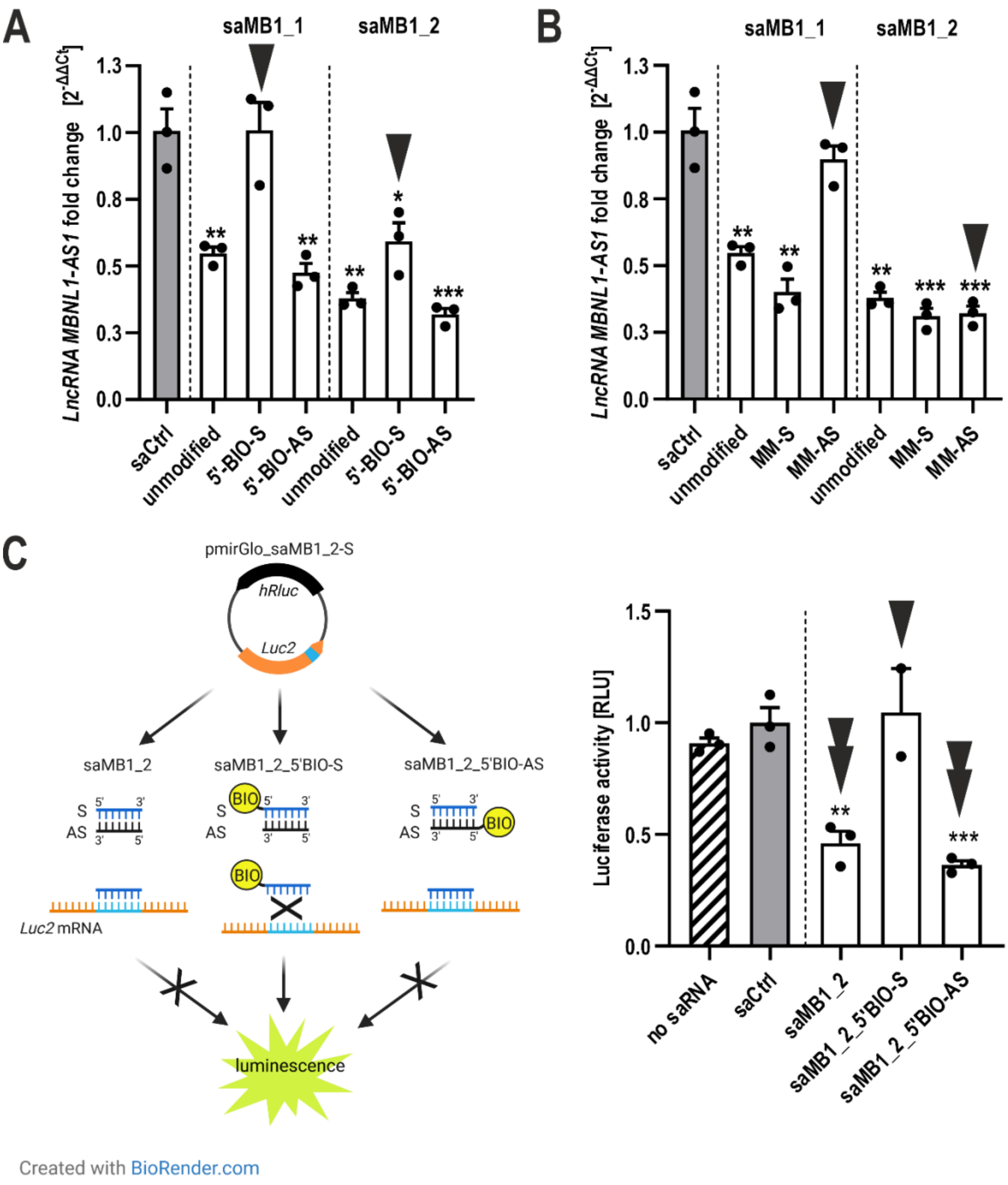


**Supplementary Figure S10. The sense strand of *MBNL1*-directed saRNA mediates lncRNA *MBNL1-AS1* downregulation. (A-B)** RT-qPCR analyses of lncRNA *MBNL1-AS1* in samples shown in Figure 4D-E, demonstrating correlation of *MBNL1-AS1* reduction with saMB1_1- and saMB1_2-mediated *MBNL1* mRNA upregulation. Black inverted arrowheads in (A) denote samples in which inhibition of the S strand blocked completely (saMB1_1) or partially (saMB1_2) the reduction of *MBNL1-AS1*. Black inverted arrowheads in (B) mark samples in which promotion of the AS strand selection (equivalent to a mismatch within the 3′-most nucleotide of the S strand) blocked completely (saMB1_1) or failed to block (saMB1_2) lncRNA *MBNL1-AS1* reduction. **(C)** The panel to the left shows schematic of dual luciferase assay designed to analyze the off-target potential of the S strand of saMB1_2 by cloning its target sequence into the 3′UTR of luciferase gene to generate the pmirGlo_saMB1_2-S vector. Created in BioRender. Stepniak-Konieczna, E. (2025) https://BioRender.com/j4v7vj1. The panel to the right shows

results of dual luciferase assay in HeLa cells co-transfected for 48 h with pmirGlo-saMB1_2-S and either unmodified or modified versions of saMB1_2. Double black inverted arrowheads mark samples in which active S strand of saMB1_2 inhibited luciferase activity (unmodified saMB1_2 and saMB1_2-5′BIO-AS). Single black inverted arrowhead marks the sample in which inhibition of the S strand by 5′-biotinylation (saMB1_2-5′BIO-S) allowed robust luciferase activity. Transfections of vector alone (no saRNA; striped bar) and saCtrl (grey bar) were used as controls. Results were normalized to Renilla luciferase luminescence and are represented as RLU (relative light unit).

**Supplementary Figure S11.**

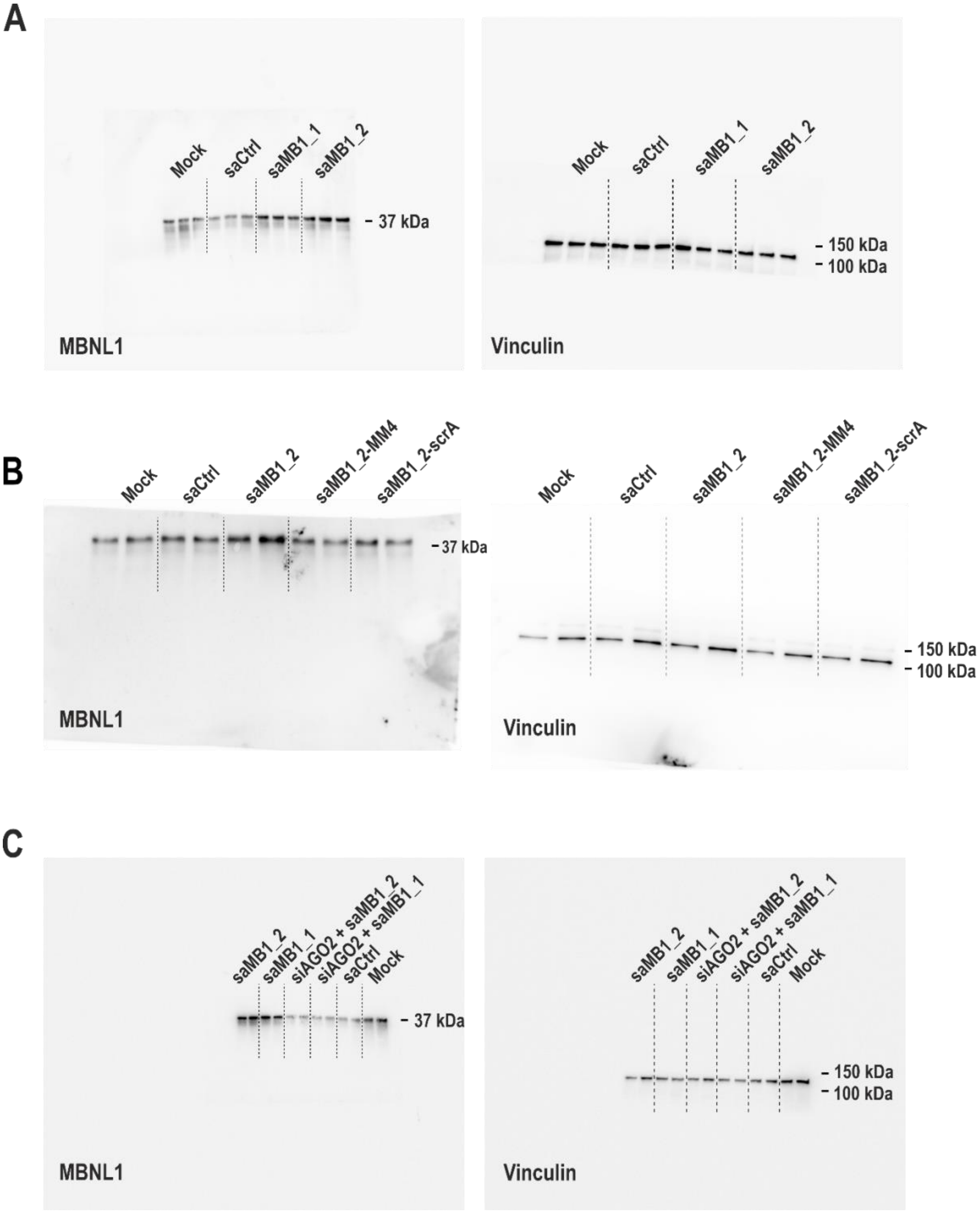


**Supplementary Figure S11. Unadjusted and uncropped western blot images corresponding to Figure 6. (A-C)** Blots corresponding to results shown in Figure 6A, 6B and 6C, respectively. Distinct sample types are described and separated by dotted lines. Each lane represents an individual biological sample.

**Supplementary Figure S12.**

A

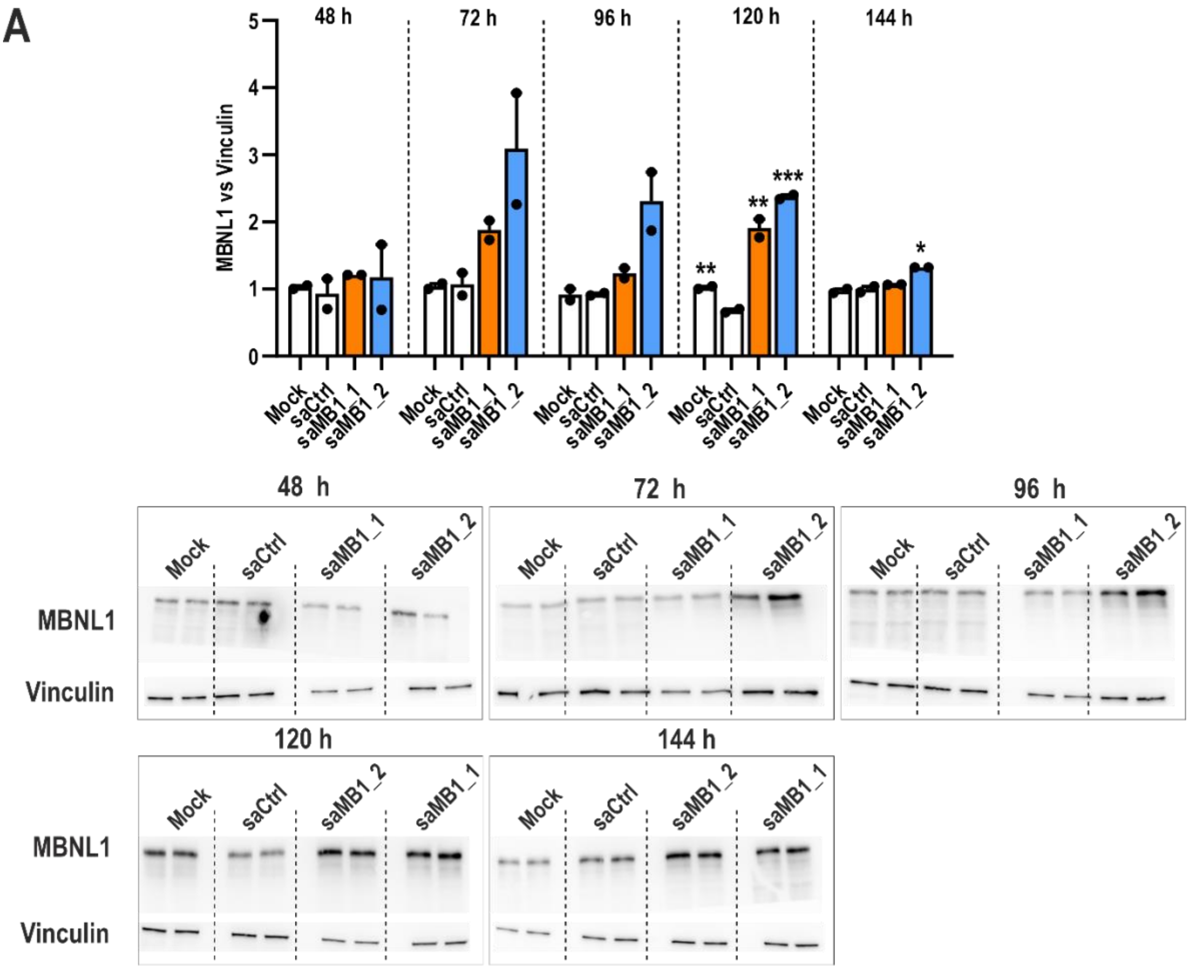


B

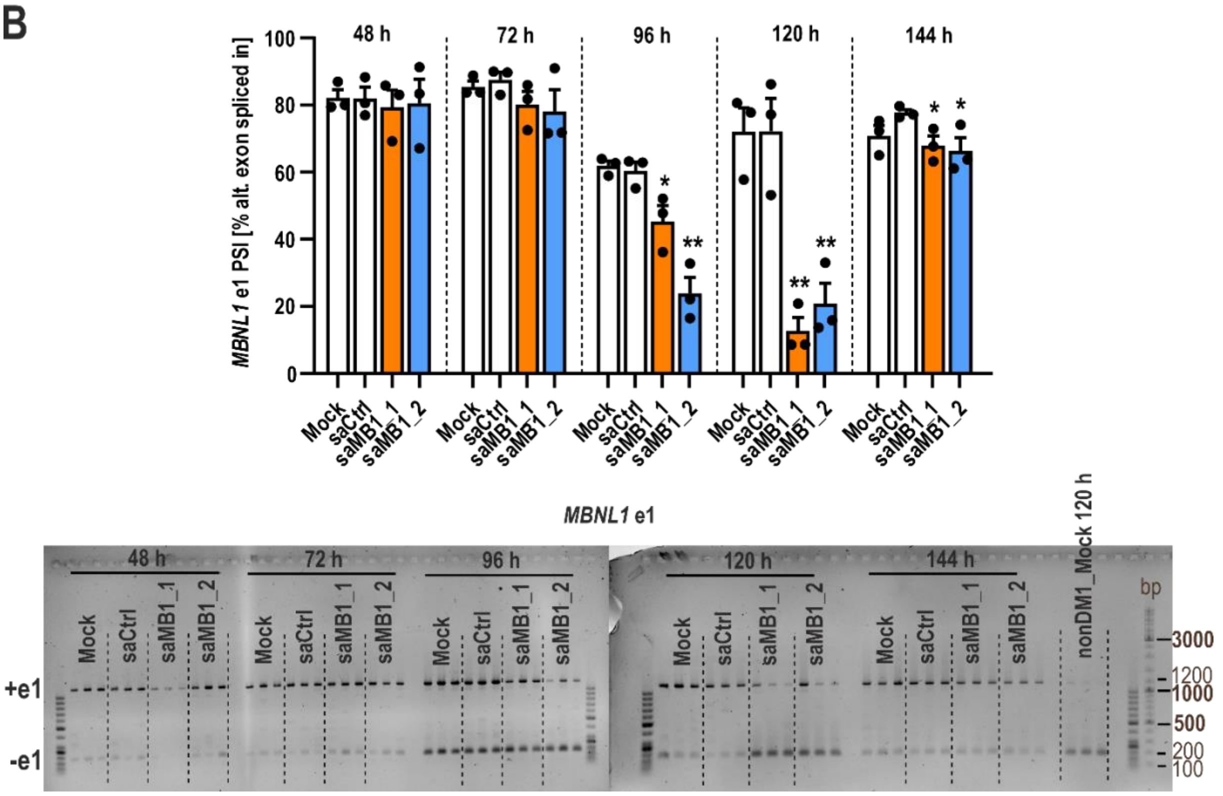

**Supplementary Figure S12. Timecourse analyses show correlation of MBNL1 protein induction upon saRNA treatment with MBNL1-dependent e1 exclusion from *MBNL1* pre-mRNA. (A)** Western blot quantification (top) and corresponding blot images (bottom) of MBNL1 protein levels in GM04033 DM1 fibroblasts transfected with lipofectamine alone (mock) or 75 nM indicated saRNA for indicated timepoints. MBNL1 and Vinculin blots are derived from the same experiment and the same gel. Data are presented as mean ± SEM of duplicate samples. **(B)** RT-PCR-based quantification (top) and corresponding RT-PCR gel images (bottom) of *MBNL1* e1 alternative splicing in GM04033 transfected with lipofectamine alone (mock) or 75 nM indicated saRNA for indicated timepoints.

**Supplementary Figure S13.**

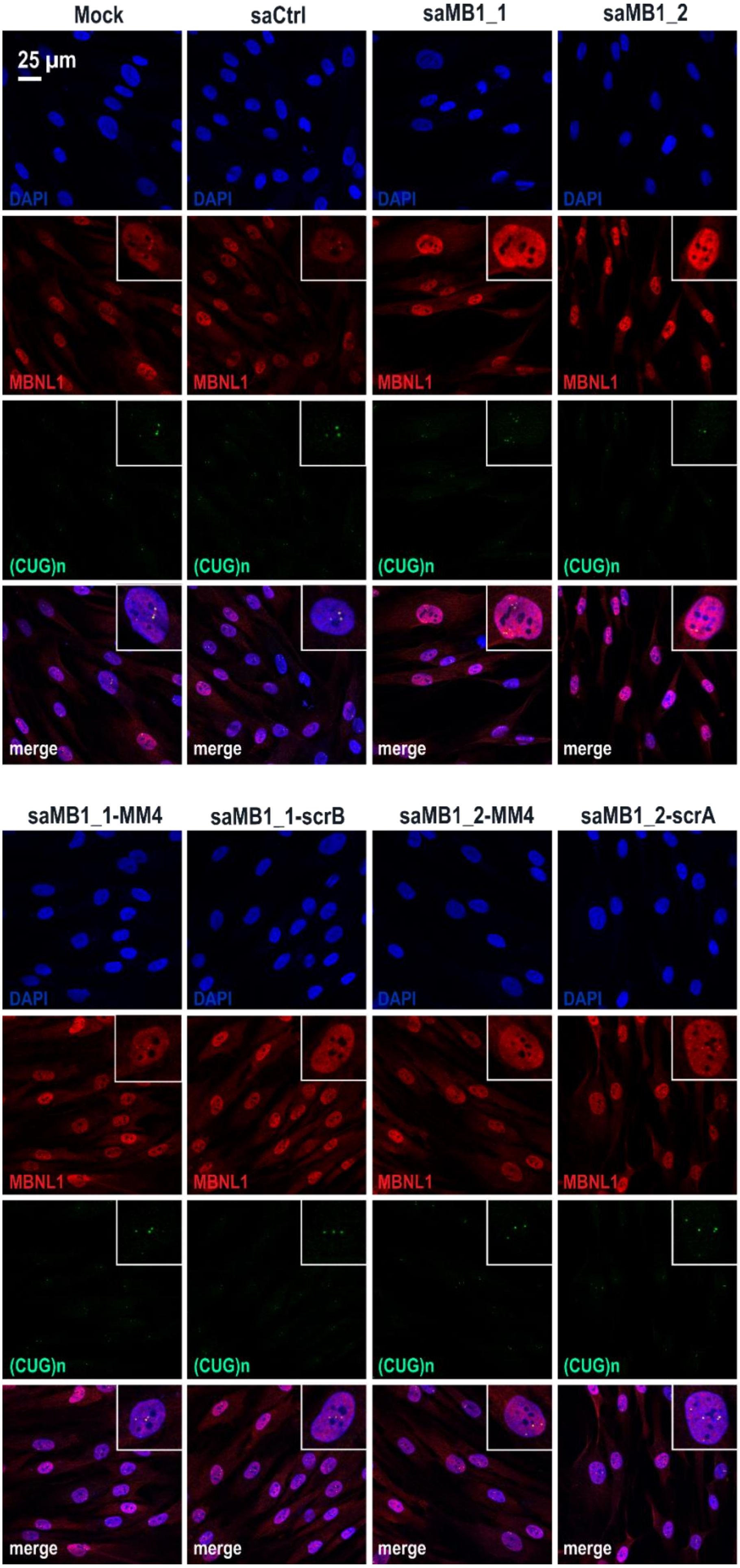

**Supplementary Figure S13. Colocalization of MBNL1 and CUG$^{exp}$ RNA foci in DM1 fibroblasts is not affected by saRNA-mediated *MBNL1* upregulation.** Representative confocal images of RNA-FISH-IF showing MBNL1 (red; DyLight594) and (CUG)n RNA foci (green; Cy3) in GM03989 DM1 fibroblasts transfected with 75 nM indicated saRNA or mock-treated for 120 h. Nuclei were counterstained with DAPI (blue). Scale bar=25 μm.

**Supplementary Figure S14.**

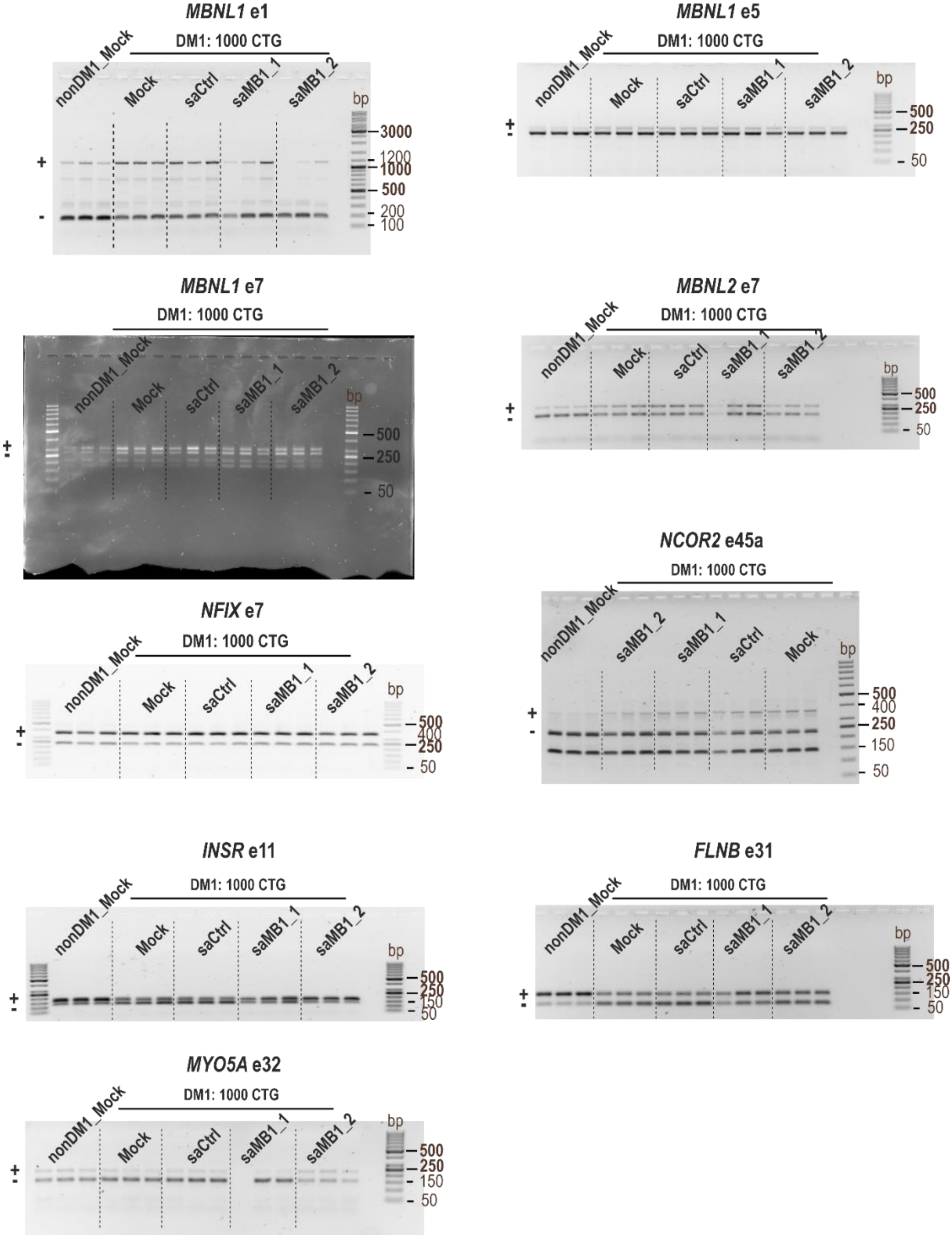


**Supplementary Figure S14. Unadjusted and uncropped RT-PCR gel images of alternative splicing events in DM1 fibroblasts shown in Figure 8A.** Distinct sample types are described and separated by dotted lines. Each lane represents an individual sample. + and – mark alternative exon inclusion and exclusion, respectively.

**Supplementary Figure S15.**

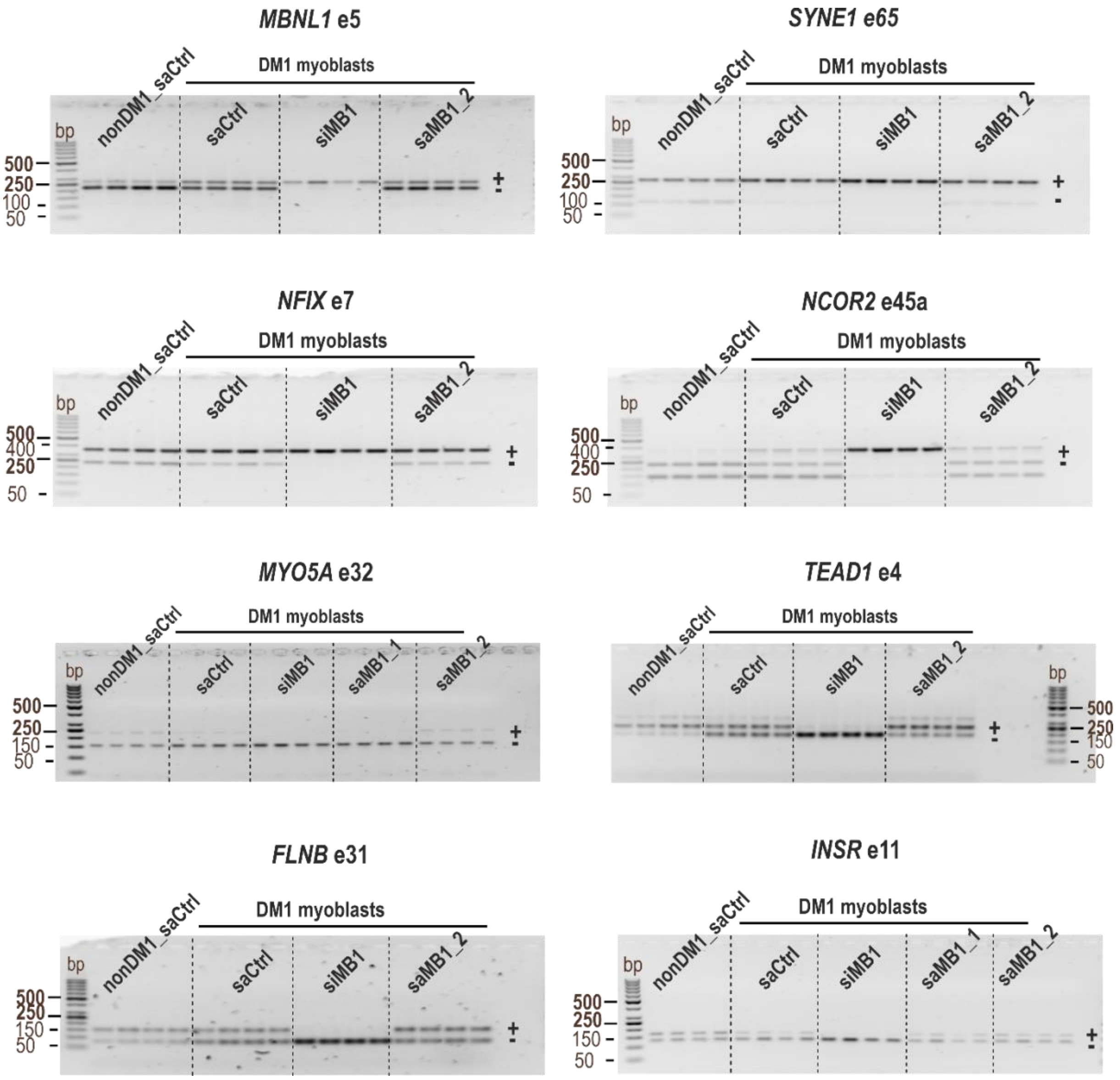


**Supplementary Figure S15. Unadjusted and uncropped RT-PCR gel images of alternative splicing events in DM1 myoblasts shown in Figure 8B.** Distinct sample types are described and separated by dotted lines. Each lane represents an individual sample. + and – mark alternative exon inclusion and exclusion, respectively.

**Supplementary Figure S16.**

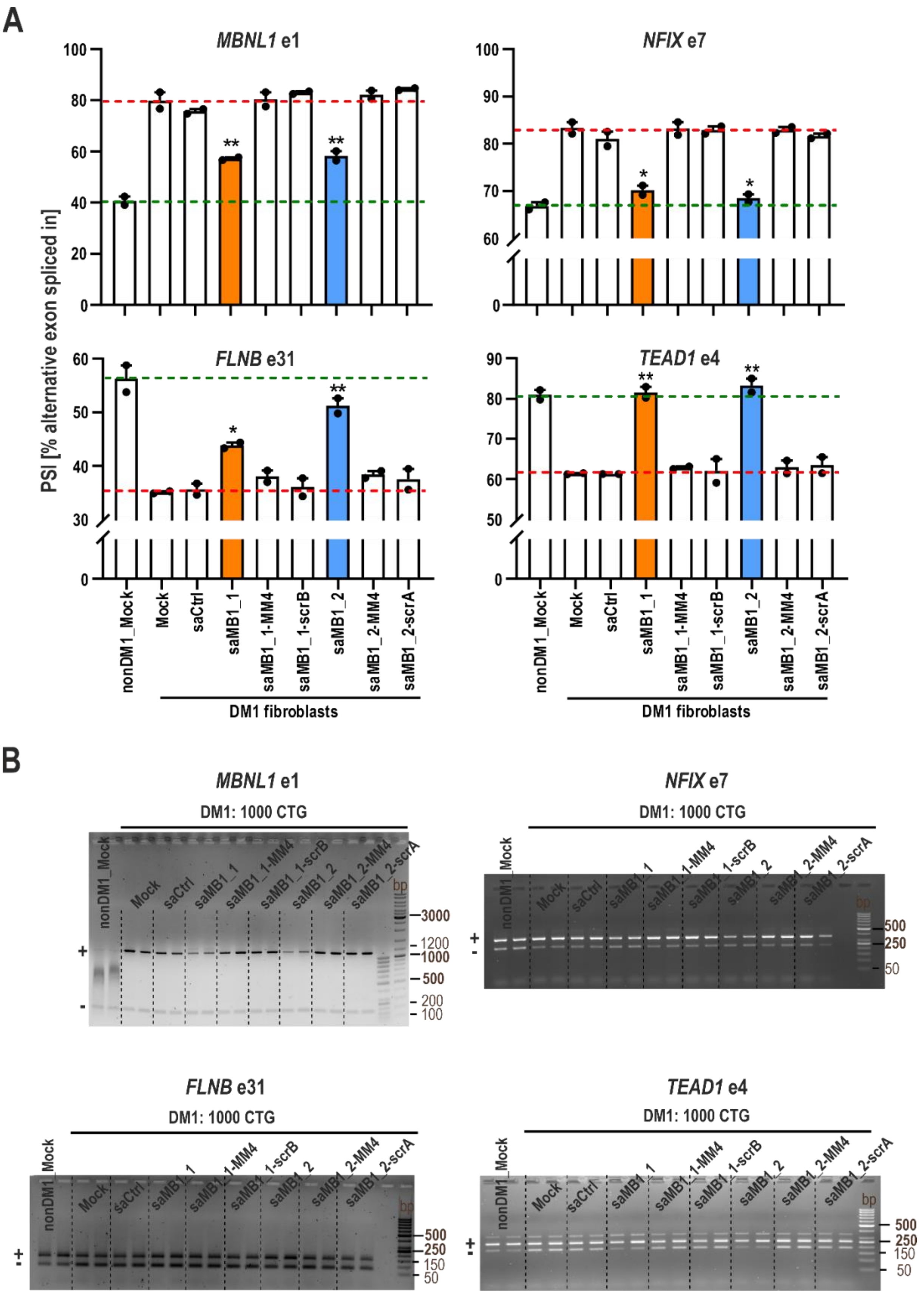


**Supplementary Figure S16. Sequence-scrambled or seed region mutant versions of saMB1_1 and saMB1_2 do not affect *MBNL1*-regulated alternative splicing. (A)** RT-PCR-based quantification of selected splicing events in GM04033 DM1 fibroblasts transfected with 75 nM indicated saRNA for 120 h. NonDM1_Mock represents unaffected and untreated control

fibroblasts. Red and green horizontal dashed lines mark the level of alternative exon inclusion (PSI) in DM1 and unaffected cells, respectively. Data are presented as mean ± SEM of duplicate samples and refer to PSI. **(B)** Representative RT-PCR gel images used for quantification. Lane description is shown, and each lane represents an individual sample. Sample groups are separated by dotted lines. + and – mark PCR products corresponding to the splice isoforms with alternative exon inclusion and exclusion, respectively.

**Supplementary Figure S17.**

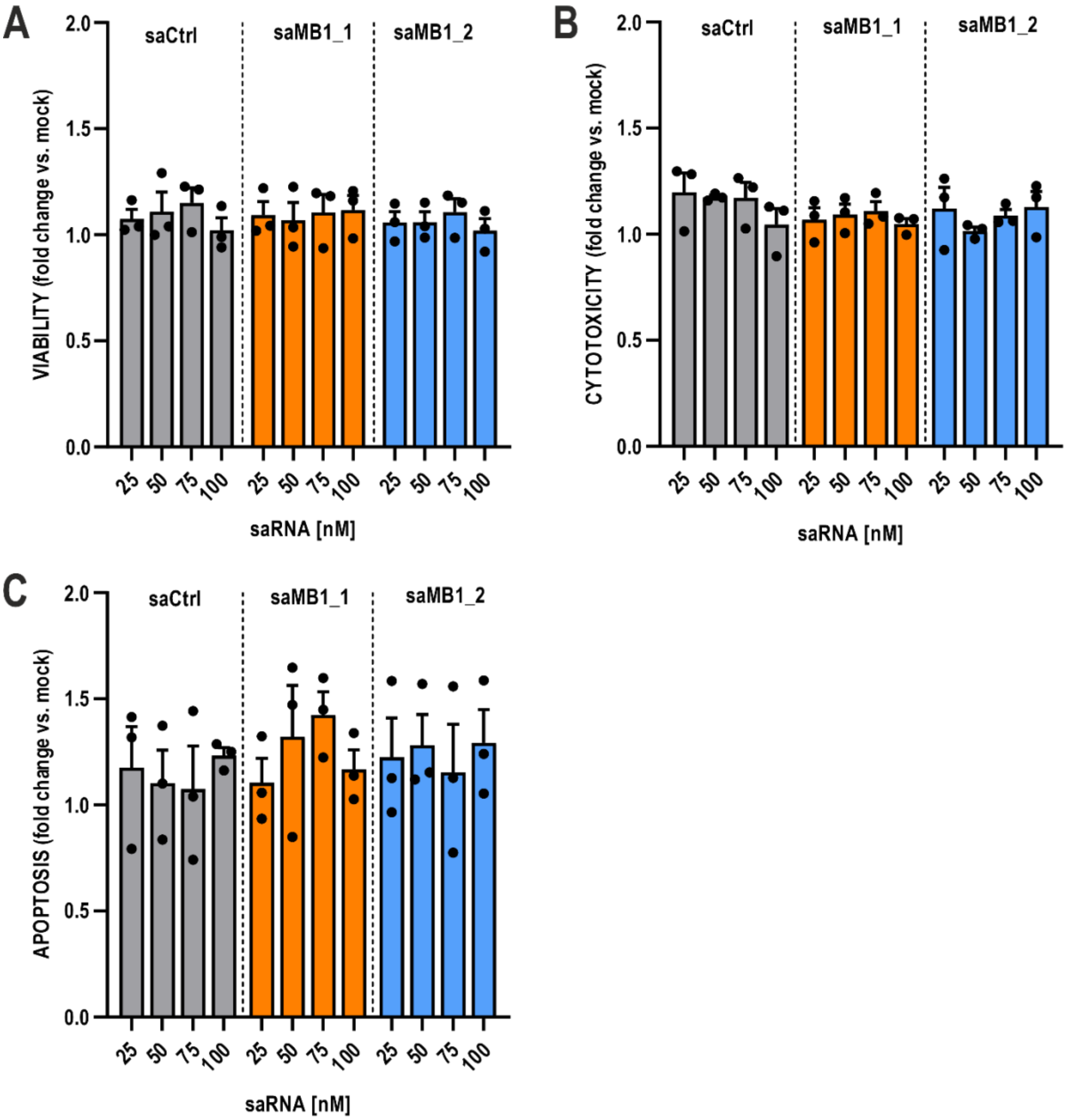


**Supplementary Figure S17. saRNA duplexes do not affect cell viability, toxicity and apoptosis.** Viability **(A),** cytotoxicity **(B)** and apoptosis **(C)** analyses of saRNA dose-response effect at 120 h timepoint in GM04033 DM1 fibroblasts. Results are demonstrated as mean ± SEM of fold change in viability, cytotoxicity, and apoptosis over mock control (untreated cells transfected with lipofectamine alone) of triplicate samples.

## 3. SUPPLEMENTARY TABLES S1-S6

**Supplementary Table S1. Sequences of saRNAs and control RNA duplexes used in this study.** Bases marked red point out single nucleotide mutations introduced within the seed region of saMB1_1 or saMB1_2 duplexes at indicated positions (4,6,8) counted from the 5′ end of the AS strand.

| saRNA | Position of cognate target site relative to TSS2 | Sequence (5' - 3') of sense (S) and antisense (AS) strand of saRNA duplex |
|---|---|---|
| **2-2822** | -2822 | **S:** UAGGCCAAAUCAGUGGUAA [dT][dT]<br>**AS:** UUACCACUGAUUUGGCCUA [dT][dT] |
| **2-2552** | -2552 | **S:** CUCCUCCAGUGUUGAAGUA [dT][dT]<br>**AS:** UACUUCAACACUGGAGGAG [dT][dT] |
| **2-2303** | -2303 | **S:** ACCUGCUAGCAGAUGCAAA [dT][dT]<br>**AS:** UUUGCAUCUGCUAGCAGGU [dT][dT] |
| **2-2250** | -2250 | **S:** ACAGACAAUGGGAGGCAAA [dT][dT]<br>**AS**: UUUGCCUCCCAUUGUCUGU [dT][dT] |
| **2-1652** | -1652 | **S:** CUUUCAAGGCACCAGUUAA [dT][dT]<br>**AS:** UUAACUGGUGCCUUGAAAG [dT][dT] |
| **2-1471** | -1471 | **S:** ACUGGCAUAGUAGGCACUA [dT][dT]<br>**AS:** UAGUGCCUACUAUGCCAGU [dT][dT] |
| **2-1319**<br>**(saMB1_2)** | -1319 | **S:** UGUGGGUACUUGAGUCCUU [dT][dT]<br>**AS:** AAGGACUCAAGUACCCACA [dT][dT] |
| **2-1284** | -1284 | **S:** GGGAAGAGGCACCAUUUAA [dT][dT]<br>**AS:** UUAAAUGGUGCCUCUUCCC [dT][dT] |
| **2-1252** | -1252 | **S:** UCUGGAAGGGAUCAGUGUA [dT][dT]<br>**AS**: UACACUGAUCCCUUCCAGA [dT][dT] |
| **2-1218** | -1218 | **S**: GCUUUGUGGAUCUAGCUAA [dT][dT]<br>**AS**: UUAGCUAGAUCCACAAAGC [dT][dT] |
| **2-942** | -942 | **S:** UCAUCACAUCACAGACGUU [dT][dT]<br>**AS:** AACGUCUGUGAUGUGAUGA [dT][dT] |
| **2-932** | -932 | **S:** ACAGACGUUCAGGAUUCUU [dT][dT]<br>**AS:** AAGAAUCCUGAACGUCUGU [dT][dT] |
| **2-912** | -912 | **S:** CCAUUCAAAGCAAAGCCAA [dT][dT]<br>**AS:** UUGGCUUUGCUUUGAAUGG [dT][dT] |

| | | |
|---|---|---|
| **2-886** | -886 | **S:** CUUAUAGCACCCAGUGAAU [dT][dT]<br>**AS:** AUUCACUGGGUGCUAUAAG [dT][dT] |
| **2-882**<br>**(saMB1_1)** | -882 | **S:** UAGCACCCAGUGAAUUAUG [dT][dT]<br>**AS:** CAUAAUUCACUGGGUGCUA [dT][dT] |
| **2-864** | -864 | **S:** GAUAUGGGGCUGCGGUUUA [dT][dT]<br>**AS:** UAAACCGCAGCCCCAUAUC [dT][dT] |
| **2-806** | -806 | **S:** UCCCGGUAUUUCAGAAGUA [dT][dT]<br>**AS:** UACUUCUGAAAUACCGGGA [dT][dT] |
| **2-769** | -769 | **S:** GGGAGCUUUGCUUUCUCUA [dT][dT]<br>**AS:** UAGAGAAAGCAAAGCUCCC [dT][dT] |
| **2-731** | -731 | **S:** GGGAAUACAGUGGCCUGUA [dT][dT]<br>**AS:** UACAGGCCACUGUAUUCCC [dT][dT] |
| **2-564** | -564 | **S:** UUAGCCCUCCAGGUUCAAA [dT][dT]<br>**AS:** UUUGAACCUGGAGGGCUAA [dT][dT] |
| **2-439** | -439 | **S:** GUGCAAGGGCCUCUUUCAA [dT][dT]<br>**AS:** UUGAAAGAGGCCCUUGCAC [dT][dT] |
| **saRNA** | **Position of cognate target site relative to TSS3** | **Sequence (5' - 3') of sense (S) and antisense (AS) strand of saRNA duplex** |
| **3-3010** | -3010 | **S:** UCCUGACAGCAUUGAUGAA [dT][dT]<br>**AS:** UUCAUCAAUGCUGUCAGGA [dT][dT] |
| **3-2906** | -2906 | **S:** CACGGUAGAUGACUCUUUA [dT][dT]<br>**AS:** UAAAGAGUCAUCUACCGUG [dT][dT] |
| **3-860** | -860 | **S:** CCAUAUGCCAAUAGAGCAA [dT][dT]<br>**AS:** UUGCUCUAUUGGCAUAUGG [dT][dT] |
| **3-847** | -847 | **S:** GAGCAAUUUUGGCUCAUGA [dT][dT]<br>**AS:** UCAUGAGCCAAAAUUGCUC [dT][dT] |
| **3-801** | -801 | **S:** CUGUGUCAUUGCCUCUUUA [dT][dT]<br>**AS:** UAAAGAGGCAAUGACACAG [dT][dT] |
| **3-747** | -747 | **S:** CCUCAUUGGCAACAUUCAA [dT][dT]<br>**AS:** UUGAAUGUUGCCAAUGAGG [dT][dT] |
| **3-653** | -653 | **S:** GUCUUGUGGUGCUGAGUUA [dT][dT]<br>**AS:** UAACUCAGCACCACAAGAC [dT][dT] |

| | | |
|---|---|---|
| **3-650** | -650 | **S:** UCUUGUGGUGCUGAGUUAA [dT][dT]<br>**AS:** UUAACUCAGCACCACAAGA [dT][dT] |
| **3-557** | -557 | **S:** UCACGCUGCUCAAAUCAAA [dT][dT]<br>**AS:** UUUGAUUUGAGCAGCGUGA [dT][dT] |
| **3-498** | -498 | **S:** CUCCCUUGAGGUUGACUUC [dT][dT]<br>**AS:** GAAGUCAACCUCAAGGGAG [dT][dT] |
| **3-461** | -461 | **S:** GACAACACUGUACUCCUUA [dT][dT]<br>**AS:** UAAGGAGUACAGUGUUGUC [dT][dT] |
| **3-430** | -430 | **S:** GACCUCAGCUUCUGCUUUA [dT][dT]<br>**AS:** UAAAGCAGAAGCUGAGGUC [dT][dT] |
| **3-379** | -379 | **S:** AUCUUGCCUGCCUGUGAUA [dT][dT]<br>**AS:** UAUCACAGGCAGGCAAGAU [dT][dT] |
| **3-344** | -344 | **S:** CUGCGGGCUCAAUGGAAUA [dT][dT]<br>**AS:** UAUUCCAUUGAGCCCGCAG [dT][dT] |
| **3-283** | -283 | **S:** UGGGCUUUAGAAGGAAGUG [dT][dT]<br>**AS:** CACUUCCUUCUAAAGCCCA [dT][dT] |
| **3-252** | -252 | **S:** CAGGAGAAUGUACCAUUUG [dT][dT]<br>**AS**: CAAAUGGUACAUUCUCCUG [dT][dT] |
| **3-234** | -234 | **S:** GUAAACACCCCUUUCCUUU [dT][dT]<br>**AS:** AAAGGAAAGGGGUGUUUAC [dT][dT] |
| **3-145** | -145 | **S:** GCUUGGAAGUCAGCUGCAA [dT][dT]<br>**AS:** UUGCAGCUGACUUCCAAGC [dT][dT] |
| **control saRNA** | **Additional information** | **Sequence (5' - 3') of sense (S) and antisense (AS) strand of saRNA duplex** |
| **saCtrl** | non-targeting control | **S**: ACUACUGAGUGACAGUAGA [dT][dT]<br>**AS:** UCUACUGUCACUCAGUAGU [dT][dT] |
| **saMB1_1_MM4** | mutation within the seed region of saMB1_1 at position **4** | **S**: UAGCACCCAGUGAAU**C**AUG [dT][dT]<br>**AS:** CAU**G**AUUCACUGGGUGCUA [dT][dT] |
| **saMB1_1_MM6** | mutation within the seed region of saMB1_1 at position **6** | **S**: UAGCACCCAGUGA**C**UUAUG [dT][dT]<br>**AS:** CAUAA**G**UCACUGGGUGCUA [dT][dT] |
| **saMB1_1_MM8** | mutation within the seed region of saMB1_1 at position **8** | **S**: UAGCACCCAGU**U**AAUUAUG [dT][dT]<br>**AS:** CAUAAUU**A**ACUGGGUGCUA [dT][dT] |

| | | |
|---|---|---|
| **saMB1_2_MM4** | mutation within the seed region of saMB1_2 at position **4** | **S**: UGUGGGUACUUGAGU**U**CUU [dT][dT]<br>**AS:** AAG**A**ACUCAAGUACCCACA [dT][dT] |
| **saMB1_2_MM6** | mutation within the seed region of saMB1_2 at position **6** | **S**: UGUGGGUACUUGA**U**UCCUU [dT][dT]<br>**AS:** AAGGA**A**UCAAGUACCCACA [dT][dT] |
| **saMB1_2_MM8** | mutation within the seed region of saMB1_2 at position **8** | **S**: UGUGGGUACUU**U**AGUCCUU [dT][dT]<br>**AS:** AAGGACU**A**AAGUACCCACA [dT][dT] |
| **saMB1_1_scrA** | scrambled sequence of saMB1_1 | **S**: ACAAUUGAAGGCUCCUGUA [dT][dT]<br>**AS:** UACAGGAGCCUUCAAUUGU [dT][dT] |
| **saMB1_1_scrB** | scrambled sequence of saMB1_1 | **S**: AAGAGCUGGUCCUACAUAU [dT][dT]<br>**AS:** AUAUGUAGGACCAGCUCUU [dT][dT] |
| **saMB1_1_scrC** | scrambled sequence of saMB1_1 | **S**: CUAUAGACAUGUACGGUAC [dT][dT]<br>**AS:** GUACCGUACAUGUCUAUAG [dT][dT] |
| **saMB1_2_scrA** | scrambled sequence of saMB1_2 | **S**: GCGAUUUCCUGUAGUUGGU [dT][dT]<br>**AS:** ACCAACUACAGGAAAUCGC [dT][dT] |
| **saMB1_2_scrB** | scrambled sequence of saMB1_2 | **S**: GUGCAGUAUCGGUUCUGUU [dT][dT]<br>**AS:** AACAGAACCGAUACUGCAC [dT][dT] |
| **saMB1_2_scrC** | scrambled sequence of saMB1_2 | **S**: ACUCCGUUUUGGGGUAUUG [dT][dT]<br>**AS:** CAAUACCCCAAAACGGAGU [dT][dT] |
| **saRNA** | **Position of cognate target site relative to TSS** | **Sequence (5' - 3') of sense (S) and antisense (AS) strand of saRNA duplex** |
| **saP21** | -322 | **S:** CCAACUCAUUCUCCAAGUA[dT][dT]<br>**AS:** UACUUGGAGAAUGAGUUGG[dT][dT] |

**Supplementary Table S2. Sequences of chemically modified and mismatch-containing saRNA duplexes for strand inhibition or promotion.** Bases marked red point out a single mismatched base introduced in the 3′ end of the indicated strands. Abbreviations: BIO – Biotin; MM – mismatch; S – sense strand; AS – antisense strand.

| **saRNA** | **Sequence (5' - 3') of sense (S) and antisense (AS) strand of saRNA duplex** |
|---|---|
| **saMB1_1 5′BIO-S** | **S: BIO**-UAGCACCCAGUGAAUUAUG [dT][dT]<br>**AS:** CAUAAUUCACUGGGUGCUA [dT][dT] |
| **saMB1_1 5′BIO-AS** | **S:** UAGCACCCAGUGAAUUAUG [dT][dT]<br>**AS: BIO**-CAUAAUUCACUGGGUGCUA [dT][dT] |
| **saMB1_2_5′BIO-S** | **S: BIO**-UGUGGGUACUUGAGUCCUU [dT][dT]<br>**AS:** AAGGACUCAAGUACCCACA [dT][dT] |
| **saMB1_2_5′BIO-AS** | **S:** UGUGGGUACUUGAGUCCUU [dT][dT]<br>**AS: BIO**-AAGGACUCAAGUACCCACA [dT][dT] |
| **saMB1_1_MM-S** | **S:** UAGCACCCAGUGAAUUAUG [dT][dT]<br>**AS:** CAUAAUUCACUGGGUGCU**U** [dT][dT] |
| **saMB1_1_MM-AS** | **S:** UAGCACCCAGUGAAUUAU**C** [dT][dT]<br>**AS:** CAUAAUUCACUGGGUGCUA [dT][dT] |
| **saMB1_2_MM-S** | **S:** UGUGGGUACUUGAGUCCUU [dT][dT]<br>**AS:** AAGGACUCAAGUACCCAC**U** [dT][dT] |
| **saMB1_2_MM-AS** | **S:** UGUGGGUACUUGAGUCCU**A** [dT][dT]<br>**AS:** AAGGACUCAAGUACCCACA [dT][dT] |

**Supplementary Table S3. siRNA and GapmeR sequences.** Abbreviations: S-sense strand; AS-antisense strand; (*) phosphorothioate modifications; The Antisense LNA GapmeRs contain a central DNA part flanked by LNA; the position of the LNA modification is not shown (information proprietary to Qiagen).

| siRNA or Antisense LNA GapmeR / target | Sequence (5' - 3') |
|---|---|
| siAGO2 | **S:** GCACGGAAGUCCAUCUGAA [dT][dT]<br>**AS:** UUCAGAUGGACUUCCGUGC [dT][dT] |
| siCTR9 | **S:** GCACGUAUAGAUGGCAAUU [dT][dT]<br>**AS:** AAUUGCCAUCUAUACGUGC [dT][dT] |
| siRHA | **S:** GCACGAGAACAUGGAUCAA [dT][dT]<br>**AS:** UUGAUCCAUGUUCUCGUGC [dT][dT] |
| siMEIS1 | **S:** CUGACAUUCAGGCCCAAGU [dT][dT]<br>**AS:** ACUUGGGCCUGAAUGUCAG [dT][dT] |
| siMEIS2 | **S:** CACCCUGGAAUGACUAUGU [dT][dT]<br>**AS:** ACAUAGUCAUUCCAGGGUG [dT][dT] |
| siMBNL1 | **S:** CACUGGAAGUAUGUAGAGA [dT][dT]<br>**AS:** UCUCUACAUACUUCCAGUG [dT][dT] |
| siCtrl | Proprietary sequence<br>(Ambion™ *Silencer*™ Select Negative Control #2) |
| siMBNL1-AS1 Lincode SMARTPool siRNA | **AS:** CUUCUGAAAUACCGGGAUA<br>**AS:** GCUUAGAAUGCCUGCGAAC<br>**AS:** CCAAACAUUUCUACGUAGU<br>**AS:** AGACUAGAAUCCUGCGAUC |
| Antisense LNA GapmeR *MBNL1-AS1* | G*C*A*A*G*T*A*G*C*A*A*G*C*A*A*T |
| Antisense LNA GapmeR *DMPK* | A*A*A*T*G*C*G*C*A*G*C*T*A*A*G*C |

**Supplementary Table S4. Expression primers for RT-qPCR.**

| Transcript name | Primers Sequence (5' - 3') |
|---|---|
| *MBNL1* (mRNA) | CTGCCGAACATCTGACTAGC<br>TTGTGTGTGTTGCTTGACGA |
| *MBNL1* (pre-mRNA) | GTGTGTGTGTGTAGGCCAAC<br>GCTAGTCAGATGTTCGGCAG |
| *MBNL1* (TSS2-derived transcripts) | CCACAATGCTCCCATGACAA<br>CCCCACTGTCGACCAAGT |
| *MBNL1* (TSS3-derived transcripts) | AGGCGCGACCTTTCATAAG<br>CTGCTCTAGAAGCGGTTCCA |
| *lncRNA MBNL1-AS1* | TGGATAAGACAGTCCCTACA<br>ATTGGATTGCTTCCCACATA |
| *MBNL2* | CCAAGGGCAGGTTCGATTCT<br>AAGCTGGATGAAGTCTGGCA |
| *DMPK* | CACTGTCGGACATTCGGGAAGGTGC<br>GCTTGCACGTGTGGCTCAAGCAGCTG |
| *AGO2* | CGCGTCCGAAGGCTGCTCTA<br>TGGCTGTGCCTTGTAAAACGCT |
| *CTR9* | TTACCGGAGGGAGATGAAG<br>AACTCTTCTGTTTTTCCTTGC |
| *RHA* | ACCTGCTATCATCAGCCAGTTGGA<br>TCCATTGTCGTATCGGGCCATCTT |
| *MEIS1* | GTTGAAGTAGGAAGGGAGCCAG<br>GCTGTGTGCGGGTACTGATG |
| *MEIS2* | GTGAGCCAAGGAGCAGCATA<br>ACATGTAGTGCCATTGCCCA |
| *GAPDH* | TGAAGGTCGGAGTCAACGGA<br>GATGACAAGCTTCCCGTTCTC |
| *GAPDH (pre-mRNA*, used for Click-IT assay*)* | AATCCCATCACCATCTTCCAG<br>GAGCCACACCATCCTAGTTG |

**Supplementary Table S5. Alternative splicing primers.**

| Transcript name | Alternative exon | Primers Sequence (5' - 3') |
|---|---|---|
| *MBNL1* | e1 | TCATGACTCCCACAATGCCT<br>ATGGCCATGTTCTTCTGCTG |
| *MBNL1* | e5 | GCTGCCCAATACCAGGTCAAC<br>TGGTGGGAGAAATGCTGTATGC |
| *MBNL1* | e7 | ACCAACAGGCTCTAGCCAACATGC<br>CGTCCTTTACTCTAACCAAGC |
| *MBNL2* | e7 | TCCTTTACCAAAGAGACAAGCAC<br>CTCAATGCAGATTCTTGGCATTCC |
| *NCOR2* | e45a | ACACCCACAACCGGAATGAGCCTG<br>GGACTTGGCTTTTCGGCTGCTG |
| *NFIX* | e7 | GAGCCCTGTTGATGACGTGTTCTA<br>CTGCACAAACTCCTTCAGTGAGTC |
| *SYNE1* | e65 | GGTCCATGAAAGCAGCAATC<br>ACTGATTGTGTTCTGCAACG |
| *INSR* | e11 | CCAAAGACAGACTCTCAGAT<br>AACATCGCCAAGGGACCTGC |
| *FLNB* | e31 | GCTTCGGTGGTGTTGATATTC<br>GTCACTCACTGGGACATAGG |
| *MYO5A* | e32 | GAACAACCGACAGCAGCAG<br>TTACGGACCGTCTTATCCTG |
| *TEAD1* | e4 | GGAGGCCCTGGCTATCTATC<br>GCTTGGAATGAAAATCACGAG |

**Supplementary Table S6. CUT&RUN primers for qPCR.**

| PCR product name (position of PCR product relative to TSS2 of *MBNL1*) | Primers Sequence (5' - 3') |
|---|---|
| *MBNL1* (+4058;+4132) | CGTGGGGAGGTTAGCTAGTT<br>ACAGACTCAAGATGGCACCA |
| *MBNL1* (+1104;+1177) | CCTCGCACAGCACTTTACAA<br>GCCCCAGAGAGCCTTAAGAA |
| *MBNL1* (+231;+310) | GAAGGAGGAAGTGCGAGGAG<br>CGAAACCAACTACCGCGAG |
| *MBNL1* (-14;+28) | GCGATAAGAGGCTGCACAG<br>CCACAACTCATTCAGCTGCA |
| *MBNL1* (-912; -841) | CCATTCAAAGCAAAGCCAAGT<br>CAAATAAACCGCAGCCCCAT |
| *MBNL1* (-1097; -1022) | TCAACAAAACCGAACCTGCA<br>CGTTTGTCCCATGTTTGTTGG |
| *MBNL1* (-1655; -1565) | CACCTTTCAAGGCACCAGTT<br>TGTATGTGGGAAGCAATCCA |
| *MBNL1* (-3465; -3403) | GCTATGAATGTGGTATGCAGAAG<br>GTAAGTGTGTGAGTGCCTGTG |
| **PCR product name (position of PCR product relative to TSS of *p21*$^{WAF1/CIP1}$)** | **Primers Sequence (5' - 3')** |
| *P21* (+5469;+5606) | GACTGTGATGCGCTAATGG<br>AGGTAGAGCTTGGGCAGG |
| *P21* (+735;+888) | GAACGGACTGTATGAGGTCAG<br>CAGTGCCCGGCTTCC |
| *P21* (+107;+235) | CAGAGCCGAGCCAAGC<br>CACCGACCCACGCCC |
| *P21* (-1;+101) | TGCCGAAGTCAGTTCCTTG<br>GGTCCCCTGTTGTCTGC |
| *P21* (-141;+22) | AGCCAGGAGCCTGGGC<br>CAAGGAACTGACTTCGGCA |
| *P21* (-417; -262) | CAGCTGCATTGGGTAAATCC<br>GACACATTTCCCCACGAAGT |

| | |
|---|---|
| *P21* (-1668; -1222) | GAGAAGGTGCCTGTCCTCTG<br>TCTGTGCCTGAAACATTTGC |
| *P21* (-3861; -3777) | TCAATGCCACCACCTTAACA<br>CAGAGCCAGGATGAATTGGT |

## 4. SUPPLEMENTARY REFERENCES

REFERENCES


1. Barski, A., Cuddapah, S., Cui, K., Roh, T.Y., Schones, D.E., Wang, Z., Wei, G., Chepelev, I. and Zhao, K. (2007) High-resolution profiling of histone methylations in the human genome. *Cell*, **129**, 823-837.
